# Visualizing nationwide variation in medicare Part D prescribing patterns

**DOI:** 10.1186/s12911-018-0670-2

**Published:** 2018-11-19

**Authors:** Alexander Rosenberg, Christopher Fucile, Robert J. White, Melissa Trayhan, Samir Farooq, Caroline M. Quill, Lisa A. Nelson, Samuel J. Weisenthal, Kristen Bush, Martin S. Zand

**Affiliations:** 1 Rochester Center for Health Informatics at the University of Rochester Medical Center, 265 Crittenden Blvd - 1.207, Rochester, 14642 NY USA; 2 University of Alabama Birmingham, Düsternbrooker Weg 20, Birmingham, 14642 AL USA; 3 Department of Medicine, Division of Nephrology, University of Rochester Medical Center, 601 Elmwood Avenue, Rochester, 14642 NY USA; 4 Department of Medicine, Division of Pulmonary and Critical Care, University of Rochester Medical Center, 601 Elmwood Avenue, Rochester, 14642 NY USA; 5 Clinical and Translational Science Institute, University of Rochester Medical Center, 265 Crittenden Blvd, Rochester, 14642 NY USA; 6 Department Pharmacy, University of Rochester Medical Center, 601 Elmwood Avenue, Rochester, 14642 NY USA

**Keywords:** Medicare, Prescribing, Machine learning, t-SNE, Healthcare variation

## Abstract

**Background:**

To characterize the regional and national variation in prescribing patterns in the Medicare Part D program using dimensional reduction visualization methods.

**Methods:**

Using publicly available Medicare Part D claims data, we identified and visualized regional and national provider prescribing profile variation with unsupervised clustering and t-distributed stochastic neighbor embedding (t-SNE) dimensional reduction techniques. Additionally, we examined differences between regionally representative prescribing patterns for major metropolitan areas.

**Results:**

Distributions of prescribing volume and medication diversity were highly skewed among over 800,000 Medicare Part D providers. Medical specialties had characteristic prescribing patterns. Although the number of Medicare providers in each state was highly correlated with the number of Medicare Part D enrollees, some states were enriched for providers with > 10,000 prescription claims annually. Dimension-reduction, hierarchical clustering and t-SNE visualization of drug- or drug-class prescribing patterns revealed that providers cluster strongly based on specialty and sub-specialty, with large regional variations in prescribing patterns. Major metropolitan areas had distinct prescribing patterns that tended to group by major geographical divisions.

**Conclusions:**

This work demonstrates that unsupervised clustering, dimension-reduction and t-SNE visualization can be used to analyze and visualize variation in provider prescribing patterns on a national level across thousands of medications, revealing substantial prescribing variation both between and within specialties, regionally, and between major metropolitan areas. These methods offer an alternative system-wide and pattern-centric view of such data for hypothesis generation, visualization, and pattern identification.

**Electronic supplementary material:**

The online version of this article (10.1186/s12911-018-0670-2) contains supplementary material, which is available to authorized users.

## Background

Pharmaceutical spending accounts for 5-25% of total medical care expenditures in Europe, and 16% of all Medicare expenditures in the United States. Variation in prescribing patterns is common, even within groups of providers with a similar scope of practice and patient mix. Prescribing variation may be due to provider preferences, patient case-mix, deviation from practice guidelines, insurance formulary restrictions, and occasionally fraud [1–5]. Understanding patterns of prescribing variation is critical to improving healthcare delivery. Visualizing prescribing variation in ways that accurately reflect underlying data structure can be challenging. Good data visualization can provide a “big picture” of complex data, especially variation and quantitative changes in large and complex data sets [6–8]. In this manuscript, we apply non-linear visualization methods to Medicare Part D provider prescribing data to evaluate patterns at the level of *collections* of prescriptions, as opposed to a univariate, per-medication approach. This reveals substantial provider variation at the local, regional and national levels, even when controlled for provider specialty and medication volumes.

Prescription claims data capture the volume, diversity and cost of medications prescribed by individual providers. For example, the 2013 Medicare Part D prescribing pattern data set consists of 1,049,381 providers and 3449 prescription drugs [9]. Because the claims are linked to thousands of individual provider treatment decisions, their patterns are an objective measure of how medical care is actually delivered. They quantify a pattern of medical practice within the population a provider treats. Lists of medications and associated claim volumes per provider, termed feature vectors, can be used to cluster providers with similar prescribing patterns. Cluster membership can then be compared to independent data such as geographic location, medical specialty, patient case mix or outcomes. Unsupervised clustering methods are very efficient at classifying data with hundreds or thousands of features, particularly when the gold-standard or ground-truth for cluster membership is unknown (e.g. how providers should be grouped).

Pattern recognition in high-dimensional data, such as large prescribing claims data sets, is difficult. Thus, visualizations that accurately reflect feature variation in high dimensional data are extremely useful for data exploration, inference and decision making [6, 7, 10]. Standard visualization methods for high dimensional data use classical multidimensional scaling [11] or Principal Components Analysis (PCA) [12]. These methods involve linear transformations that project multidimensional data into two or three dimensions, while preserving relative distances between data points. When applied to very high dimensional data, however, PCA and other linear transformation methods often result in dense visualizations that can overwhelm subtle sub-groupings and do little to highlight patterns in the underlying data.

Recently, van der Maaten and colleagues developed t-distributed stochastic neighbor embedding (t-SNE) [13], a non-linear mapping and dimension reduction method that balances cluster display at the local and global levels. This makes t-SNE is ideally suited to visualizing medication prescribing pattern variation for very large data sets. t-SNE has been used to improve visualization of patterns in single nucleotide polymorphisms [14], single-cell RNAseq analysis [15], drug synergy interactions [16], prognostic tumor markers [17], and electronic medical record data [18].

Variation of regional prescribing practices has important implications for behavioral, economic, and healthcare outcomes [19, 20]. To our knowledge, there are currently no published analyses that examine and visualize geographic variations in drug co-prescribing patterns at a national level, based on collections of medications, at a national level, irrespective of provider specialty. Regional variation in health *services delivery* has been well described [21–27]. In contrast, little is known about regional *patterns* of prescription drug utilization beyond focused studies of prescribing patterns for antibiotics [1], chemotherapy [28], cholinesterase therapy [29], psychiatric medications [30], and statin cholesterol lowering agents [31]. In these studies, patterns have been found to reflect the nature and complexity of health status of patient populations [32, 33], patient socioeconomic factors [34–37], provider preferences with self-reinforcing regional influences [38–40], social network influence (i.e. “prescriber contagion”) [41], and composition of specialties and Medicare formulary [40].

The focus of this work is twofold. First, t-SNE is used to visualize the prescribing patterns of Medicare Part D providers based on the volumes and types of medication claims, and unsupervised agglomerative clustering is used to validate groupings of providers identified by t-SNE. Second, we identify and visualize regional prescribing pattern differences among Medicare Part D providers across specialties, and variations in the prescribing patterns across medical specialties, states, and geographic regions in the United States. That such variations exist is not surprising. The innovation here is that an entire national healthcare data set with hundreds of thousands of providers, millions of patients, and thousands of drugs, can be visualized in a way that identifies prescribing patterns linked to practitioner specialty and regional variation.

## Methods

### Medicare Part D data

Medicare Part D 2013 provider prescribing data were downloaded directly from the Center for Medicare Services (CMS) [9]. A provider refers to any individual who is licensed to prescribe medications and appears in the data set. The data were packaged as three files: 1) a table of providers and their associated annotations, including their unique national provider identifier (NPI), address, summary statistics on numbers of claims, costs, etc.; 2) a table of drugs and their associated annotations including flags for whether they are narcotics, DEA schedule II or III, or categorized as Beers (medications to avoid in older adults [42]), as well as summary statistics (e.g. numbers of claims, costs, etc.); and 3) a table of NPI, drug (both brand and generic names, which taken together are unique) and the number of claims, duration of prescription, and cost for each provider-drug combination. This third file represents a bipartite graph specifying connections between disjoint sets of nodes (i.e. providers and drugs) that are linked by a corresponding measure (e.g. number of claims). To comply with data privacy requirements, values in the provider-by-drug matrix less than 11 were set to 0 by CMS prior to data release [43]. All formatted data were imported into Matlab R2016a (Mathworks, Natick MA) or Mathematica 11.1 (Wolfram, Champaign IL) for further analysis and visualization.

### Feature vector construction

For analysis, a feature vector was created for each provider *Ω*_*i*_={*α*_*i*,1_,*α*_*i*,2_...*α*_*i*,*m*_} where *i* is the provider number and *α*_*i*,*j*_ is the number of Medicare outpatient prescription claims for drug *α*_*j*_ attributed to provider *i*. The total number of providers is designated by *n*, and the total number of individual drugs by *m*. A restriction of the data set, implemented by CMS to ensure non-identifiability of Medicare recipients, is that if *α*_*i*,*j*_≤ 11, then *α*_*i*,*j*_=0. With this constraint, the summary number of claims associated with a particular provider (or drug) in the CMS data set may not be exactly equivalent to the sum of the provider-by-drug matrix. Thus, while there were 1,049,381 providers and 3449 drugs in the data set, there were only 808,020 providers with ≥ 11 claims for at least one drug. Similarly, there were 2892 drugs with ≥ 11 claims from at least one provider.

### Supporting data sources

Additional file 1: Figure S1 shows a schema of the data sets used for this study, which are all publicly available. The number of Medicare Part D participants by state were obtained from CMS public use files (boxes 1, 2, and 3) [44]. To collapse individual drugs into categories, we used the National Drug File from the Veterans Administration [45], followed by further, minor manual aggregation to result in 198 drug categories (Additional file 1: Figure S1, box 4). For some analyses, we consider providers practicing in 52 metropolitan areas with a population ≥ 1,000,000 by the July 2012 Core-Based Statistical Areas (CBSAs) estimate [46]. We link CBSAs to county and Federal Information Processing Standards (FIPS) codes using a look-up table from the National Bureau of Economic Research (box 8) [47]. We linked providers to their FIPS county codes using a table from the U.S. Department of Housing and Urban Development website (box 5) [48]. Finally, we obtained population estimates of Medicare Part D enrollees by county from the Kaiser Family Foundation website [49], where we consider both Medicare Advantage and the Prescription Drug Plan (box 7) enrollees.

### Visualization, clustering, and statistical methods

Providers with similar prescribing patterns were identified by agglomerative clustering implemented in Wolfram Mathematica. Ward’s minimum variance criteria, which minimizes the total within-cluster variance [50], was used to determine cluster membership and number. Clusters were also grouped by provider geographical region, state, and medical specialty. Visualization of providers based on their prescribing patterns, we used the fast t-distributed stochastic neighbor embedding (t-SNE) dimension reduction method of van der Maaten and Hinton [13]. Given the size of the data set, with > 10^5^ providers, we used the fast Barnes-Hut implementation of t-SNE in Matlab [51] with 50 initial dimensions based on principal component analysis pre-processing to improve computational efficiency. Unlike with clustering methods, there are no accepted standards for selecting t-SNE visualization hyperparameters, although such guidelines have been suggested [13, 51, 52]. We selected hyperparameter values within the range suggested by van der Maatan et al. [13, 51, 52] based on the data set size and parameter numbers, computational efficiency, t-SNE algorithm convergence, and final embeddings that minimized the cost-function. Reproducibility of the t-SNE visualization results was accomplished by fixing the pseudo-random number generator seed parameter.

### Sensitivity analysis and dimensional reduction

We performed sensitivity analysis by varying initial PCA dimensions as well as perplexity and selected parameters that both minimized t-SNE cost and resulted in visual clarity of the embedding. For the visualizations used in this manuscript, we used a perplexity of 40, and *theta*=0.5. The algorithm performed 300-1500 iterations per run and we selected the result with the minimum t-SNE cost function (error rate) [13]. Dimensional reduction to visualize the CBSA groupings CBSAs was accomplished using classical multidimensional scaling [11] implemented in Matlab using a CBSA-CBSA distance matrix with one minus correlation as the metric. Comparisons of the differences in proportion of provider fractions between geographic regions was performed using the Mann-Whitney U test.

### Measures of skewness

We used the bootstrap implementation of the Gini index [53–55], to quantify skewness of the claims distributions. The Gini index was calculated using the formula:

$$G = \frac{\sum_{i=1}^{n} (2i - n - 1)x_{i}}{n^{2} \mu} $$

where *n* is the number of observations (e.g. providers, drugs), *x*_*i*_ is the *ith* value (e.g. number of prescriptions with ≥ 11 claims), with ordering such that *x*_*i*_≤*x*_*i*+1_. *G* normally varies {0,1}. When *G*=0, all providers would have the same number of medication claims, while the closer we get to *G*=1, the more skewed the distribution.

## Results

### Volume and diversity of medicare prescriptions

**Fig. 1:**
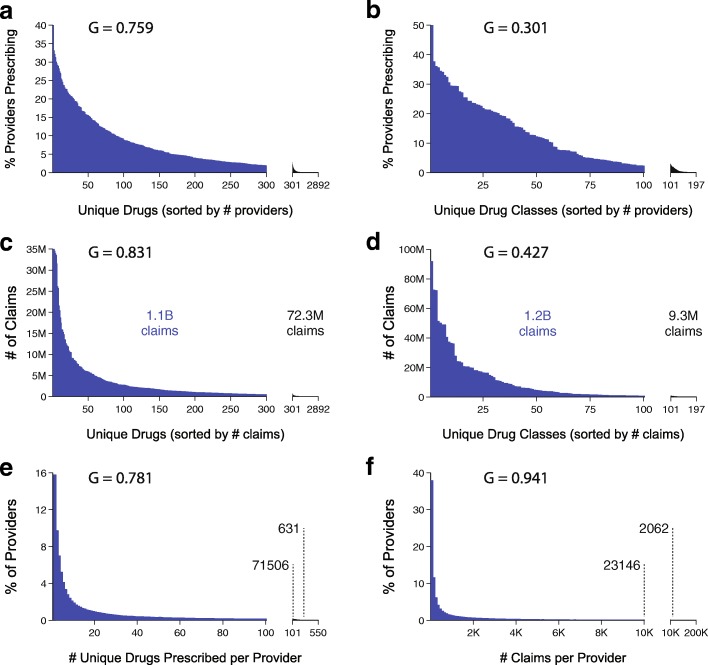
Overall features of 2013 Medicare Part D prescribing patterns data set. **a**. Distribution of percentage of providers prescribing each of 2892 unique drugs, sorted by percentage of providers prescribing. **b**. Same as A except for 197 unique drug classes. **c**. Distribution of number of claims for each of 2892 unique drugs, sorted by number of claims. Note that the unique drug order is not necessarily the same as in **a**. **d**. Same as **b** except for 197 unique drug classes. **e**. Distribution of drug prescription diversity across all providers sorted by number of unique claims. Numbers of providers prescribing more than 100 and 300 unique drugs are annotated on plot. **f**. Distribution of number of claims across all providers sorted by claims per provider. Number of providers making more than 10,000 and 25,000 claims are annotated on plot. *G* = Gini index

As a prelude to dimension reduction and visualization, we first examined the overall univariate statistical distributions of prescribing volume and diversity among medication classes and providers (Fig. 1). This step allowed us to assess the utility of dimension reduction visualization methods, which would be best suited to data with high variation and skewed distributions of medication volumes and prescribing diversity.

We found that small fraction of the unique Medicare Part D outpatient medications were prescribed by > 5% of providers (Figs. 1a and 1c). Only 165 unique drugs (5.7%) were prescribed by ≥ 5% of providers (Fig. 1a). Similarly, only 197 unique drugs (6.8%) had more than one million claims across all providers (Fig. 1c). To reduce the effect of formulary and brand name versus generic medication restrictions, we mapped unique drugs onto 197 categories (Fig. 1b and 1d). Distribution skewness was assessed by the Gini index (G), which has the property of *GI*=0 if all providers prescribed the same number of medications or all drug types were prescribed at the same volume, and approaches *G*=1 with increasing skew of the distribution [53]. Drug class distributions were less skewed; for all drugs *G*=0.759 versus for classes *G*=0.301, with 72 drug classes (36.5%) prescribed by ≥ 5% of the providers, and 83 classes (42.1%) surpassing one million claims across all providers.

We next examined provider prescription diversity, defined as the number of different drugs prescribed by each provider (Fig. 1e). The majority (70.3%) of providers prescribe ≤ 25 unique drugs reimbursed by Medicare (Fig. 1f), with 71,506 providers prescribing ≥ 100, and 631 providers ≥ 300 unique drugs. We hypothesized that high volume prescribers were more likely to be general practitioners (i.e. general medicine, internal medicine, family medicine). There were 2062 high-volume prescribing providers (HV) with ≥ 25,000 claims, utilizing 1954 of the 2892 available drugs. This group of 0.2% of providers were responsible for 3.59% Medicare Part D drug costs in 2013. Compared with the standard volume prescribing providers (SV; *n*=805,958), this small subset of HV (*n*=2062) was heavily skewed towards general practice (*p*< 0.001): 89% of HV providers were categorized as either internal medicine, family medicine or general practice (SV = 25.8%), and 3% were geriatric medicine (SV = 0.2%).

We further examined the differences in the patient populations cared for between, and Medicare costs, between the low volume (≥ 1000 prescriptions) and high volume (≥ 25,000 prescriptions) prescribers (Additional file 2: Table S2). High volume prescribers had higher numbers of unique beneficiaries and Medicare payments per provider (*p*< 0.0001). They also had higher percentages of beneficiaries Hispanic and Asian Pacific Islander patients (*p*< 0.0001), with both Medicare and Medicaid entitlement reimbursement (*p*< 0.0001), with dementia (*p*< 0.0001), chronic kidney disease (*p*< 0.0001), diabetes (*p*< 0.0001), heart failure (*p*< 0.0001), ischemic heart disease (*p*< 0.0001) and rheumatoid arthritis (*p*< 0.0001). Thus, high volume providers appeared to have Medicare patient panels skewed towards chronic conditions, many of which require multiple medications for ongoing treatment.

### Regional prescribing volumes and drug diversity

**Fig. 2:**
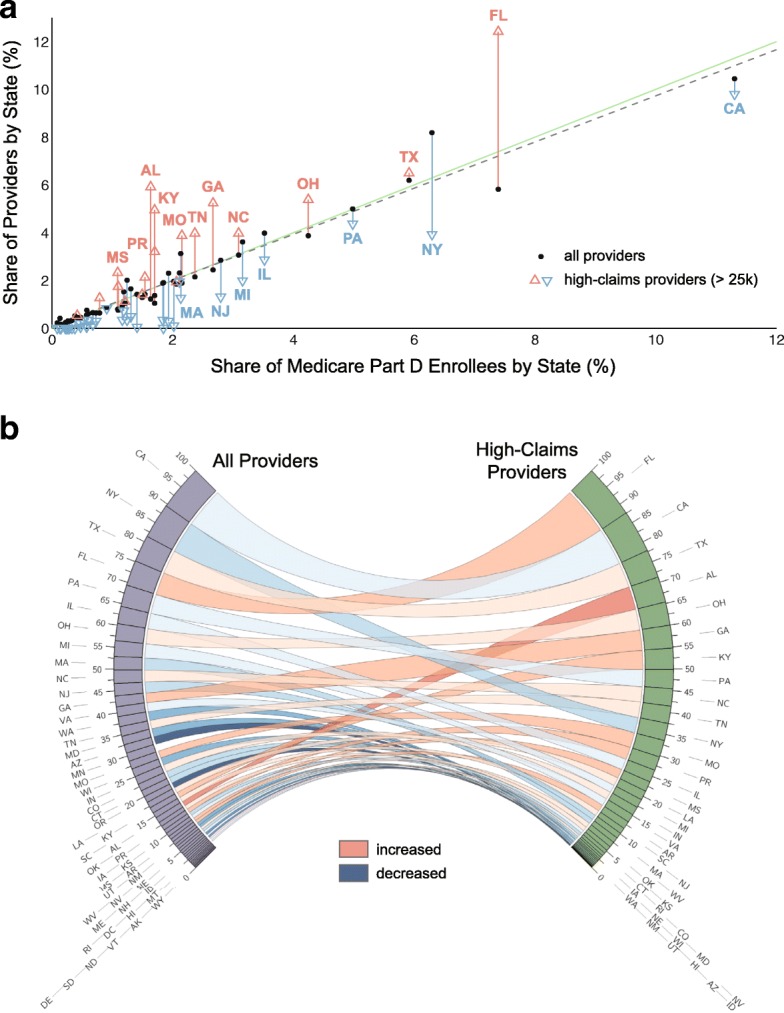
Distribution of Medicare Part D providers across states. **a**. Share of providers by state (as a percentage of the total number of providers) plotted against share of Medicare Part D enrollees by state (as a percentage of the total number of enrollees nationwide) are shown by black circles and fit to a line (gray dashed line); green line is slope of one. A similar plot based on a data subset of high-claims providers (> 25,000 claims resulting in 2062 providers) is shown superimposed as open triangles colored by their relation to the corresponding data from the full data set. Some states are annotated. **b**. Comparison of the provider composition by state for the full data set (left) and the high-claims data set (right). Ribbons connecting the two join corresponding states

Prescribing volumes may be related to population density, and thus examined the degree to which they correlated with the regional distribution of Medicare Part D prescription benefit enrollees. We therefore examined the relationship between prescribing volumes (overall versus HV providers), density of Medicare Part D enrollees, and prescription volumes. The number of Medicare Part D providers in each state was highly correlated with the corresponding number of Medicare Part D enrollees (Fig. 2a, *R*^2^=0.950), but not (*R*^2^=0.697) for providers with > 25,000 claims. There were substantial deviations for several states. For Florida and New York, these deviations may be due to differences in the ratios of providers to enrollees, such that Medicare drug prescribing was more/less concentrated among those providers. In contrast, several states with a proportional number of providers and enrollees had more high-claims providers (e.g. Georgia).

Figure 2b compares the ranking of all providers versus high-claims providers, with ribbons joining corresponding states. In contrast to the relatively similar ratios of Medicare providers per enrollee across states, the distribution of high-prescribing providers varies regionally (Additional file 3: Table S1). In general, high volume providers also had high prescribing diversity (Additional file 4: Figure S3). This distribution can be used to identify outliers in terms of prescribing diversity and volume. For example, only 10 Medicare providers accounted for approximately 12% of all 2013 Medicare Part D zoster vaccine claims, each with ≥ 10,000 claims accounting for over $30 million in claims. Such univariate outlier analyses are increasingly used to screen for activity defined as inappropriate or fraudulant (e.g. excessive opioid prescribing, prescription fraud). In this case, the data did not contain sufficient information to discriminate between potential explanations (e.g. fraud, contractual agreements with outpatient pharmacy chains, medical directorship of a large nursing home or eldercare facility).

### High dimensional provider prescribing patterns highly correlate with provider specialty

While univariate prescribing volumes and diversity measurements are useful for describing aggregate patterns, they do not provide information about how closely related entire prescribing patterns are between individual providers. Specifically, we were most interested in how well PCA visualization performed against t-SNE with respect to visual clarity and the ability to visualize different clusters of providers by prescribing pattern and specialty. PCA uses orthogonal transformation to map a data set of potentially correlated variables into a new set of linearly uncorrelated variables (principal components). It is often used to visualize the relationship between high dimensional data elements and highlight the axes of greatest variation. In contrast, t-SNE maps data onto a non-linear projection designed to highlight differences between high dimensional feature distributions. t-SNE has an advantage over PCA for visualizing prescribing data because the embedding is not biased by a the skewed distribution of a few features, and t-SNE can reveal more subtleties in the differences between provider groups [13]. Thus, we hypothesized that t-SNE would allow greater visualization and discrimination between clusters of providers with different prescribing patterns.

**Fig. 3:**
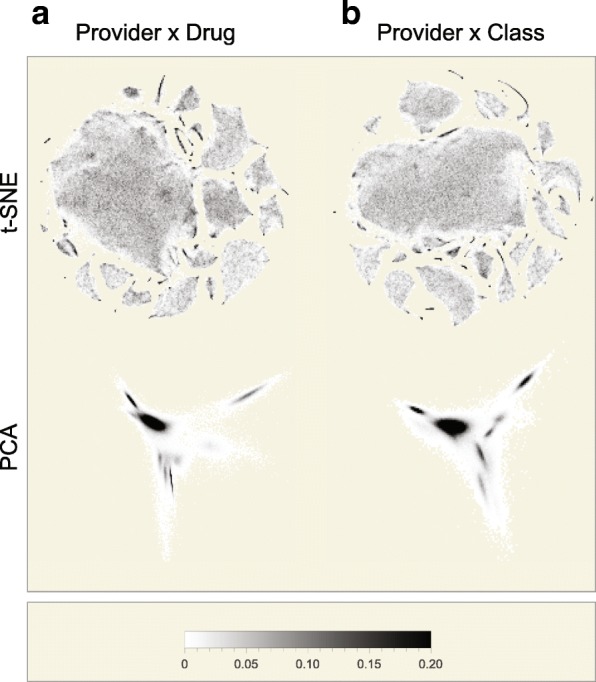
Low-dimension embedding of providers using t-SNE and PCA. 2-D density plots in low dimensional space created using t-SNE (upper) or PCA (lower) of 227,573 Medicare Part D providers, each with ≥ 1000 prescription claims in 2013 organized by **a** the 227,573×2791 drug claims matrix or **b** the 227,573×195 drug class claims matrix. Number-of-claims data per provider by drug or drug class is scaled by the total claims per provider to express the prescribing pattern as a composition prior to t-SNE

Figure 3 shows the projection of provider densities resulting from t-SNE and PCA applied to providers with ≥ 1000 claims (*n*=227,573) and using a feature vector of corresponding drugs (*n*=2791; Fig. 3a) or drug classes (*n*=195; Fig. 3b), where claim volumes in *Ω*_*i*_ were initially normalized by total claims per provider. Note the areas of very high density within the PCA projection obscuring finer variations in prescribing patterns. In contrast, t-SNE projections contain numerous spatially resolved groupings with fine detail visible, as well as one dominant grouping of Internal, Family, Geriatric, and General Medicine providers with areas of higher density reflecting subgroupings of providers with similar prescribing patterns.

**Fig. 4:**
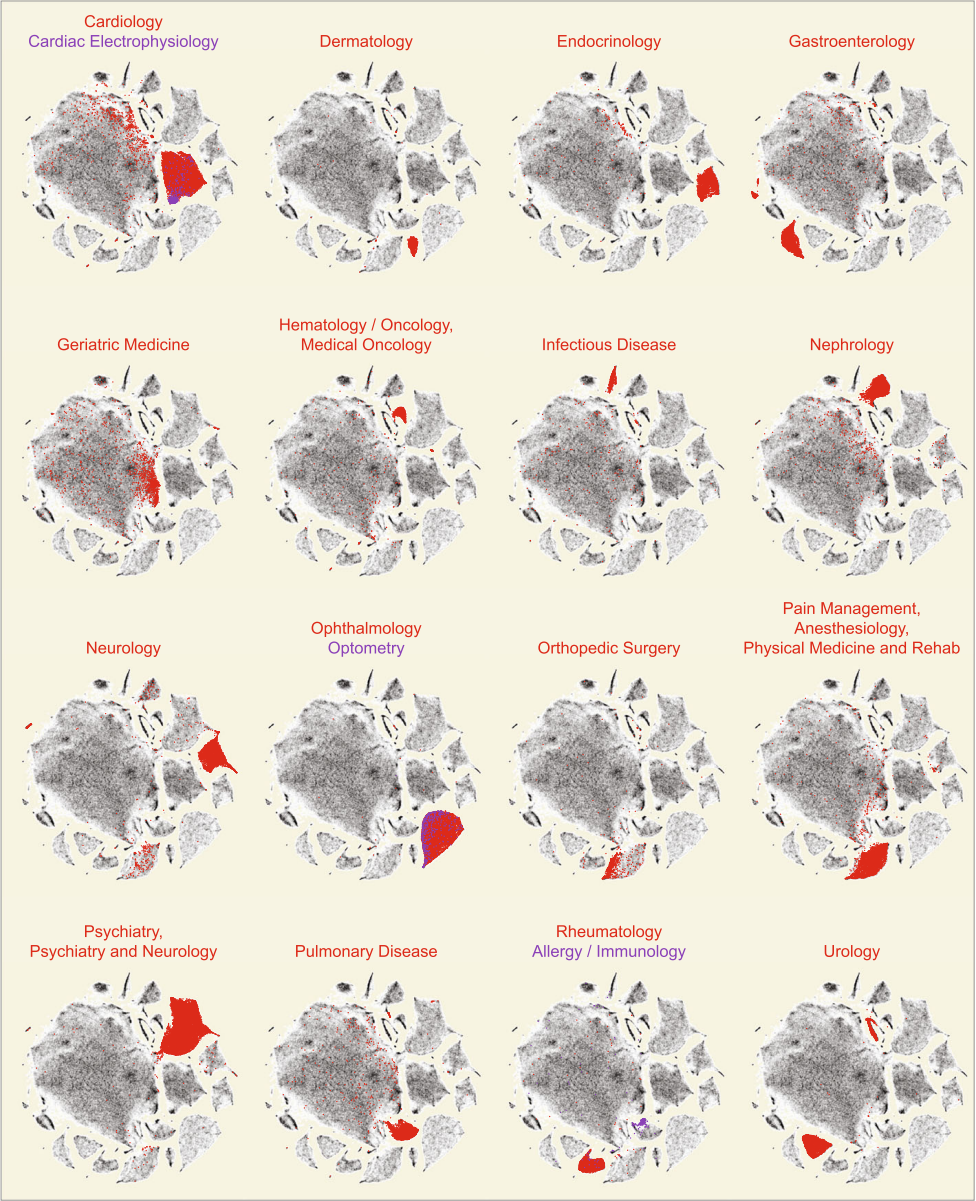
Array of t-SNE plots each highlighting providers of a specific specialty. Each 2-D density plot (grey) is the same as shown in Additional file 5: Figure S4A, and represents the set of 227,573 Medicare Part D providers ×2791 drug claims. Included providers had ≥ 1000 prescription claims in 2013. The plot is a heatmap, with densities representing increased numbers of providers. Provider specialties are shown in red to emphasize their collocation by prescribing pattern, and are labeled by NPPES self-reported specialty designation. Note the separation of provider clusters, even to the extent that subspecialties (annotated in blue) are distinguishable within the specialty cluster (e.g. Cardiology and Cardiac Electrophysiology

The t-SNE groupings are highly correlated with provider specialty and subspecialty (Fig. 4). These plots, based on the provider-by-drug matrix and cross-referenced with provider specialty from the National Plan and Provider Enumeration System (NPPES) database, highlight that some specialties have single dominant clusters (e.g. Dermatology, Endocrinology, Nephrology) whereas others have multiple clusters or sub-clusters that reflect groupings of sub-specialty practice within a specialty (e.g. Gastroenterology, Urology). Furthermore, when compared to PCA, t-SNE clearly provides better visual resolution of related medical specialties and sub-specialties within the projection (e.g. Cardiology and Cardiac Electrophysiology).

### Visualizing details of provider prescribing patterns

**Fig. 5:**
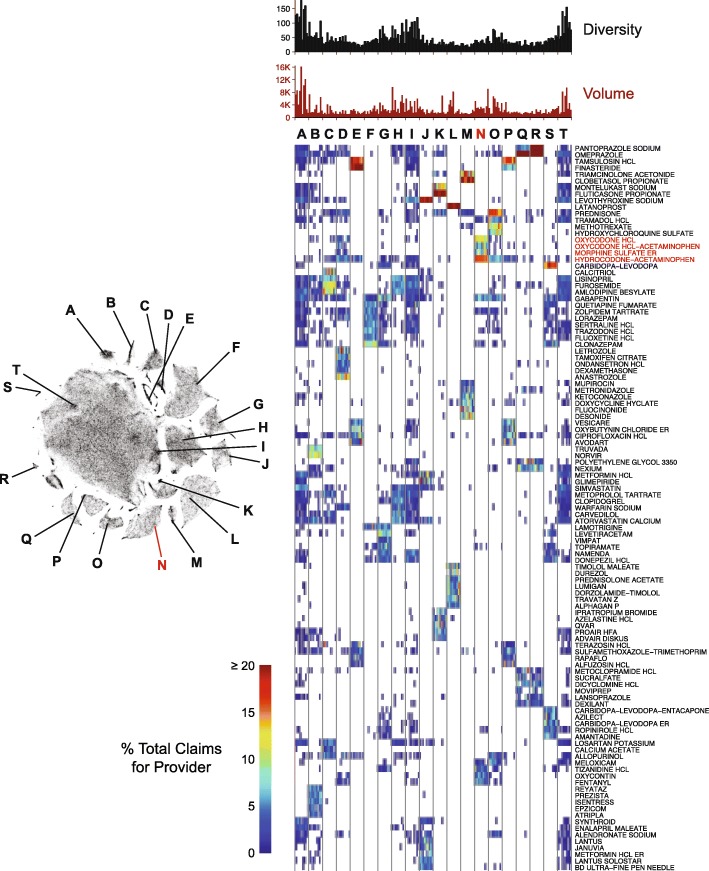
Representative prescribing patterns corresponding to different regions of t-SNE plot. Left: t-SNE plot as shown in Additional file 5: Figure S4A with 20 different regions labeled as A through T. Right: Heat map showing prescribing patterns. Columns are individual providers, 10 randomly selected from each of the 20 regions. Each row represents a drug. The drugs shown are the union of the top eight most frequently prescribed in each region. Increasing gray density corresponds to the percent of claims for a particular drug made by a provider relative to their total claims, with white denoting no claims. Prescribing volume (total claims) and diversity (number of unique drugs prescribed) are shown above the heat map as bar graphs. Note region N, which is enriched for providers with a high volume of opioid analgesic claims

We next used t-SNE to visualize prescribing diversity across many different provider cluster regions (Fig. 5) using the full provider-drug matrix. Ten random providers were chosen from 20 regions of the low-dimensional t-SNE visualization (Fig. 5, labeled A-T), which mapped to 47 different agglomerative clusters. Location within the embedding clearly maps different prescribing patterns. For example, regions E and P both are dominated by Urology (see Fig. 4), but E is characterized by large proportions of claims for tamsulosin and finasteride, whereas P is mainly tamsulosin. Cluster L is largely Ophthalmologists, consistent with high proportions of latanoprost and to a lesser extent, timolol maleate, Lumigan (bimatoprost), Alphagan(brimonidine tartrate) and similar drugs. Area K is enriched for Allergists that prescribe high proportions of fluticasone proprionate and montelukast sodium. Cluster N is enriched for providers with a high incidence of opioid analgesic prescriptions.

The t-SNE visualizations allow visualization of prescribing patterns likely associated with treating different patient populations, even within the same specialty. For example, groups G and S are dominated by Neurologists, but with substantially different prescribing patterns. Providers in cluster S prescribe large amounts of Parkinson’s disease medications (i.e. carbidopa-levodopa, ropinirole, amantidine, azilect), whereas those in cluster G are biased towards medications used to treat epilepsy and Alzheimer’s disease (i.e. levetiracetam, lamotrigine, lacosamide, topiramate, namenda and donepezil). In other cases, regional variation may strongly influence prescribing patterns. For example, cluster A is dominated by providers from Puerto Rico. These results demonstrate the utility of using t-SNE to visualize variation of prescribing patterns that highly correlate with formal provider clusters.

### Visualizing prescribing volume and medication distribution patterns

**Fig. 6:**
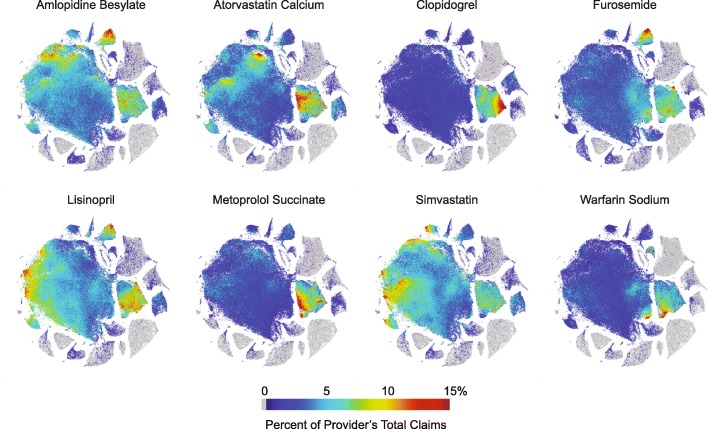
Array of t-SNE plots of providers annotated for fraction of claims for each of eight heart/circulation related drugs. The t-SNE plots were created from the set of 227,573 Medicare Part D providers ×2791 drug claims. Included providers had ≥ 1000 prescription claims in 2013. The color for each provider corresponds to the percentage of claims for the indicated drug relative to the provider’s total claims. Gray is 0%, the maximum scale (red) is 15% of total claims. Note the high volume of prescriptions within within both the cardiology and internal medicine areas

t-SNE plots can also be annotated by the prescribing proportions for individual drugs (Fig. 6). Here, for eight drugs typically prescribed for cardiovascular-related conditions, the percentage of claims for individual providers relative to their total number of claims are coded by color. Note that these are visible as high proportions within the region corresponding to Cardiology (see Fig. 4). Even within the Cardiology region, high prescription rates of these drugs are associated with different provider groupings (see for example, atorvastatin, clopidogrel, and warfarin). These groupings may reflect differences in provider scope of practice, patient populations, Medicare formularies, or provider prescribing preferences.

In a similar fashion, the dimension-reduced space can be annotated by claim volume as shown in Additional file 5: Figure S4. In this figure, each point is color coded by claim volume. There is slight gradient of claim volume in the large, central General Medicine/Internal Medicine/Family Practice region with several small densities of extremely high prescribing volume providers (e.g. ≥ 10,000 claims). Claim volume also correlated with drug diversity (see Additional file 4: Figure S3), so volume will be somewhat conflated with prescribing pattern and will affect position in the low-dimensional embedding. However, plots highlighting single drugs suggest that the variation across the large t-SNE region correlate well with the prescribing patterns of individual providers (Fig. 6). Finally, it is important to recognize that such visualizations allow comparison of high-dimensional co-prescribing variation across thousands of individual provider patterns, in contrast to bar graphs showing the top 10 medications proportionally prescribed within a self-identified specialty class (see Additional file 6: Figure S6).

**Fig. 7:**
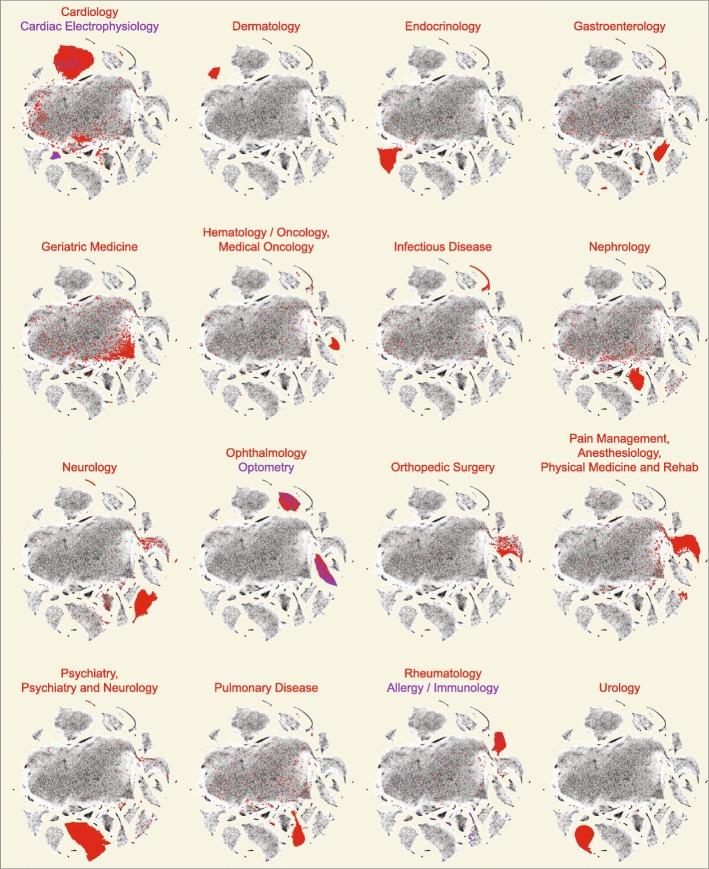
Array of t-SNE plots each highlighting providers of a specific specialty. These t-SNE plots are derived from the dataset of 227,573 Medicare Part D providers ×195 drug classes. Even with dimension reduction from 2791 individual medications to 195 medication classes, t-SNE plots produced clear groupings of specialties and subspecialties. This plot removes potential bias introduced by prescribing of generic versus brand name medications, and thus is a better representation of prescribing variation across specialties due to patient populations and practice patterns

**Fig. 8:**
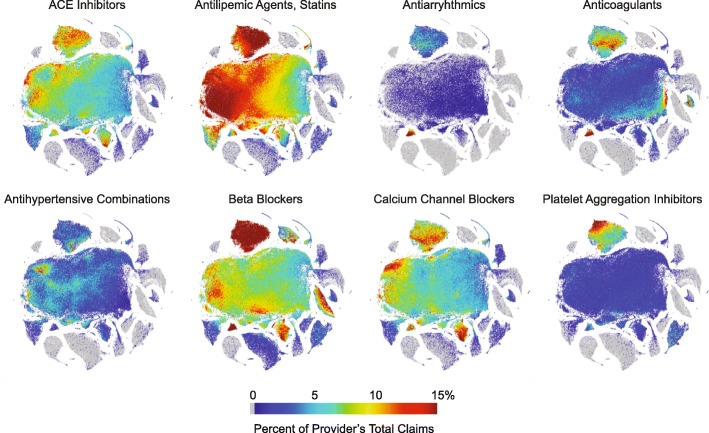
Array of t-SNE plots of providers annotated for fractions of claims for each of six cardiac drug classes The t-SNE plot layout was generated using the dataset of 227,573 Medicare Part D providers ×195 drug classes. The 195 drug classes include all medications (generic and brand name) collapsed into the the indicated class. The color for each provider corresponds to the percentage of claims for the indicated drug relative to the provider’s total claims. Gray is 0%, the maximum scale (red) is 15%. This dimension reduction and visual representation eliminates differences due to formulary, or generic versus brand name medication prescribing patterns. Note, for example, the high percentage (red areas) of beta blockers prescribed in cardiology and nephrology (oral preparations) and opthomology (eye drops)

Figure 7 shows the specialist-annotated embeddings based on medication class (see Fig. 3b). As with the embeddings based on individual medications, specialists are enriched in the smaller clusters surrounding the main cluster. Figure 8 shows this embedding annotated for prescription proportion of six cardiology-related drug *classes* (similar to Fig. 6). Even when considering classes instead of individual drugs, which eliminates clustering differences due to separately considering different formulations of the same drug (i.e. generic and brand name), there are clearly large variations in prescription patterns within the cardiology cluster (see for example, anticoagulants, calcium channel blockers, and platelet aggregation inhibitors).

### Hierarchical clustering of provider prescribing patters

**Fig. 9:**
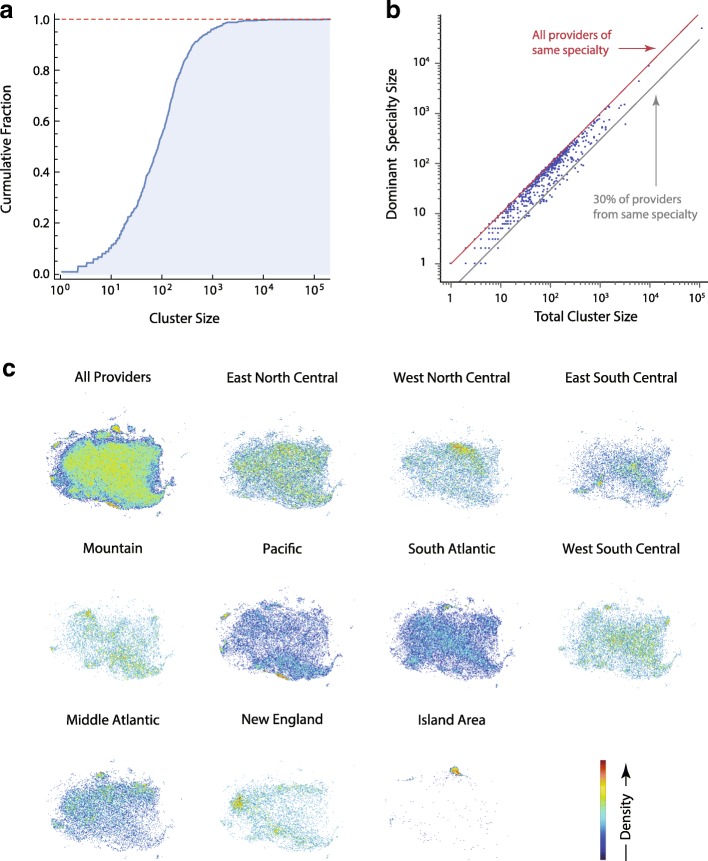
Unsupervised hierarchical clustering by drug class. Provider clusters obtained by hierarchical agglomerative clustering using a Euclidean distance measure and centroid criteria. **a**) Cumulative distribution of provider size over 605 clusters. **b**) Provider specialties within each cluster were tallied and the number of providers in the dominant specialty plotted against cluster size. The lines indicates where 100% (red), or 30% (gray) of providers in the cluster are the same medical specialty. **c**) t-SNE visualization of provider prescribing pattern variation for Family Medicine providers by United States Federal Region. Each plot represents a 2D density histogram

To more rigorously identify provider subspecialty association within t-SNE heatmap regions, we performed unsupervised hierarchical cluster analysis. We identified 605 provider clusters using agglomerative clustering with Ward’s minimum intercluster variance linkage minimization (Additional file 7: Figure S5, and Fig. 9a). The dominant provider subspecialty classification within a cluster, taken from the NPPES data, was used to map each of the 605 sub-clusters to provider sub-specialties. Ninety one percent of the clusters had one provider specialty accounting for ≥ 30% of the providers (Fig. 9b). Of those clusters with ≥ 2 specialties (*n*=595), 34.5% of the second most frequent specialties were either nurse practitioner or physician assistant, roles rather than disease-based identification. Inclusion within these clusters suggested practice scope within the dominant specialty. When mapped to US Federal Regions (Fig. 9c), clusters also reflected regional variation in prescribing patterns. For example, within the t-SNE projection, we highlighted sub-clusters of providers identified as Family Medicine and then divided by Federal Region. This combination of clustering and t-SNE visualization made visible large regional variations in regional medication prescribing volumes and patterns within Family Practice.

### Regional variation in prescribing patterns

Given the variation in regional prescribing patterns observed within the Internal Medicine-Family Practice-General Medicine cluster, we hypothesized that such variation was present across all Medicare Part D program providers. To test this hypothesis, we next performed an in-depth characterization of regional differences in prescribing patterns over all sub-specialties by census region (Additional file 8: Figure S2).

**Fig. 10:**
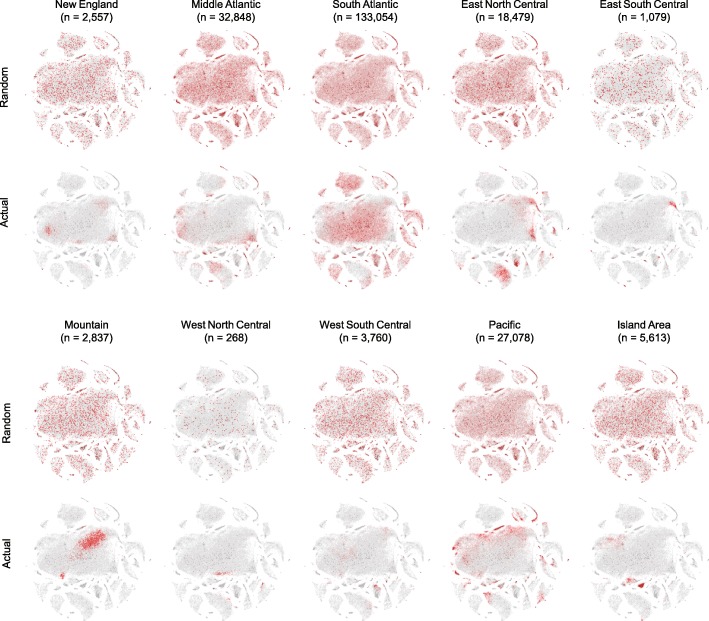
Distribution of provider prescribing patterns by census region. Providers with ≥ 1000 claims (*n*=227,573) were divided into subsets by census region (lower figures within regional pairs). For comparison, a random sample of equivalent size was taken from the entire data set such that the providers in each random sub-sample did not overlap with any of the others (upper figures). This allows visual comparison of regional provider distributions with a random national sample of equivalent size

Figure 10 shows how the prescribing patterns of providers with ≥ 1000 Medicare Part D claims are clustered within each census region, as compared to a non-overlapping random sample from the entire data set. For these visualizations, we used heat maps of provider density within the t-SNE embedding. This type of visualization accounts for equivalent sample sizes, but not variation in the proportion of Medicare Part D provider types (e.g. Family Practice versus Nephrology) between the random and regional samples. For example, the East North Central region has a much higher percentage of Neurologists compared with the East South Central region. Differences in provider and population density, and thus prescribing patterns and volumes, may also contribute to regional variations in Medicare part D prescription costs. The utility of the t-SNE visualization can be seen by comparison with traditional univariate bar graphs 6, which only shows differences in the univariate prescribing percentages for single medications, and provides no information about variation of co-prescribing patterns among individual providers.

### Urban prescribing pattern variation

The results from dimensional reduction visualization with t-SNE were again hypothesis generating, and suggested that regional prescribing patterns could be due to urban location, variation in income, or population density. To further explore regional variations in prescribing patterns, while diminishing the impact of these variables, we selected 52 metropolitan areas (core-based statistical areas, CBSA) with populations greater than one million (Additional file 9: Figure S7). Among the large metropolitan areas, there were large regional differences in terms of proportion of Medicare Part D enrollees of the total population, as shown in Additional file 9: Figure S7, ranging from 4.6% (Washington DC) to just under 15.7% (Pittsburgh). These results were not statistically correlated to overall population of the respective CBSAs.

**Fig. 11:**
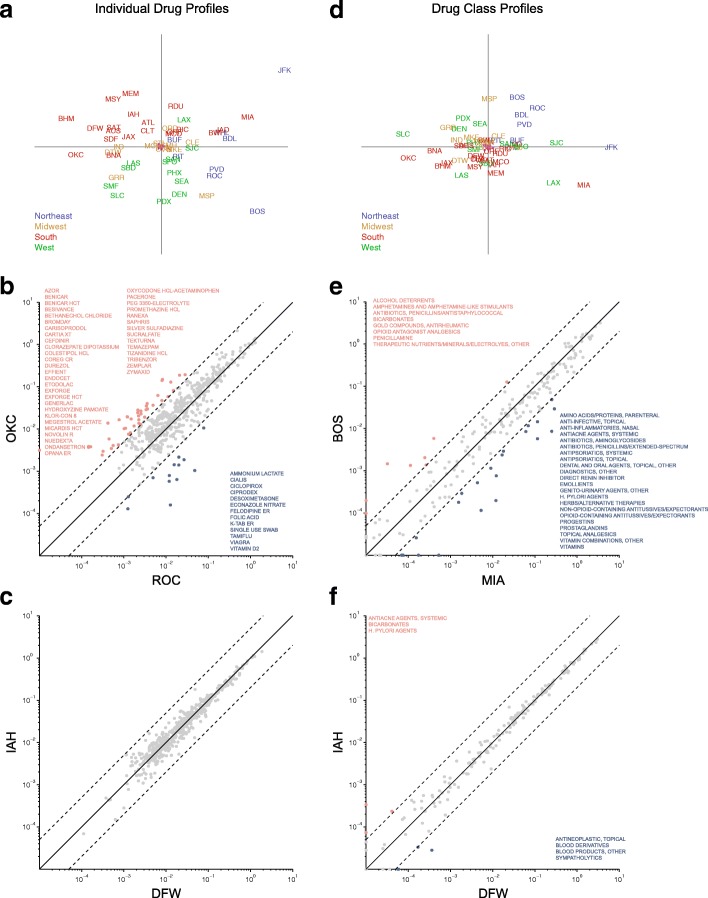
Variation of prescribing pattern by core-based statistical areas. **a**. Multidimensional scaling (MDS) of 52 CBSAs based on 532 drugs that have over 100,000 claims (across 50 states and Washington DC). Data were expressed as number of claims for a particular drug in a particular CBSA per number of enrollees in that CBSA. CBSAs are specified by IATA airport code. Magenta dots indicate MDS performed on a randomly permuted data sets where the data corresponding to the CBSA providers were shuffled, preserving the number of providers for each CBSA. **b**. Comparison of two CBSAs of similar sizes: Oklahoma City OK vs. Rochester NY. Dots represent individual drugs and axes are the number of claims per enrollee in log scale (for the respective CBSAs). Dashed lines indicate 5-fold differences in the per-enrollee numbers of claims. Drugs beyond these regions are indicated. **c**. Comparison of Houston TX and Dallas-Fort Worth Texas CBSAs that might be expected to have similar profiles as an internal control. **d**. MDS plot of 52 CBSAs based on 198 drug categories, similar to part A. **e**. Comparison of prescribing patterns in Boston MA and Miami FL based on drug categories. **f**. Houston TX vs. Dallas-Fort Worth TX based on drug categories

Dimension-reduction with t-SNE visualizations also revealed *regional* variation in prescribing patterns across CBSAs. To characterize prescribing profiles within CBSAs, we selected 532 drugs with over 100,000 claims for all states. A 52 CBSA by 532 drug number-of-claims matrix was computed and each row was divided by the number of Medicare Part D enrollees in the corresponding CBSA, expressing the normalized data as drug claims per enrollee. Figure 11a shows the first two coordinates of the resulting multidimensional scaling based on pairwise CBSA-CBSA distances *d*_*i*,*j*_=1−*r*_*i*,*j*_, where *r*_*i*,*j*_ is the Pearson product-moment correlation coefficient for the CBSA pair *i* and *j* feature vectors. The red dots near the center of the plot are the result of multi-dimensional scaling following random permutation of the CBSA provider memberships (preserving the relative numbers of providers per CBSA) used as a reference against which to interpret the dispersion of the real data. Although the data do not segregate into distinct clusters in this dimension, there are apparent regional variations, notably, that most of the southern CBSAs appear on the left half of the plot, reflecting similar regional prescribing profiles within the southern CBSAs.

Further visualizations highlight the substantial variation in provider prescribing patterns between CBSA’s. Figure 11b shows an example of claims-per-enrollee of the 532 drugs for two geographically distant but similarly sized CBSAs: Rochester, NY (ROC) and Oklahoma City, OK (OKC). Although their populations are similar, they have different median household incomes and percent Medicare Part D enrollees (see Additional file 5: Figure S4): $43,955 and 14.1%, respectively for ROC, and $36,797 and 7.8% for OKC. The dashed lines represent 5-fold differences in claims-per-enrollee for specific drugs, with those outside the range annotated. The selected CBSAs are annotated in t-SNE density plots shown in Additional file 10: Figure S8A. For comparison, Fig. 10c shows another pairwise visual comparison between two geographically proximate and similarly sized CBSAs: Dallas-Fort Worth, TX (DFW) and Houston, TX (IAH). If prescribing patterns reflect regional prescribing homophily or state-specific Medicare Part D approved medication formularies, such pairs would be expected to have similar prescribing profiles and could be considered an internal control. In this example, the claims per enrollee are more similar between the two CBSAs. The median household incomes and percent enrolled are $47,418 and 6.6% for Dallas Fort Worth (DFW), and $44,714 and 6.3% for Houston (IAH). These results provide further support for the hypothesis that regional variation in prescribing patterns increases with geographic distance.

### t-SNE identifies regional variation in prescribing patterns

Medicare formulary composition varies by state and region. Such variation may lead to prescribing pattern differences between providers based on drug formulations, rather than the use of similar drugs of the same class. To control for this effect, we next examined the results obtained by dimension reduction and visualization with t-SNE based on drug classes, rather than individual medications. Figures 11d-f show results based on profiles of 195 drug categories, which still show substantial differences prescribing profiles between CBSAs. Figure 11e compares the Boston, MA (BOS) and Miami, FL (MIA) CBSAs (also see t-SNE plots in Additional file 10: Figure S8B), with 5- to 10-fold differences the claims-per-enrollee for some categories. While these are sized metropolitan areas, there are almost twice as many enrollees per provider in MIA than in BOS (see Additional file 9: Figure S7 and Fig. 2). As an example, “Amphetamines and Amphetamine-Like Stimulants” generate almost 6-fold more claims per 1000 Boston enrollees as compared to claims per 1000 Miami enrollees (126.4 vs. 21.7). In contrast, “Genito-Urinary Agents, Other” generate almost 10-fold more claims per 1000 enrollees in MIA as compared to BOS (28.9 vs. 2.9). Figure 11f shows that the Dallas-Fort Worth vs. Houston profiles are substantially more similar, with the largest differences for rarely prescribed drug categories.

One possible cause for regional variation in prescribing patterns could be differences in disease prevalence between regions. We used Medicare data on the disease prevalence for 13 conditions (see Additional file 11: Figure S9 for a detailed list and explanation) to construct a feature vector *D*={*δ*_1_,*δ*_2_,...*δ*_*n*_}, where Medicare providers with ≥ 1000 Medicare prescriptions in 2013 (*n*=207,158) and complete data were grouped by state (50 US states, the District of Columbia, and Puerto Rico). We then calculated the mean feature vector prescribing pattern and provider patient-specific disease prevalence values for each state’s providers. To test whether the multi-dimensional drug prescribing pattern differences were correlated with multi-dimensional disease prevalence, we calculated the Euclidean n-dimensional matrix of distances between each pair of states for both prescribing pattern distances and disease prevalence distances. Thus, that states with similar Medicare prescribing patterns should have have small multi-dimensional Euclidean feature distances, while those that differ would have large feature distances. A similar relationship would exist for n-dimensional feature distances calculated using the disease prevalence feature vector; pairs of states with similar prevalence of diseases would have small n-dimensional Euclidean feature distances. We found the correlation between disease prevalence and prescribing pattern distances to be *R*^2^=0.22185, indicating that variation in multi-dimensional prescribing patterns between states cannot be explained simply by variance in multi-dimensional disease prevalence.

## Discussion

Our results demonstrate that t-SNE dimensional reduction can be used to visualize prescribing pattern variation in very large administrative data sets, and reveal patterns not otherwiseapparent.

Previously, a number of focused studies have examined prescription diversity, mostly with respect to opioid analgesics [56–62], antibiotics [1, 63–67], psychiatric medications [68–71], and among general practitioners [37, 72–76]. One web site has made the Medicare Part D prescribing data searchable with various filters for provider, charges, and medications [77–79]. As far as we are aware, however, this is the first high level, aggregate analysis of provider prescribing diversity and patterns on a national scale (40 million patients and over 800,000 providers) across multiple specialties, medication classes and practitioner types. This type of analysis may be used as a starting point for future work comparing national prescribing patterns, especially in countries where regional formulary composition is centrally tracked. Thus, this multivariate approach has value in establishing an atlas of prescription *pattern* diversity, and can be a means for deeper, more targeted queries about groupings or sub-groupings of providers.

Provider prescribing volume and diversity patterns could be a powerful proxy for organizing how practitioners actually provide care, as opposed to self- or board- identified medical specialty. For example, providers with a “mixed practice” (e.g. adult internal medicine and endocrinology) will have prescribing patterns that differ from those practicing solely within one specialty. There are currently no data sets, survey results or accepted methods to identify such mixed-practice providers. Thus, our results are hypothesis generating and suggest that such practice mixes can be identified by unsupervised clustering of prescribing patterns, and visualized with t-SNE. Further work will need to be done to test this hypothesis, and could involve comparing survey data about self-identified practice mix with prescribing patterns. The current study provides the motivating hypothesis and groundwork for such investigations.

Additionally, our approach enhances hypothesis generation and testing regarding root causes of prescribing variation. For example, correlating provider clusters with clinical outcomes data may improve comparative effectiveness studies of prescribing patterns for specific diagnoses (e.g. effect of anti-hypertensive regimens with and without diuretics on blood pressure control and mortality) [80]. Similar approaches have recently been used to conduct “virtual clinical trials”, replicating the results of randomized prospective clinical trials [81, 82], but lack a visualization component. Our results demonstrate that these methods can be used to identify and visualize complex, multi-dimensional, prescribing behaviors of interest (e.g. opioid prescribing) in geographically comprehensive data sets. In the future, studies coupling prescribing patterns, patient outcomes, and genomic data may aid in identification of genotype-phenotype associations and facilitate precision targeting of effective therapies to specific individual genotypes [83].

Our analysis and t-SNE visualizations also highlight prescribing variation in groups of metropolitan providers with similar Medicare claims patterns. These findings complement reports showing considerable geographic variation in both claims volume [84] and cost [4] across the United States. Potential contributing factors to such variation [35, 85–87], include suboptimal care or health services delivery inefficiencies [88, 89], and regional differences in prescriptions for branded drugs compared to generic counterparts [90–93]. The analysis of metropolitan areas, adjusted for population density, revealed considerable residual variation in prescribing patterns, with up to ten-fold variations for both individual drugs and drug classes.

Further work, incorporating more detailed data (e.g. regional Medicare formularies, provider-health system associations), are needed to determine the factors associated with such variation. Interestingly, we found that prescribing pattern differences increase with geographic differences. However, our results showed only modest correlation between n-dimensional prescribing patterns and n-dimensional disease prevalence among states. Regional prescribing patterns may be shaped by local factors (e.g. economic, social, state-specific Medicare formularies, local and regional provider practice patterns) Further work will need to be done to better elucidate sources of such regional variation. Nevertheless, these findings are a significant advance over single-specialty or disease-based variation studies, providing a method to compare comprehensive medication co-prescribing patterns.

Several caveats apply to this analysis. First, we recognize that most Medicare providers have a patient population with a mix of prescription plans, and our results may not be applicable beyond the Medicare population demographics [94]. For example, only 15.5% of Medicare Part D enrollees were ≤ 65 years of age. Thus, the prescribing profiles and provider cluster memberships described here cannot be generalized to younger individuals. Approximately 50% of individuals enrolled in Medicare Part D also have private or supplemental insurance for medication coverage, and prescription claims captured by Medicare Part D may differ from overall claims. This bias is somewhat mitigated by our selection of 227,000 providers with ≥ 1000 claims. Unfortunately, there is currently no available data set for the United States integrating the medication formularies of all the Medicare plans. Thus, we are unable to judge to what extent prescribing variation is dependent on Medicare Part D plan formulary differences. Future work might explore these issues with more comprehensive US data sets, or data sets from countries with national healthcare systems where formulary information is available.

## Conclusions

In conclusion, we have presented a pattern-based approach for visualizing prescribing variation in a national administrative data set. The analysis highlighted regional variations in prescribing practices in the United States Medicare Part D program and captured this diversity based on overall prescribing patterns as opposed to single medications. The use of the t-SNE visualization algorithm enhances the analysis and visualization of variation in high-dimensional co-prescribing data, and can be used as a hypothesis generating method.

## Additional files

Additional file 1 **Figure S1.** Data sources used for this study. This schema depicts various sources of data and how they are related. Red font indicates a data column with unique values. (EPS 332 kb)

Additional file 2 **Table S2.** Differences between providers by services, patient beneficiary demographics, and payments. Comparison between low volume (≤ 25,000 prescriptions over 12 months) and high volume (> 25,000 prescriptions over 12 months) provider patient populations. In general, high volume prescribers had a higher proportion of patients with more complex medical conditions (e.g. cancer, Alzheimer’s disease, heart failure), more elderly patients, and much higher use of Medicare services. (PDF 123 kb)

Additional file 3 **Table S1.** Differences in high-prescribing provider fractions by geographic region. Table quantifies the fraction of high prescribing Medicare prescribers by United States Administrative Region (see Additional file 1: Figure S1 for region definitions). (PDF 60.6 kb)

Additional file 4 **Figure S3.** Comparison of prescribing diversity and prescribing volume. Density/scatter plot indicating the number of unique drugs (top) drug classes (bottom) prescribed (diversity; y-axis), number of claims (volume; x-axis) and number of providers bin height coded as color. Bins that have a single provider are indicated by a blue dot. (EPS 1751 kb)

Additional file 5 **Figure S4.** t-SNE plot showing distribution of claim volume per provider. This t-SNE plot is based on the provider by drug matrix, as shown in Fig. 3a. Color corresponds to the *Log*_10_ of claims per provider (each represented by a dot). (PDF 899 kb)

Additional file 6 **Figure S6.** Unidimensional bar graphs of medication class prescribing frequency by region. Bar graphs of each of the top 10 medication classes prescribed (by percentage of individual prescriber prescriptions) for each of 24 medical specialty groupings, plotted for each of 10 Federal Regions. Note that drug class prescribing percentages are mean levels, and truncated at 21% to make the visualizations informative. (ZIP 5280 kb)

Additional file 7 **Figure S5.** Hierarchical clustering. Plots of the 605 clusters identified by hierarchical clustering with linkage using Ward’s minimization criteria. The background is the full t-SNE projection, while each cluster is in red. This 19 page figure is available for download from https://figshare.com/account/projects/24664/articles/5388157. (PDF 19 MB)

Additional file 8 **Figure S2.** United States Census Regions. Map of United States Census Regions used for geographic data comparisons. Adapted from the United States Census Bureau. (PDF 249 kb)

Additional file 9 **Figure S7.** Characteristics of core-based statistical areas (CBSA). 52 CBSAs are listed that have July 2012 population estimates greater than 1,000,000 residents. See Methods for data sources. (PDF 24.2 kb)

Additional file 10 **Figure S8.** t-SNE plots with particular CBSAs highlighted. A. t-SNE plot based on provider by drug matrix (as in Fig. 3a) with providers in Rochester and Oklahoma City annotated (see Fig. 10b). B. t-SNE plot based on drug class by provider matrix (as in 3b) with providers in Miami and Boston annotated (see Fig. 10e). (PDF 1010 kb)

Additional file 11 **Figure S9.** Comparison of between state mutidimensional distance matrices for prescribing pattern versus disease prevalence. Provider specific data for drug class prescribing patterns (*n*=68 drug classes) and provider-specific patient disease prevalence (*n*=13 diseases) were obtained from Medicare public use files. Disease prevalence figures included dementia, asthma, atrial fibrillation, cancer, depression, diabetes, chronic obstructive pulmonary disease, chronic kidney disease, heart failure, hyperlipidemia, hypertension, ischemic heart disease, and stroke. Medicare providers with ≥ 1000 Medicare prescriptions in 2013 (*n*=207,158) and complete data were grouped by state (50 US states, the District of Columbia, and Puerto Rico). We then calculated the mean feature vector prescribing pattern and provider patient-specific disease prevalence values for each state’s providers. To test whether the multi-dimensional drug prescribing pattern differences were correlated with multi-dimensional disease prevelence, we calculated the Euclidean n-dimensional matrix of distances between each pair of states for both prescribing pattern distances and disease prevalence distances. Thus, that states with similar Medicare prescribing patterns should have have small multi-dimensional Euclidean distances, while those that differ would have large distances. A similar relationship would exist for n-dimensional distances calculated using the disease prevalence feature vector; pairs of states with similar prevalence of diseases would have small n-dimensional Euclidean distances. We then tested the correlation between disease prevalence and prescribing pattern distances by analysis of variance, finding an *R*^2^=0.22185, indicating that variation in prescribing patterns between states cannot be explained simply by variance in disease prevalence. (PDF 1370 kb)

## Abbreviations

CMS: Center for medicare services
CBSA: Core-based statistical areas
DEA: Drug enforcement agency
FIPS: Federal information processing standards
G: Gini index
HV: High-volume prescribing providers
NPPES: National plan and provider enumeration system
NPI: National provider identifier
PCA: Principal components analysis
SV: Standard-volume prescribing providers
t-SNE: t-distributed stochastic neighbor embedding. United States abbreviations for states can be found in Additional file 3: Table S1. Abbreviations for the 52 core based statistical areas used in this analysis can be found in Additional file 9: Figure S7.
