# Supplementary material for: Visualizing nationwide variation in medicare Part D prescribing patterns
Source: BMC Med Inform Decis Mak. 2018 Nov 19;18:103. doi: 10.1186/s12911-018-0670-2 (PMC6245567; doi:10.1186/s12911-018-0670-2)
Supplement: Supplementary file 2 — Table S2. Differences between providers by services, patient beneficiary demographics, and payments. Comparison between low volume (≤ 25,000 prescriptions over 12 months) and high volume (> 25,000 prescriptions over 12 months) provider patient populations. In general, high volume prescribers had a higher proportion of patients with more complex medical conditions (e.g. cancer, Alzheimer’s disease, heart failure), more elderly patients, and much higher use of Medicare services. (PDF 123 kb) [file 12911_2018_670_MOESM2_ESM.pdf]

Table S2: **Differences between providers by services, patient beneficiary demographics, and payments**

	Low Volume (<25,000)			High Volume (≥25,000)			
**Characteristic**	**n[a]**	**Median**	**IQR[b]**	**n[a]**	**Median**	**IQR[b]**	**p[c]**
**Annual Number of:**							
Medical Services	204,985	1,693	2,918	2,194	5,316	6,431	<0.00001
Unique Beneficiaries	204,985	325	429	2,194	752	496	<0.00001
Drug Services	177,597	75	291	2,015	258	896	<0.00001
Unique Beneficiaries With Drug Services	177,597	36	97	2,015	138	258	<0.00001
**Total Annual Medicare Payments ($):**							
All	204,985	77,789	144,558	2,194	23,4616	238,426	<0.00001
Drug	177,597	849	3,411	2,015	2,307	6,738	<0.00001
**Percent (%) of Beneficiaries:**							
Age < 65	183,046	16.7	19.4	2,124	16.6	13.8	0.00621
Age 65 - 74	190,604	38.4	12.6	2,168	31.9	11.4	<0.00001
Age 75 - 84	178,072	30.0	8.8	2,152	29.3	7.0	<0.00001
Age > 84	166,378	14.8	10.0	2,114	19.14	14.7	<0.00001
Female	204,985	57.3	12.2	2,194	59.8	7.0	<0.00001
Male	204,985	42.7	12.2	2,194	40.2	7.1	<0.00001
Non-Hispanic White	161,814	85.2	22.9	1,858	83.8	26.0	0.18117
Black or African American	89,650	10.2	20.2	1,153	10.3	18.0	0.11820
Asian Pacific Islanders	39,983	2.6	10.6	444	2.7	13.2	0.03933
Hispanic	67,737	7.1	19.7	786	8.8	40.2	0.00011
American Indian/Alaska Native	62,669	0.0	0.0	545	0.0	0.0	0.38909
With Race Not Elsewhere Classified	27,818	1.3	3.0	298	1.8	5.26	0.00082
With Medicare Only Entitlement	186,853	76.0	29.7	2,123	54.7	33.8	<0.00001
With Medicare & Medicaid Entitlement	187,358	23.9	29.58	2,123	45.3	33.8	<0.00001
**Percent (%) of Beneficiaries With:**							
Alzheimer's Disease or Dementia	170,606	12	11	2,138	25	25	<0.00001
Asthma	162,438	8	5	2,099	8	4	<0.00001
Atrial Fibrillation	166,907	12	8	2,089	13	8	0.90077
Cancer	165,559	10	5	2,106	9	4	<0.00001
Chronic Kidney Disease	190,883	23	17	2,159	29	17	<0.00001
Chronic Obstructive Pulmonary Disease	183,337	17	12	2,136	25	13	<0.00001
Depression	195,181	24	15	2,164	31	20	<0.00001
Diabetes	199,304	34	15	2,181	42	14	<0.00001
Heart Failure	185,511	19	18	2,155	30	18	<0.00001
Hyperlipidemia	201,296	58	20	2,189	58	19	0.00519
Hypertension	202,943	72	13	2,192	75	1	<0.00001
Ischemic Heart Disease	196,066	36	20	2,179	45	15	<0.00001
Osteoporosis	157,941	9	5	2,095	11	8	<0.00001
Rheumatoid Arthritis / Osteoarthritis	198,481	38	13	2,182	46	17	<0.00001
Schizophrenia / Other Psychotic Disorders	127,592	6	7	1,996	9	11.5	<0.00001
Stroke	135,837	6	6	2,050	8	6	<0.00001
Average HCC Risk Score of Beneficiaries	204,985	1.288	0.5635	2,194	1.61615	0.6345	<0.00001

[a] Reflects number of providers without missing or censored data (for n¡11 beneficiaries, per Center for Medicare Services privacy guidelines)
[b] IQR = inter-quartile range
[c] Mann-Whitney U test
[d] HCC = Hierarchical Condition Category (HCC) risk adjustment model used by Medicare. Higher scores indicate a population of beneficiaries with more chronic conditions
