# Supplementary material for: Visualizing nationwide variation in medicare Part D prescribing patterns
Source: BMC Med Inform Decis Mak. 2018 Nov 19;18:103. doi: 10.1186/s12911-018-0670-2 (PMC6245567; doi:10.1186/s12911-018-0670-2)
Supplement: Supplementary file 3 — Table S1. Differences in high-prescribing provider fractions by geographic region. Table quantifies the fraction of high prescribing Medicare prescribers by United States Administrative Region (see Additional file 1: Figure S1 for region definitions). (PDF 60.6 kb) [file 12911_2018_670_MOESM3_ESM.pdf]

Table S1: Differences in high-prescribing provider fractions by geographic region

Region	States	n =	Median	Min	Max	PAC	ESC	WSC	MTN	NE	SATL	WNC	ENC
Pacific	AK, CA, HI, OR, WA	5	0.19	0.0000	9.8000								
East South Central	AL, KY, MS, TN	4	4.47	2.3300	5.9200	0.1113							
West South Central	AR, LA, OK, TX	4	1.94	1.1200	6.5000	0.1113	0.1939						
Mountain	AZ, CO, ID, MT, NM, NV, UT, WY	8	0.10	0.0000	0.4800	0.5059	0.0049	0.0049					
New England	CT, MA, ME, NH, RI, VT	6	0.29	0.0000	1.2600	0.8535	0.0075	0.0139	0.5130				
South Atlantic	DC, DE, FL, GA, MD, NC, SC, VA, WV	9	1.41	0.0000	12.4200	0.3485	0.1218	0.4869	0.0470	0.1218			
West North Central	IA, KS, MN, MO, ND, NE, SD	7	0.34	0.0000	3.8800	0.9340	0.0099	0.0279	0.5161	0.8253	0.1314		
East North Central	IL, IN, MI, OH, WI	5	1.99	0.3400	5.3800	0.1437	0.1113	0.9025	0.0042	0.0222	0.4222	0.0481	
Middle Atlantic	NJ, NY, PA	3	3.93	1.3100	4.3600	0.1360	0.3768	0.5959	0.0104	0.0138	0.4588	0.0206	0.5510

p-values calculated by the Mann-Whitney U test, with significant values shown in red.

A map of the U.S. Census regions can be found in Supplemental Figure 2.

US State Abbreviations: AL, Alabama; AK, Alaska; AZ, Arizona; AR, Arkansas; CA, California; CO, Colorado; CT, Connecticut; DE, Delaware; FL, Florida; GA, Georgia; HI, Hawaii; ID, Idaho; IL, Illinois; IN, Indiana; IA, Iowa; KS, Kansas; KY, Kentucky; LA, Louisiana; ME, Maine; MD, Maryland; MA, Massachusetts; MI, Michigan; MN, Minnesota; MS, Mississippi; MO, Missouri; MT, Montana; NE, Nebraska; NV, Nevada; NH, New Hampshire; NJ, New Jersey; NM, New Mexico; NY, New York; NC, North Carolina; ND, North Dakota; OH, Ohio; OK, Oklahoma; OR, Oregon; PA, Pennsylvania; RI, Rhode Island; SC, South Carolina; SD, South Dakota; TN, Tennessee; TX, Texas; UT, Utah; VT, Vermont; VA, Virginia; WA, Washington; WV, West Virginia; WI, Wisconsin; WY, Wyoming; DC, Washington DC
