# Supplementary figures and images for: Visualizing nationwide variation in medicare Part D prescribing patterns
Source: BMC Med Inform Decis Mak. 2018 Nov 19;18:103. doi: 10.1186/s12911-018-0670-2 (PMC6245567; doi:10.1186/s12911-018-0670-2)

Provider X Drug

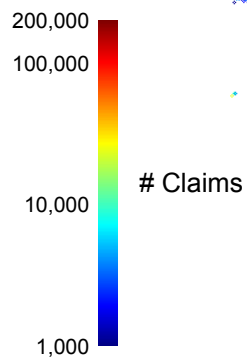

Supplement: Supplementary file 5 — Figure S4. t-SNE plot showing distribution of claim volume per provider. This t-SNE plot is based on the provider by drug matrix, as shown in Fig. 3a. Color corresponds to the Log10 of claims per provider (each represented by a dot). (PDF 899 kb) [file 12911_2018_670_MOESM5_ESM.pdf]

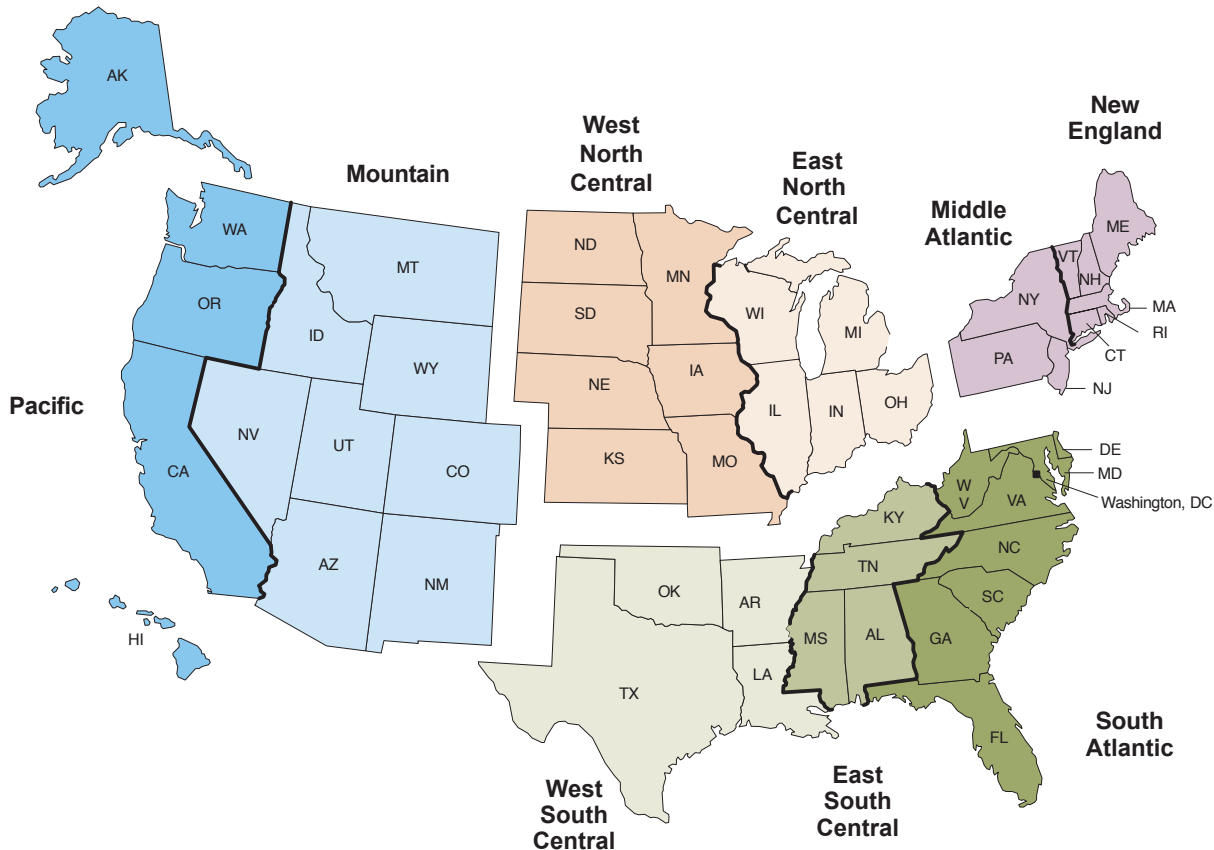

Supplement: Supplementary file 8 — Figure S2. United States Census Regions. Map of United States Census Regions used for geographic data comparisons. Adapted from the United States Census Bureau. (PDF 249 kb) [file 12911_2018_670_MOESM8_ESM.pdf]

$$R^2 = 0.221885$$

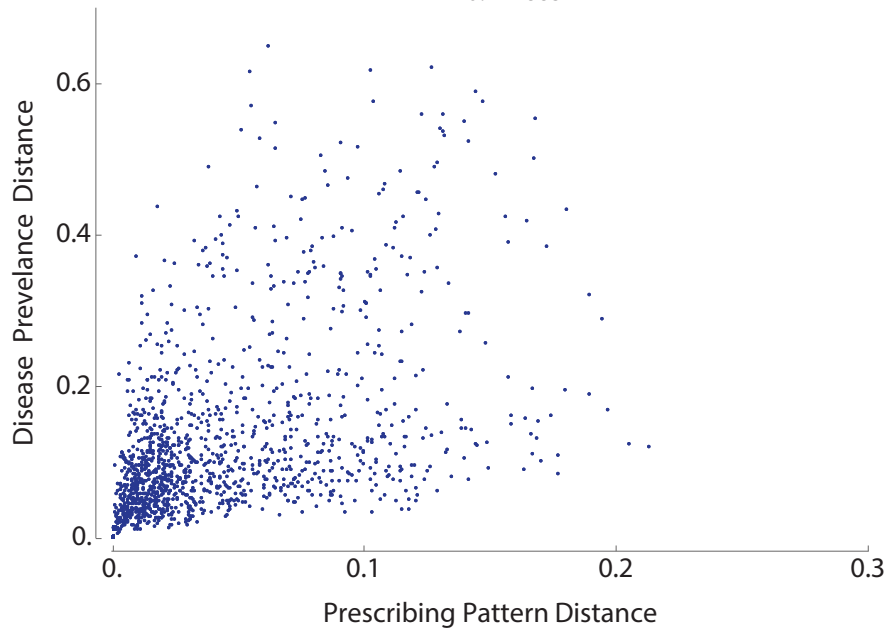

Supplement: Supplementary file 11 — Figure S9. Comparison of between state mutidimensional distance matrices for prescribing pattern versus disease prevalence. Provider specific data for drug class prescribing patterns (n=68 drug classes) and provider-specific patient disease prevalence (n=13 diseases) were obtained from Medicare public use files. Disease prevalence figures included dementia, asthma, atrial fibrillation, cancer, depression, diabetes, chronic obstructive pulmonary disease, chronic kidney disease, heart failure, hyperlipidemia, hypertension, ischemic heart disease, and stroke. Medicare providers with ≥ 1000 Medicare prescriptions in 2013 (n=207,158) and complete data were grouped by state (50 US states, the District of Columbia, and Puerto Rico). We then calculated the mean feature vector prescribing pattern and provider patient-specific disease prevalence values for each state’s providers. To test whether the multi-dimensional drug prescribing pattern differences were correlated with multi-dimensional disease prevelence, we calculated the Euclidean n-dimensional matrix of distances between each pair of states for both prescribing pattern distances and disease prevalence distances. Thus, that states with similar Medicare prescribing patterns should have have small multi-dimensional Euclidean distances, while those that differ would have large distances. A similar relationship would exist for n-dimensional distances calculated using the disease prevalence feature vector; pairs of states with similar prevalence of diseases would have small n-dimensional Euclidean distances. We then tested the correlation between disease prevalence and prescribing pattern distances by analysis of variance, finding an R2=0.22185, indicating that variation in prescribing patterns between states cannot be explained simply by variance in disease prevalence. (PDF 1370 kb) [file 12911_2018_670_MOESM11_ESM.pdf]
