# Supplementary material for: Visualizing nationwide variation in medicare Part D prescribing patterns
Source: BMC Med Inform Decis Mak. 2018 Nov 19;18:103. doi: 10.1186/s12911-018-0670-2 (PMC6245567; doi:10.1186/s12911-018-0670-2)
Supplement: Supplementary file 6 — Figure S6. Unidimensional bar graphs of medication class prescribing frequency by region. Bar graphs of each of the top 10 medication classes prescribed (by percentage of individual prescriber prescriptions) for each of 24 medical specialty groupings, plotted for each of 10 Federal Regions. Note that drug class prescribing percentages are mean levels, and truncated at 21% to make the visualizations informative. (ZIP 5280 kb) [file 12911_2018_670_MOESM6_ESM.zip › Additional file 8/S6_P1R4.pdf]

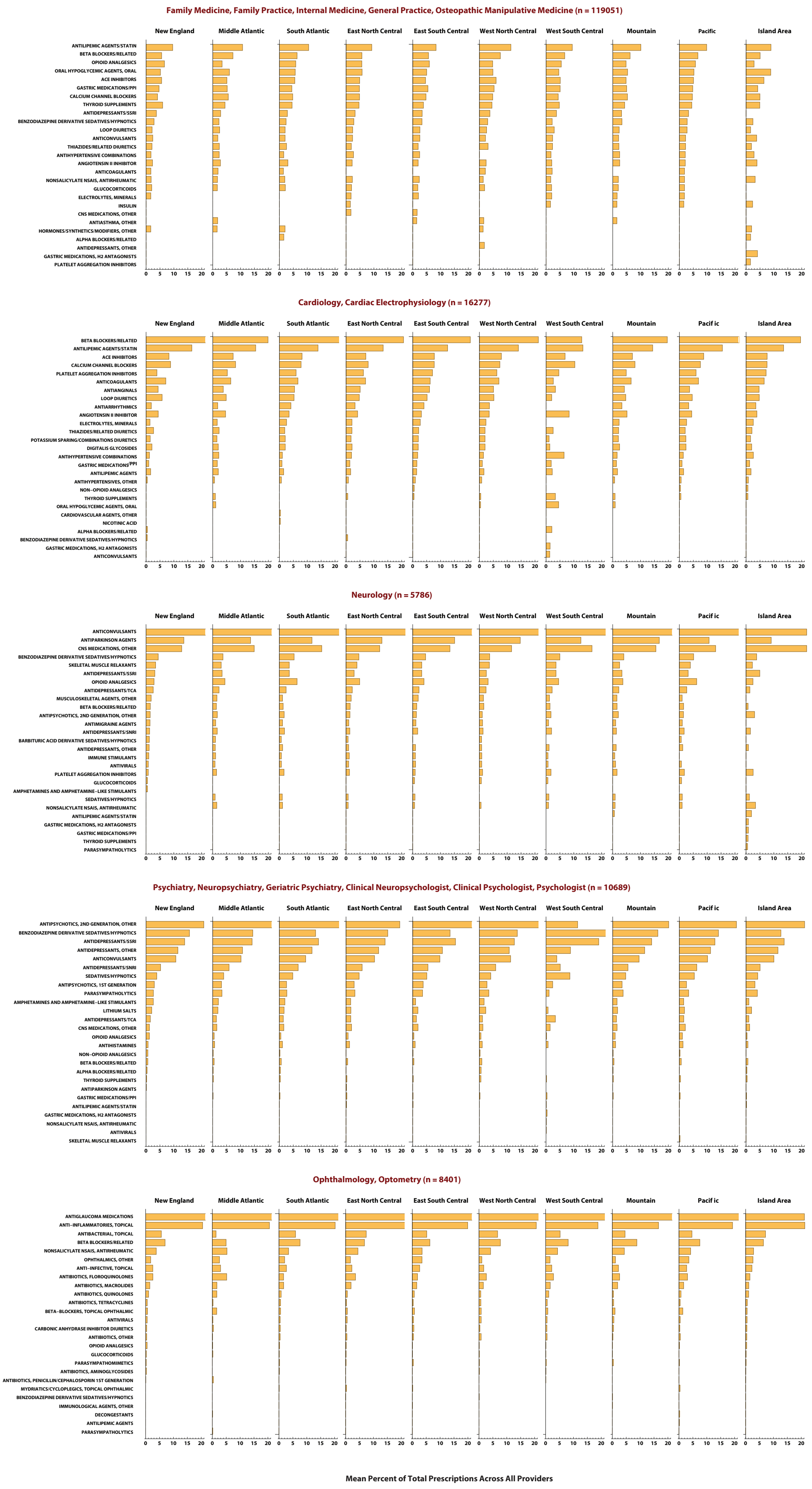
Family Medicine, Family Practice, Internal Medicine, General Practice, Osteopathic Manipulative Medicine (n = 119051)
New England
Middle Atlantic
South Atlantic
East North Central
East South Central
West North Central
West South Central
Mountain
Pacific
Island Area
ANTILIPEMIC AGENTS/STATIN
BETA BLOCKERS/RELATED
OPIOID ANALGESICS
ORAL HYPOGLYCEMIC AGENTS, ORAL
ACE INHIBITORS
GASTRIC MEDICATIONS/PPI
CALCIUM CHANNEL BLOCKERS
THYROID SUPPLEMENTS
ANTIDEPRESSANTS/SSRI
BENZODIAZEPINE DERIVATIVE SEDATIVES/HYPNOTICS
LOOP DIURETICS
ANTICONVULSANTS
THIAZIDES/RELATED DIURETICS
ANTIHYPERTENSIVE COMBINATIONS
ANGIOTENSIN II INHIBITOR
ANTICOAGULANTS
NONSALICYLATE NSAIS, ANTIRHEUMATIC
GLUCOCORTICOIDS
ELECTROLYTES, MINERALS
INSULIN
CNS MEDICATIONS, OTHER
ANTIASTHMA, OTHER
HORMONES/SYNTHETICS/MODIFIERS, OTHER
ALPHA BLOCKERS/RELATED
ANTIDEPRESSANTS, OTHER
GASTRIC MEDICATIONS, H2 ANTAGONISTS
PLATELET AGGREGATION INHIBITORS
0 5 10 15 20
Cardiology, Cardiac Electrophysiology (n = 16277)
New England
Middle Atlantic
South Atlantic
East North Central
East South Central
West North Central
West South Central
Mountain
Pacif ic
Island Area
BETA BLOCKERS/RELATED
ANTILIPEMIC AGENTS/STATIN
ACE INHIBITORS
CALCIUM CHANNEL BLOCKERS
PLATELET AGGREGATION INHIBITORS
ANTICOAGULANTS
ANTIANGINALS
LOOP DIURETICS
ANTIARRHYTHMICS
ANGIOTENSIN II INHIBITOR
ELECTROLYTES, MINERALS
THIAZIDES/RELATED DIURETICS
POTASSIUM SPARING/COMBINATIONS DIURETICS
DIGITALIS GLYCOSIDES
ANTIHYPERTENSIVE COMBINATIONS
GASTRIC MEDICATIONS/PPI
ANTILIPEMIC AGENTS
ANTIHYPERTENSIVES, OTHER
NON−OPIOID ANALGESICS
THYROID SUPPLEMENTS
ORAL HYPOGLYCEMIC AGENTS, ORAL
CARDIOVASCULAR AGENTS, OTHER
NICOTINIC ACID
ALPHA BLOCKERS/RELATED
BENZODIAZEPINE DERIVATIVE SEDATIVES/HYPNOTICS
GASTRIC MEDICATIONS, H2 ANTAGONISTS
ANTICONVULSANTS
0 5 10 15 20
Neurology (n = 5786)
New England
Middle Atlantic
South Atlantic
East North Central
East South Central
West North Central
West South Central
Mountain
Pacif ic
Island Area
ANTICONVULSANTS
ANTIPARKINSON AGENTS
CNS MEDICATIONS, OTHER
BENZODIAZEPINE DERIVATIVE SEDATIVES/HYPNOTICS
SKELETAL MUSCLE RELAXANTS
ANTIDEPRESSANTS/SSRI
OPIOID ANALGESICS
ANTIDEPRESSANTS/TCA
MUSCULOSKELETAL AGENTS, OTHER
BETA BLOCKERS/RELATED
ANTIPSYCHOTICS, 2ND GENERATION, OTHER
ANTIMIGRAINE AGENTS
ANTIDEPRESSANTS/SNRI
BARBITURIC ACID DERIVATIVE SEDATIVES/HYPNOTICS
ANTIDEPRESSANTS, OTHER
IMMUNE STIMULANTS
ANTIVIRALS
PLATELET AGGREGATION INHIBITORS
GLUCOCORTICOIDS
AMPHETAMINES AND AMPHETAMINE−LIKE STIMULANTS
SEDATIVES/HYPNOTICS
NONSALICYLATE NSAIS, ANTIRHEUMATIC
ANTILIPEMIC AGENTS/STATIN
GASTRIC MEDICATIONS, H2 ANTAGONISTS
GASTRIC MEDICATIONS/PPI
THYROID SUPPLEMENTS
PARASYMPATHOLYTICS
0 5 10 15 20
Psychiatry, Neuropsychiatry, Geriatric Psychiatry, Clinical Neuropsychologist, Clinical Psychologist, Psychologist (n = 10689)
New England
Middle Atlantic
South Atlantic
East North Central
East South Central
West North Central
West South Central
Mountain
Pacif ic
Island Area
ANTIPSYCHOTICS, 2ND GENERATION, OTHER
BENZODIAZEPINE DERIVATIVE SEDATIVES/HYPNOTICS
ANTIDEPRESSANTS/SSRI
ANTIDEPRESSANTS, OTHER
ANTICONVULSANTS
ANTIDEPRESSANTS/SNRI
SEDATIVES/HYPNOTICS
ANTIPSYCHOTICS, 1ST GENERATION
PARASYMPATHOLYTICS
AMPHETAMINES AND AMPHETAMINE−LIKE STIMULANTS
LITHIUM SALTS
ANTIDEPRESSANTS/TCA
CNS MEDICATIONS, OTHER
OPIOID ANALGESICS
ANTIHISTAMINES
NON−OPIOID ANALGESICS
BETA BLOCKERS/RELATED
ALPHA BLOCKERS/RELATED
THYROID SUPPLEMENTS
ANTIPARKINSON AGENTS
GASTRIC MEDICATIONS/PPI
ANTILIPEMIC AGENTS/STATIN
GASTRIC MEDICATIONS, H2 ANTAGONISTS
NONSALICYLATE NSAIS, ANTIRHEUMATIC
ANTIVIRALS
SKELETAL MUSCLE RELAXANTS
0 5 10 15 20
Ophthalmology, Optometry (n = 8401)
New England
Middle Atlantic
South Atlantic
East North Central
East South Central
West North Central
West South Central
Mountain
Pacific
Island Area
ANTIGLAUCOMA MEDICATIONS
ANTI−INFLAMMATORIES, TOPICAL
ANTIBACTERIAL, TOPICAL
BETA BLOCKERS/RELATED
NONSALICYLATE NSAIS, ANTIRHEUMATIC
OPHTHALMICS, OTHER
ANTI−INFECTIVE, TOPICAL
ANTIBIOTICS, FLOROQUINOLONES
ANTIBIOTICS, MACROLIDES
ANTIBIOTICS, QUINOLONES
ANTIBIOTICS, TETRACYCLINES
BETA−BLOCKERS, TOPICAL OPHTHALMIC
ANTIVIRALS
CARBONIC ANHYDRASE INHIBITOR DIURETICS
ANTIBIOTICS, OTHER
OPIOID ANALGESICS
GLUCOCORTICOIDS
PARASYMPATHOMIMETICS
ANTIBIOTICS, AMINOGLYCOSIDES
ANTIBIOTICS, PENICILLIN/CEPHALOSPORIN 1ST GENERATION
MYDRIATICS/CYCLOPLEGICS, TOPICAL OPHTHALMIC
BENZODIAZEPINE DERIVATIVE SEDATIVES/HYPNOTICS
IMMUNOLOGICAL AGENTS, OTHER
DECONGESTANTS
ANTILIPEMIC AGENTS
PARASYMPATHOLYTICS
0 5 10 15 20
Mean Percent of Total Prescriptions Across All Providers
