# Supplementary material for: Visualizing nationwide variation in medicare Part D prescribing patterns
Source: BMC Med Inform Decis Mak. 2018 Nov 19;18:103. doi: 10.1186/s12911-018-0670-2 (PMC6245567; doi:10.1186/s12911-018-0670-2)
Supplement: Supplementary file 6 — Figure S6. Unidimensional bar graphs of medication class prescribing frequency by region. Bar graphs of each of the top 10 medication classes prescribed (by percentage of individual prescriber prescriptions) for each of 24 medical specialty groupings, plotted for each of 10 Federal Regions. Note that drug class prescribing percentages are mean levels, and truncated at 21% to make the visualizations informative. (ZIP 5280 kb) [file 12911_2018_670_MOESM6_ESM.zip › Additional file 8/S6_P3R4.pdf]

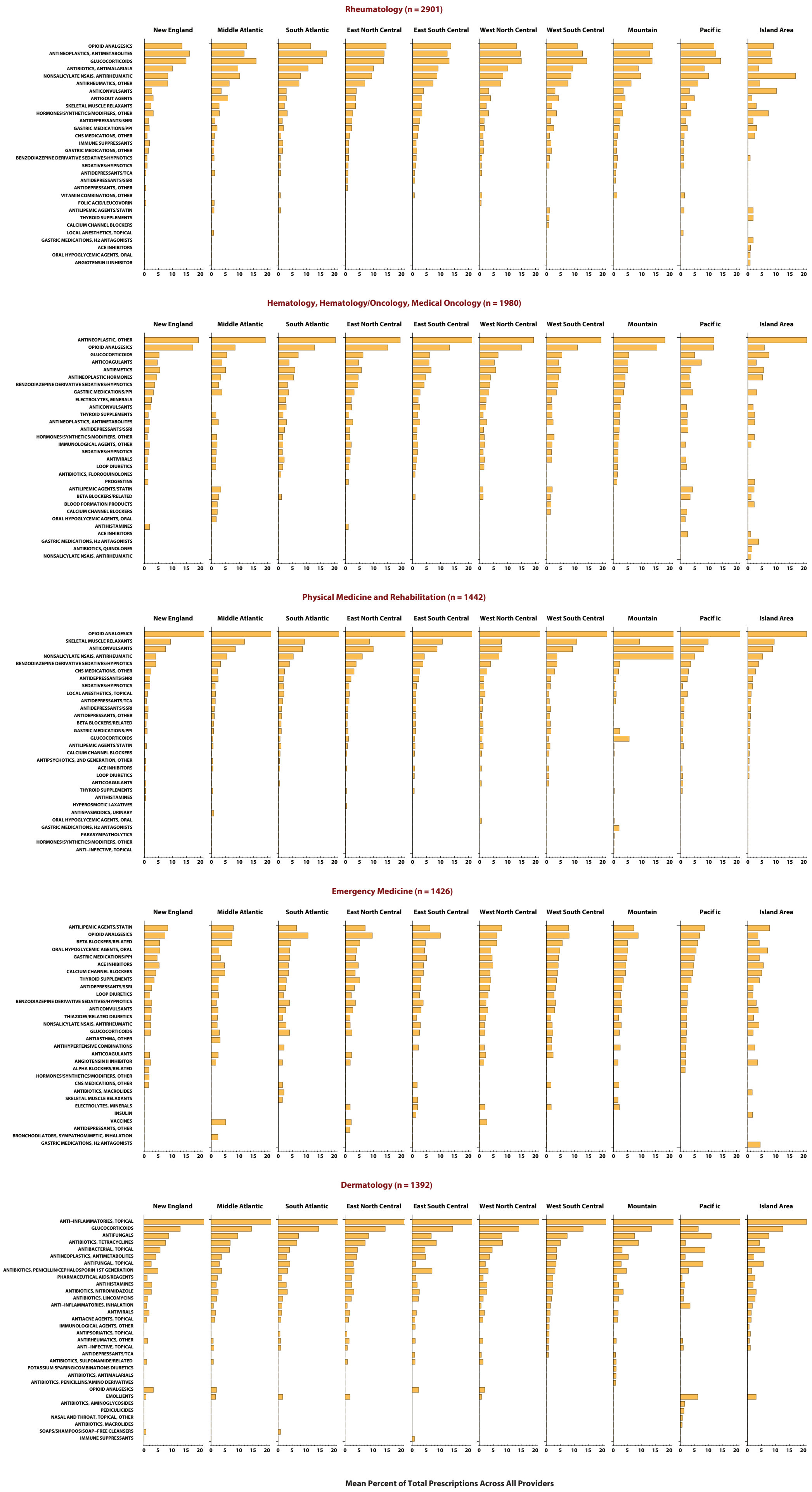

Rheumatology (n = 2901)
New England
Middle Atlantic
South Atlantic
East North Central
East South Central
West North Central
West South Central
Mountain
Pacif ic
Island Area
OPIOID ANALGESICS
ANTINEOPLASTICS, ANTIMETABOLITES
GLUCOCORTICOIDS
ANTIBIOTICS, ANTIMALARIALS
NONSALICYLATE NSAIS, ANTIRHEUMATIC
ANTIRHEUMATICS, OTHER
ANTICONVULSANTS
ANTIGOUT AGENTS
SKELETAL MUSCLE RELAXANTS
HORMONES/SYNTHETICS/MODIFIERS, OTHER
ANTIDEPRESSANTS/SNRI
GASTRIC MEDICATIONS/PPI
CNS MEDICATIONS, OTHER
IMMUNE SUPPRESSANTS
GASTRIC MEDICATIONS, OTHER
BENZODIAZEPINE DERIVATIVE SEDATIVES/HYPNOTICS
SEDATIVES/HYPNOTICS
ANTIDEPRESSANTS/TCA
ANTIDEPRESSANTS/SSRI
ANTIDEPRESSANTS, OTHER
VITAMIN COMBINATIONS, OTHER
FOLIC ACID/LEUCOVORIN
ANTILIPEMIC AGENTS/STATIN
THYROID SUPPLEMENTS
CALCIUM CHANNEL BLOCKERS
LOCAL ANESTHETICS, TOPICAL
GASTRIC MEDICATIONS, H2 ANTAGONISTS
ACE INHIBITORS
ORAL HYPOGLYCEMIC AGENTS, ORAL
ANGIOTENSIN II INHIBITOR
0 5 10 15 20
Hematology, Hematology/Oncology, Medical Oncology (n = 1980)
ANTINEOPLASTIC, OTHER
OPIOID ANALGESICS
GLUCOCORTICOIDS
ANTICOAGULANTS
ANTIEMETICS
ANTINEOPLASTIC HORMONES
BENZODIAZEPINE DERIVATIVE SEDATIVES/HYPNOTICS
GASTRIC MEDICATIONS/PPI
ELECTROLYTES, MINERALS
ANTICONVULSANTS
THYROID SUPPLEMENTS
ANTINEOPLASTICS, ANTIMETABOLITES
ANTIDEPRESSANTS/SSRI
HORMONES/SYNTHETICS/MODIFIERS, OTHER
IMMUNOLOGICAL AGENTS, OTHER
SEDATIVES/HYPNOTICS
ANTIVIRALS
LOOP DIURETICS
ANTIBIOTICS, FLOROQUINOLONES
PROGESTINS
ANTILIPEMIC AGENTS/STATIN
BETA BLOCKERS/RELATED
BLOOD FORMATION PRODUCTS
CALCIUM CHANNEL BLOCKERS
ORAL HYPOGLYCEMIC AGENTS, ORAL
ANTIHISTAMINES
ACE INHIBITORS
GASTRIC MEDICATIONS, H2 ANTAGONISTS
ANTIBIOTICS, QUINOLONES
NONSALICYLATE NSAIS, ANTIRHEUMATIC
Physical Medicine and Rehabilitation (n = 1442)
OPIOID ANALGESICS
SKELETAL MUSCLE RELAXANTS
ANTICONVULSANTS
NONSALICYLATE NSAIS, ANTIRHEUMATIC
BENZODIAZEPINE DERIVATIVE SEDATIVES/HYPNOTICS
CNS MEDICATIONS, OTHER
ANTIDEPRESSANTS/SNRI
SEDATIVES/HYPNOTICS
LOCAL ANESTHETICS, TOPICAL
ANTIDEPRESSANTS/TCA
ANTIDEPRESSANTS/SSRI
ANTIDEPRESSANTS, OTHER
BETA BLOCKERS/RELATED
GASTRIC MEDICATIONS/PPI
GLUCOCORTICOIDS
ANTILIPEMIC AGENTS/STATIN
CALCIUM CHANNEL BLOCKERS
ANTIPSYCHOTICS, 2ND GENERATION, OTHER
ACE INHIBITORS
LOOP DIURETICS
ANTICOAGULANTS
THYROID SUPPLEMENTS
ANTIHISTAMINES
HYPEROSMOTIC LAXATIVES
ANTISPASMODICS, URINARY
ORAL HYPOGLYCEMIC AGENTS, ORAL
GASTRIC MEDICATIONS, H2 ANTAGONISTS
PARASYMPATHOLYTICS
HORMONES/SYNTHETICS/MODIFIERS, OTHER
ANTI-INFECTIVE, TOPICAL
Emergency Medicine (n = 1426)
ANTILIPEMIC AGENTS/STATIN
OPIOID ANALGESICS
BETA BLOCKERS/RELATED
ORAL HYPOGLYCEMIC AGENTS, ORAL
GASTRIC MEDICATIONS/PPI
ACE INHIBITORS
CALCIUM CHANNEL BLOCKERS
THYROID SUPPLEMENTS
ANTIDEPRESSANTS/SSRI
LOOP DIURETICS
BENZODIAZEPINE DERIVATIVE SEDATIVES/HYPNOTICS
ANTICONVULSANTS
THIAZIDES/RELATED DIURETICS
NONSALICYLATE NSAIS, ANTIRHEUMATIC
GLUCOCORTICOIDS
ANTIASTHMA, OTHER
ANTIHYPERTENSIVE COMBINATIONS
ANTICOAGULANTS
ANGIOTENSIN II INHIBITOR
ALPHA BLOCKERS/RELATED
HORMONES/SYNTHETICS/MODIFIERS, OTHER
CNS MEDICATIONS, OTHER
ANTIBIOTICS, MACROLIDES
SKELETAL MUSCLE RELAXANTS
ELECTROLYTES, MINERALS
INSULIN
VACCINES
ANTIDEPRESSANTS, OTHER
BRONCHODILATORS, SYMPATHOMIMETIC, INHALATION
GASTRIC MEDICATIONS, H2 ANTAGONISTS
Dermatology (n = 1392)
ANTI-INFLAMMATORIES, TOPICAL
GLUCOCORTICOIDS
ANTIFUNGALS
ANTIBIOTICS, TETRACYCLINES
ANTIBACTERIAL, TOPICAL
ANTINEOPLASTICS, ANTIMETABOLITES
ANTIFUNGAL, TOPICAL
ANTIBIOTICS, PENICILLIN/CEPHALOSPORIN 1ST GENERATION
PHARMACEUTICAL AIDS/REAGENTS
ANTIHISTAMINES
ANTIBIOTICS, NITROIMIDAZOLE
ANTIBIOTICS, LINCOMYCINS
ANTI-INFLAMMATORIES, INHALATION
ANTIVIRALS
ANTIACNE AGENTS, TOPICAL
IMMUNOLOGICAL AGENTS, OTHER
ANTIPSORIATICS, TOPICAL
ANTIRHEUMATICS, OTHER
ANTI-INFECTIVE, TOPICAL
ANTIDEPRESSANTS/TCA
ANTIBIOTICS, SULFONAMIDE/RELATED
POTASSIUM SPARING/COMBINATIONS DIURETICS
ANTIBIOTICS, ANTIMALARIALS
ANTIBIOTICS, PENICILLINS/AMINO DERIVATIVES
OPIOID ANALGESICS
EMOLLIENTS
ANTIBIOTICS, AMINOGLYCOSIDES
PEDICULICIDES
NASAL AND THROAT, TOPICAL, OTHER
ANTIBIOTICS, MACROLIDES
SOAPS/SHAMPOOS/SOAP-FREE CLEANSERS
IMMUNE SUPPRESSANTS
Mean Percent of Total Prescriptions Across All Providers
