# Supplementary material for: Visualizing nationwide variation in medicare Part D prescribing patterns
Source: BMC Med Inform Decis Mak. 2018 Nov 19;18:103. doi: 10.1186/s12911-018-0670-2 (PMC6245567; doi:10.1186/s12911-018-0670-2)
Supplement: Supplementary file 6 — Figure S6. Unidimensional bar graphs of medication class prescribing frequency by region. Bar graphs of each of the top 10 medication classes prescribed (by percentage of individual prescriber prescriptions) for each of 24 medical specialty groupings, plotted for each of 10 Federal Regions. Note that drug class prescribing percentages are mean levels, and truncated at 21% to make the visualizations informative. (ZIP 5280 kb) [file 12911_2018_670_MOESM6_ESM.zip › Additional file 8/S6_P4R4.pdf]

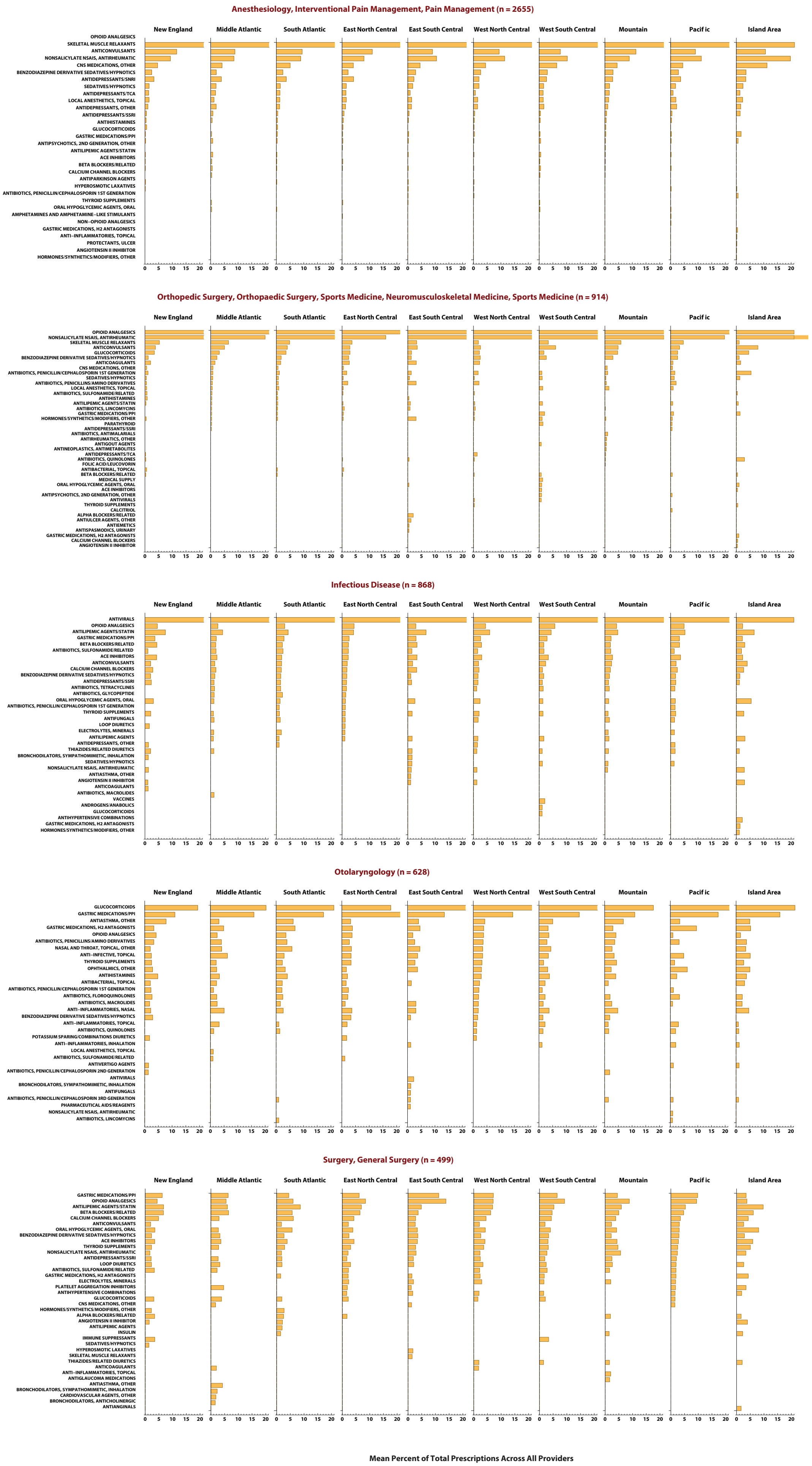
Anesthesiology, Interventional Pain Management, Pain Management (n = 2655)
New England
Middle Atlantic
South Atlantic
East North Central
East South Central
West North Central
West South Central
Mountain
Pacif ic
Island Area
OPIOID ANALGESICS
SKELETAL MUSCLE RELAXANTS
ANTICONVULSANTS
NONSALICYLATE NSAIS, ANTIRHEUMATIC
CNS MEDICATIONS, OTHER
BENZODIAZEPINE DERIVATIVE SEDATIVES/HYPNOTICS
ANTIDEPRESSANTS/SNRI
SEDATIVES/HYPNOTICS
ANTIDEPRESSANTS/TCA
LOCAL ANESTHETICS, TOPICAL
ANTIDEPRESSANTS, OTHER
ANTIDEPRESSANTS/SSRI
ANTIHISTAMINES
GLUCOCORTICOIDS
GASTRIC MEDICATIONS/PPI
ANTIPSYCHOTICS, 2ND GENERATION, OTHER
ANTILIPEMIC AGENTS/STATIN
ACE INHIBITORS
BETA BLOCKERS/RELATED
CALCIUM CHANNEL BLOCKERS
ANTIPARKINSON AGENTS
HYPEROSMOTIC LAXATIVES
ANTIBIOTICS, PENICILLIN/CEPHALOSPORIN 1ST GENERATION
THYROID SUPPLEMENTS
ORAL HYPOGLYCEMIC AGENTS, ORAL
AMPHETAMINES AND AMPHETAMINE-LIKE STIMULANTS
NON-OPIOID ANALGESICS
GASTRIC MEDICATIONS, H2 ANTAGONISTS
ANTI-INFLAMMATORIES, TOPICAL
PROTECTANTS, ULCER
ANGIOTENSIN II INHIBITOR
HORMONES/SYNTHETICS/MODIFIERS, OTHER
0 5 10 15 20
Orthopedic Surgery, Orthopaedic Surgery, Sports Medicine, Neuromusculoskeletal Medicine, Sports Medicine (n = 914)
OPIOID ANALGESICS
NONSALICYLATE NSAIS, ANTIRHEUMATIC
SKELETAL MUSCLE RELAXANTS
ANTICONVULSANTS
GLUCOCORTICOIDS
BENZODIAZEPINE DERIVATIVE SEDATIVES/HYPNOTICS
ANTICOAGULANTS
CNS MEDICATIONS, OTHER
ANTIBIOTICS, PENICILLIN/CEPHALOSPORIN 1ST GENERATION
SEDATIVES/HYPNOTICS
ANTIBIOTICS, PENICILLINS/AMINO DERIVATIVES
LOCAL ANESTHETICS, TOPICAL
ANTIBIOTICS, SULFONAMIDE/RELATED
ANTIHISTAMINES
ANTILIPEMIC AGENTS/STATIN
ANTIBIOTICS, LINCOMYCINS
GASTRIC MEDICATIONS/PPI
HORMONES/SYNTHETICS/MODIFIERS, OTHER
PARATHYROID
ANTIDEPRESSANTS/SSRI
ANTIBIOTICS, ANTIMALARIALS
ANTIRHEUMATICS, OTHER
ANTIGOUT AGENTS
ANTINEOPLASTICS, ANTIMETABOLITES
ANTIDEPRESSANTS/TCA
ANTIBIOTICS, QUINOLONES
FOLIC ACID/LEUCOVORIN
ANTIBACTERIAL, TOPICAL
BETA BLOCKERS/RELATED
MEDICAL SUPPLY
ORAL HYPOGLYCEMIC AGENTS, ORAL
ACE INHIBITORS
ANTIPSYCHOTICS, 2ND GENERATION, OTHER
ANTIVIRALS
THYROID SUPPLEMENTS
CALCITRIOL
ALPHA BLOCKERS/RELATED
ANTIULCER AGENTS, OTHER
ANTIEMETICS
ANTISPASMODICS, URINARY
GASTRIC MEDICATIONS, H2 ANTAGONISTS
CALCIUM CHANNEL BLOCKERS
ANGIOTENSIN II INHIBITOR
Infectious Disease (n = 868)
ANTIVIRALS
OPIOID ANALGESICS
ANTILIPEMIC AGENTS/STATIN
GASTRIC MEDICATIONS/PPI
BETA BLOCKERS/RELATED
ANTIBIOTICS, SULFONAMIDE/RELATED
ACE INHIBITORS
ANTICONVULSANTS
CALCIUM CHANNEL BLOCKERS
BENZODIAZEPINE DERIVATIVE SEDATIVES/HYPNOTICS
ANTIDEPRESSANTS/SSRI
ANTIBIOTICS, TETRACYCLINES
ANTIBIOTICS, GLYCOPEPTIDE
ORAL HYPOGLYCEMIC AGENTS, ORAL
ANTIBIOTICS, PENICILLIN/CEPHALOSPORIN 1ST GENERATION
THYROID SUPPLEMENTS
ANTIFUNGALS
LOOP DIURETICS
ELECTROLYTES, MINERALS
ANTILIPEMIC AGENTS
ANTIDEPRESSANTS, OTHER
THIAZIDES/RELATED DIURETICS
BRONCHODILATORS, SYMPATHOMIMETIC, INHALATION
SEDATIVES/HYPNOTICS
NONSALICYLATE NSAIS, ANTIRHEUMATIC
ANTIASTHMA, OTHER
ANGIOTENSIN II INHIBITOR
ANTICOAGULANTS
ANTIBIOTICS, MACROLIDES
VACCINES
ANDROGENS/ANABOLICS
GLUCOCORTICOIDS
ANTIHYPERTENSIVE COMBINATIONS
GASTRIC MEDICATIONS, H2 ANTAGONISTS
HORMONES/SYNTHETICS/MODIFIERS, OTHER
Otolaryngology (n = 628)
GLUCOCORTICOIDS
GASTRIC MEDICATIONS/PPI
ANTIASTHMA, OTHER
GASTRIC MEDICATIONS, H2 ANTAGONISTS
OPIOID ANALGESICS
ANTIBIOTICS, PENICILLINS/AMINO DERIVATIVES
NASAL AND THROAT, TOPICAL, OTHER
ANTI-INFECTIVE, TOPICAL
THYROID SUPPLEMENTS
OPHTHALMICS, OTHER
ANTIHISTAMINES
ANTIBACTERIAL, TOPICAL
ANTIBIOTICS, PENICILLIN/CEPHALOSPORIN 1ST GENERATION
ANTIBIOTICS, FLOROQUINOLONES
ANTIBIOTICS, MACROLIDES
ANTI-INFLAMMATORIES, NASAL
BENZODIAZEPINE DERIVATIVE SEDATIVES/HYPNOTICS
ANTI-INFLAMMATORIES, TOPICAL
ANTIBIOTICS, QUINOLONES
POTASSIUM SPARING/COMBINATIONS DIURETICS
ANTI-INFLAMMATORIES, INHALATION
LOCAL ANESTHETICS, TOPICAL
ANTIBIOTICS, SULFONAMIDE/RELATED
ANTIVERTIGO AGENTS
ANTIBIOTICS, PENICILLIN/CEPHALOSPORIN 2ND GENERATION
ANTIVIRALS
BRONCHODILATORS, SYMPATHOMIMETIC, INHALATION
ANTIFUNGALS
ANTIBIOTICS, PENICILLIN/CEPHALOSPORIN 3RD GENERATION
PHARMACEUTICAL AIDS/REAGENTS
NONSALICYLATE NSAIS, ANTIRHEUMATIC
ANTIBIOTICS, LINCOMYCINS
Surgery, General Surgery (n = 499)
GASTRIC MEDICATIONS/PPI
OPIOID ANALGESICS
ANTILIPEMIC AGENTS/STATIN
BETA BLOCKERS/RELATED
CALCIUM CHANNEL BLOCKERS
ANTICONVULSANTS
ORAL HYPOGLYCEMIC AGENTS, ORAL
BENZODIAZEPINE DERIVATIVE SEDATIVES/HYPNOTICS
ACE INHIBITORS
THYROID SUPPLEMENTS
NONSALICYLATE NSAIS, ANTIRHEUMATIC
ANTIDEPRESSANTS/SSRI
LOOP DIURETICS
ANTIBIOTICS, SULFONAMIDE/RELATED
GASTRIC MEDICATIONS, H2 ANTAGONISTS
ELECTROLYTES, MINERALS
PLATELET AGGREGATION INHIBITORS
ANTIHYPERTENSIVE COMBINATIONS
GLUCOCORTICOIDS
CNS MEDICATIONS, OTHER
HORMONES/SYNTHETICS/MODIFIERS, OTHER
ALPHA BLOCKERS/RELATED
ANGIOTENSIN II INHIBITOR
ANTILIPEMIC AGENTS
INSULIN
IMMUNE SUPPRESSANTS
SEDATIVES/HYPNOTICS
HYPEROSMOTIC LAXATIVES
SKELETAL MUSCLE RELAXANTS
THIAZIDES/RELATED DIURETICS
ANTICOAGULANTS
ANTI-INFLAMMATORIES, TOPICAL
ANTIGLAUCOMA MEDICATIONS
ANTIASTHMA, OTHER
BRONCHODILATORS, SYMPATHOMIMETIC, INHALATION
CARDIOVASCULAR AGENTS, OTHER
BRONCHODILATORS, ANTICHOLINERGIC
ANTIANGINALS
Mean Percent of Total Prescriptions Across All Providers
