# Supplementary material for: Visualizing nationwide variation in medicare Part D prescribing patterns
Source: BMC Med Inform Decis Mak. 2018 Nov 19;18:103. doi: 10.1186/s12911-018-0670-2 (PMC6245567; doi:10.1186/s12911-018-0670-2)
Supplement: Supplementary file 6 — Figure S6. Unidimensional bar graphs of medication class prescribing frequency by region. Bar graphs of each of the top 10 medication classes prescribed (by percentage of individual prescriber prescriptions) for each of 24 medical specialty groupings, plotted for each of 10 Federal Regions. Note that drug class prescribing percentages are mean levels, and truncated at 21% to make the visualizations informative. (ZIP 5280 kb) [file 12911_2018_670_MOESM6_ESM.zip › Additional file 8/S6_P5R4.pdf]

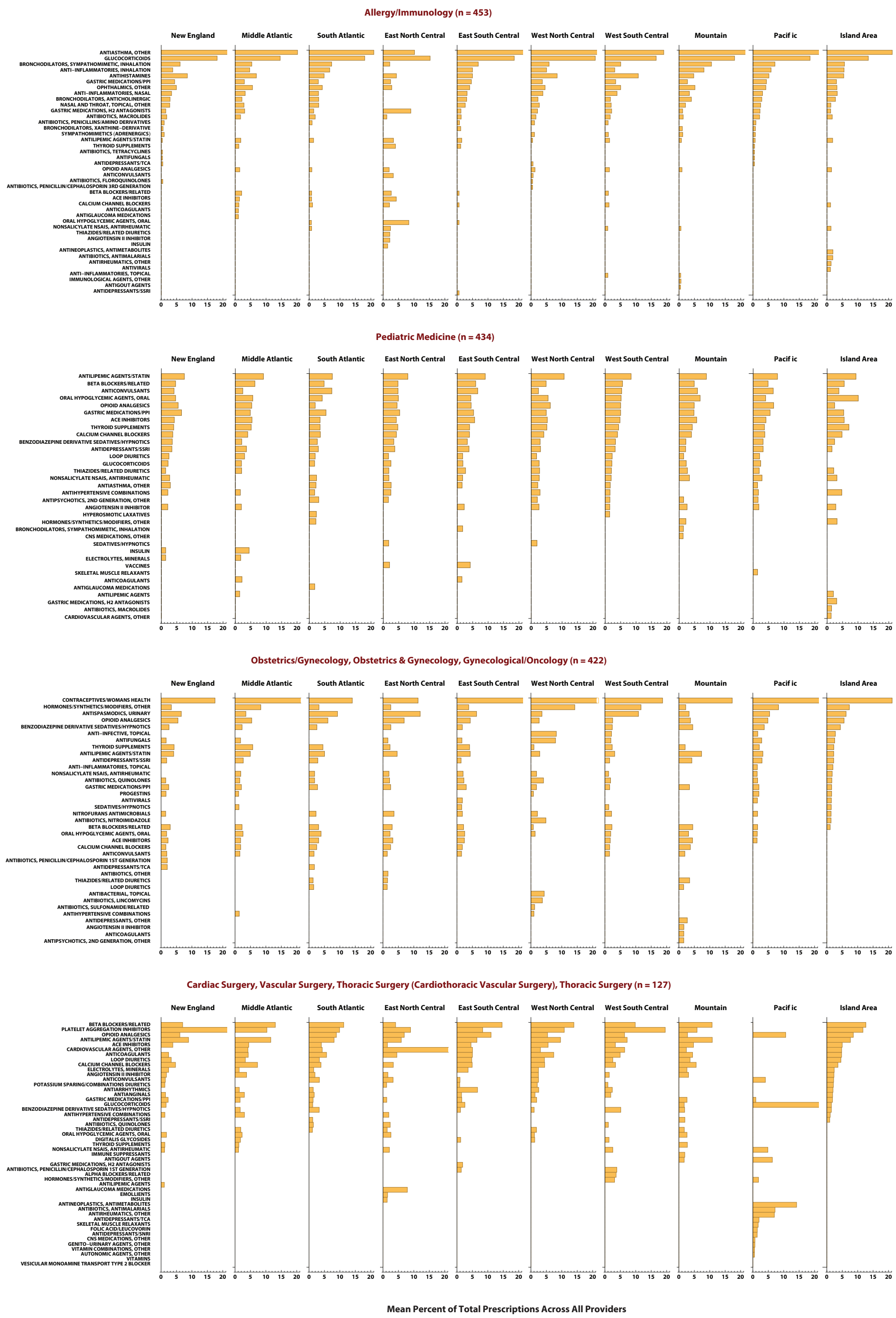
Allergy/Immunology (n = 453)
New England
Middle Atlantic
South Atlantic
East North Central
East South Central
West North Central
West South Central
Mountain
Pacif ic
Island Area
ANTIASTHMA, OTHER
GLUCOCORTICOIDS
BRONCHODILATORS, SYMPATHOMIMETIC, INHALATION
ANTI–INFLAMMATORIES, INHALATION
ANTIHISTAMINES
GASTRIC MEDICATIONS/PPI
OPHTHALMICS, OTHER
ANTI–INFLAMMATORIES, NASAL
BRONCHODILATORS, ANTICHOLINERGIC
NASAL AND THROAT, TOPICAL, OTHER
GASTRIC MEDICATIONS, H2 ANTAGONISTS
ANTIBIOTICS, MACROLIDES
ANTIBIOTICS, PENICILLINS/AMINO DERIVATIVES
BRONCHODILATORS, XANTHINE–DERIVATIVE
SYMPATHOMIMETICS (ADRENERGICS)
ANTILIPEMIC AGENTS/STATIN
THYROID SUPPLEMENTS
ANTIBIOTICS, TETRACYCLINES
ANTIFUNGALS
ANTIDEPRESSANTS/TCA
OPIOID ANALGESICS
ANTICONVULSANTS
ANTIBIOTICS, FLOROQUINOLONES
ANTIBIOTICS, PENICILLIN/CEPHALOSPORIN 3RD GENERATION
BETA BLOCKERS/RELATED
ACE INHIBITORS
CALCIUM CHANNEL BLOCKERS
ANTICOAGULANTS
ANTIGLAUCOMA MEDICATIONS
ORAL HYPOGLYCEMIC AGENTS, ORAL
NONSALICYLATE NSAIS, ANTIRHEUMATIC
THIAZIDES/RELATED DIURETICS
ANGIOTENSIN II INHIBITOR
INSULIN
ANTINEOPLASTICS, ANTIMETABOLITES
ANTIBIOTICS, ANTIMALARIALS
ANTIRHEUMATICS, OTHER
ANTIVIRALS
ANTI–INFLAMMATORIES, TOPICAL
IMMUNOLOGICAL AGENTS, OTHER
ANTIGOUT AGENTS
ANTIDEPRESSANTS/SSRI
0 5 10 15 20
Pediatric Medicine (n = 434)
ANTILIPEMIC AGENTS/STATIN
BETA BLOCKERS/RELATED
ANTICONVULSANTS
ORAL HYPOGLYCEMIC AGENTS, ORAL
OPIOID ANALGESICS
GASTRIC MEDICATIONS/PPI
ACE INHIBITORS
THYROID SUPPLEMENTS
CALCIUM CHANNEL BLOCKERS
BENZODIAZEPINE DERIVATIVE SEDATIVES/HYPNOTICS
ANTIDEPRESSANTS/SSRI
LOOP DIURETICS
GLUCOCORTICOIDS
THIAZIDES/RELATED DIURETICS
NONSALICYLATE NSAIS, ANTIRHEUMATIC
ANTIASTHMA, OTHER
ANTIHYPERTENSIVE COMBINATIONS
ANTIPSYCHOTICS, 2ND GENERATION, OTHER
ANGIOTENSIN II INHIBITOR
HYPEROSMOTIC LAXATIVES
HORMONES/SYNTHETICS/MODIFIERS, OTHER
BRONCHODILATORS, SYMPATHOMIMETIC, INHALATION
CNS MEDICATIONS, OTHER
SEDATIVES/HYPNOTICS
INSULIN
ELECTROLYTES, MINERALS
VACCINES
SKELETAL MUSCLE RELAXANTS
ANTICOAGULANTS
ANTIGLAUCOMA MEDICATIONS
ANTILIPEMIC AGENTS
GASTRIC MEDICATIONS, H2 ANTAGONISTS
ANTIBIOTICS, MACROLIDES
CARDIOVASCULAR AGENTS, OTHER
0 5 10 15 20
Obstetrics/Gynecology, Obstetrics & Gynecology, Gynecological/Oncology (n = 422)
CONTRACEPTIVES/WOMANS HEALTH
HORMONES/SYNTHETICS/MODIFIERS, OTHER
ANTISPASMODICS, URINARY
OPIOID ANALGESICS
BENZODIAZEPINE DERIVATIVE SEDATIVES/HYPNOTICS
ANTI–INFECTIVE, TOPICAL
ANTIFUNGALS
THYROID SUPPLEMENTS
ANTILIPEMIC AGENTS/STATIN
ANTIDEPRESSANTS/SSRI
ANTI–INFLAMMATORIES, TOPICAL
NONSALICYLATE NSAIS, ANTIRHEUMATIC
ANTIBIOTICS, QUINOLONES
GASTRIC MEDICATIONS/PPI
PROGESTINS
ANTIVIRALS
SEDATIVES/HYPNOTICS
NITROFURANS ANTIMICROBIALS
ANTIBIOTICS, NITROIMIDAZOLE
BETA BLOCKERS/RELATED
ORAL HYPOGLYCEMIC AGENTS, ORAL
ACE INHIBITORS
CALCIUM CHANNEL BLOCKERS
ANTICONVULSANTS
ANTIBIOTICS, PENICILLIN/CEPHALOSPORIN 1ST GENERATION
ANTIDEPRESSANTS/TCA
ANTIBIOTICS, OTHER
THIAZIDES/RELATED DIURETICS
LOOP DIURETICS
ANTIBACTERIAL, TOPICAL
ANTIBIOTICS, LINCOMYCINS
ANTIBIOTICS, SULFONAMIDE/RELATED
ANTIHYPERTENSIVE COMBINATIONS
ANTIDEPRESSANTS, OTHER
ANGIOTENSIN II INHIBITOR
ANTICOAGULANTS
ANTIPSYCHOTICS, 2ND GENERATION, OTHER
0 5 10 15 20
Cardiac Surgery, Vascular Surgery, Thoracic Surgery (Cardiothoracic Vascular Surgery), Thoracic Surgery (n = 127)
BETA BLOCKERS/RELATED
PLATELET AGGREGATION INHIBITORS
OPIOID ANALGESICS
ANTILIPEMIC AGENTS/STATIN
ACE INHIBITORS
CARDIOVASCULAR AGENTS, OTHER
ANTICOAGULANTS
LOOP DIURETICS
CALCIUM CHANNEL BLOCKERS
ELECTROLYTES, MINERALS
ANGIOTENSIN II INHIBITOR
ANTICONVULSANTS
POTASSIUM SPARING/COMBINATIONS DIURETICS
ANTIARRHYTHMICS
ANTIANGINALS
GASTRIC MEDICATIONS/PPI
GLUCOCORTICOIDS
BENZODIAZEPINE DERIVATIVE SEDATIVES/HYPNOTICS
ANTIHYPERTENSIVE COMBINATIONS
ANTIDEPRESSANTS/SSRI
ANTIBIOTICS, QUINOLONES
THIAZIDES/RELATED DIURETICS
ORAL HYPOGLYCEMIC AGENTS, ORAL
DIGITALIS GLYCOSIDES
THYROID SUPPLEMENTS
NONSALICYLATE NSAIS, ANTIRHEUMATIC
IMMUNE SUPPRESSANTS
ANTIGOUT AGENTS
GASTRIC MEDICATIONS, H2 ANTAGONISTS
ANTIBIOTICS, PENICILLIN/CEPHALOSPORIN 1ST GENERATION
ALPHA BLOCKERS/RELATED
HORMONES/SYNTHETICS/MODIFIERS, OTHER
ANTILIPEMIC AGENTS
ANTIGLAUCOMA MEDICATIONS
EMOLLIENTS
INSULIN
ANTINEOPLASTICS, ANTIMETABOLITES
ANTIBIOTICS, ANTIMALARIALS
ANTIRHEUMATICS, OTHER
ANTIDEPRESSANTS/TCA
SKELETAL MUSCLE RELAXANTS
FOLIC ACID/LEUCOVORIN
ANTIDEPRESSANTS/SNRI
CNS MEDICATIONS, OTHER
GENITO–URINARY AGENTS, OTHER
VITAMIN COMBINATIONS, OTHER
AUTONOMIC AGENTS, OTHER
VITAMINS
VESICULAR MONOAMINE TRANSPORT TYPE 2 BLOCKER
0 5 10 15 20
Mean Percent of Total Prescriptions Across All Providers
