# Supplementary material for: Visualizing nationwide variation in medicare Part D prescribing patterns
Source: BMC Med Inform Decis Mak. 2018 Nov 19;18:103. doi: 10.1186/s12911-018-0670-2 (PMC6245567; doi:10.1186/s12911-018-0670-2)
Supplement: Supplementary file 7 — Figure S5. Hierarchical clustering. Plots of the 605 clusters identified by hierarchical clustering with linkage using Ward’s minimization criteria. The background is the full t-SNE projection, while each cluster is in red. This 19 page figure is available for download from https://figshare.com/account/projects/24664/articles/5388157. (PDF 19 MB) [file 12911_2018_670_MOESM7_ESM.pdf]

**Cluster 576**
**(n = 4)**
**Family Practice (100.0 %)**

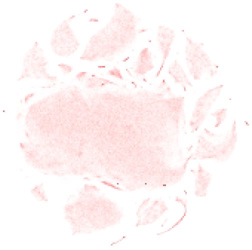

**Cluster 577**
**(n = 4)**
**Internal Medicine (50.0 %)**

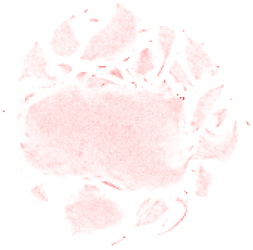

**Cluster 578**
**(n = 3)**
**General Practice (66.7 %)**

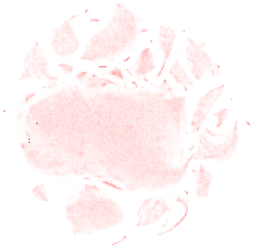

**Cluster 579**
**(n = 3)**
**Otolaryngology (33.3 %)**

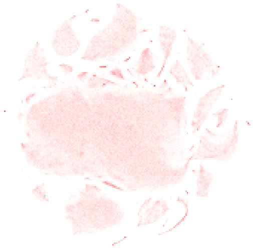

**Cluster 580**
**(n = 3)**
**Internal Medicine (66.7 %)**

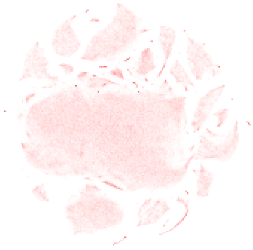

**Cluster 581**
**(n = 3)**
**Internal Medicine (66.7 %)**

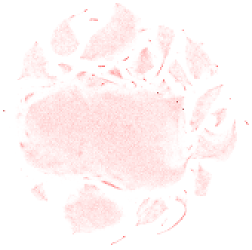

**Cluster 582**
**(n = 3)**
**Ophthalmology (100.0 %)**

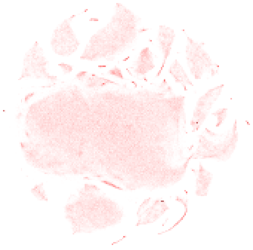

**Cluster 583**
**(n = 3)**
**Internal Medicine (66.7 %)**

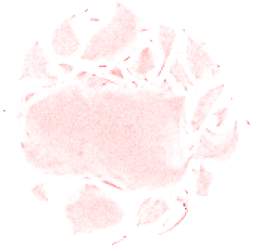

**Cluster 584**
**(n = 3)**
**Nurse Practitioner (66.7 %)**

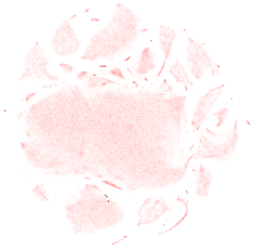

**Cluster 585**
**(n = 3)**
**Ophthalmology (100.0 %)**

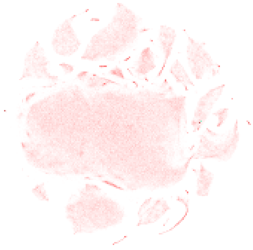

**Cluster 586**
**(n = 2)**
**General Practice (50.0 %)**

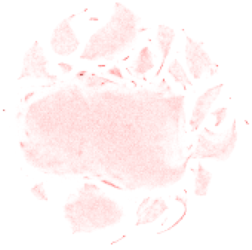

**Cluster 587**
**(n = 2)**
**Ophthalmology (100.0 %)**

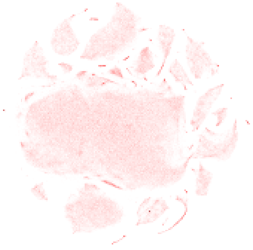

**Cluster 588**
**(n = 2)**
**Obstetrics/Gynecology (50.0 %)**

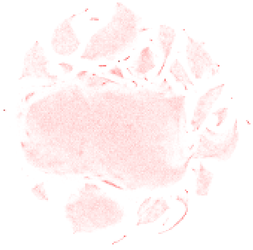

**Cluster 589**
**(n = 2)**
**Nephrology (100.0 %)**

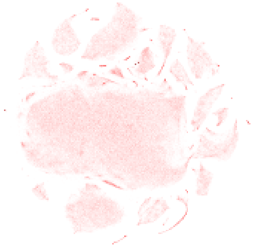

**Cluster 590**
**(n = 2)**
**Nephrology (100.0 %)**

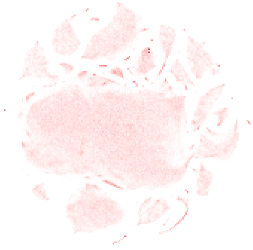

**Cluster 591**
**(n = 2)**
**Cardiology (100.0 %)**

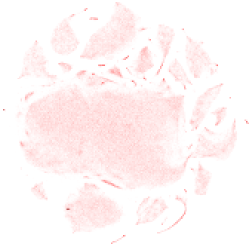

**Cluster 592**
**(n = 2)**
**Pulmonary Disease (100.0 %)**

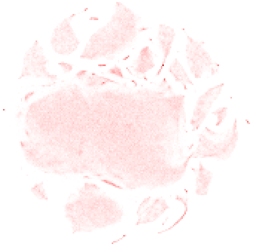

**Cluster 593**
**(n = 2)**
**Neurosurgery (50.0 %)**

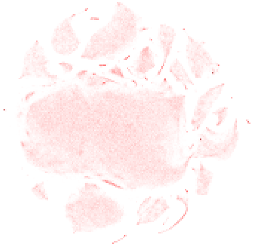

**Cluster 594**
**(n = 2)**
**Gastroenterology (100.0 %)**

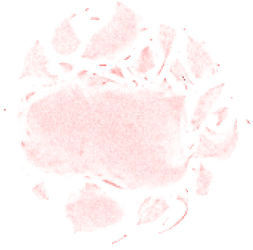

**Cluster 595**
**(n = 2)**
**Nephrology (50.0 %)**

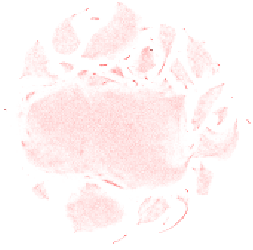

**Cluster 596**
**(n = 2)**
**Family Practice (50.0 %)**

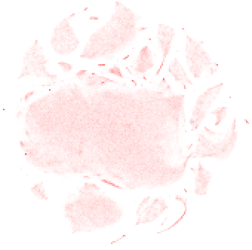

**Cluster 597**
**(n = 2)**
**Urology (100.0 %)**

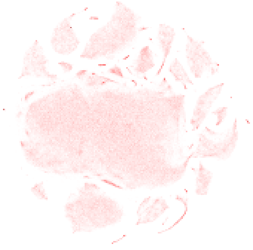

**Cluster 598**
**(n = 2)**
**Psychiatry (50.0 %)**

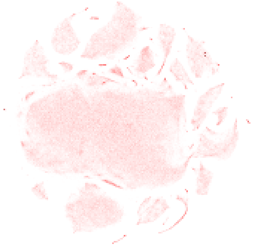

**Cluster 599**
**(n = 1)**
**Cardiology (100.0 %)**

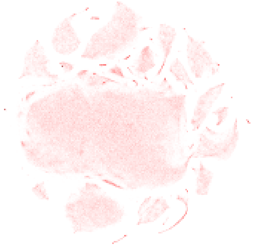

**Cluster 600**
**(n = 1)**
**Neurology (100.0 %)**

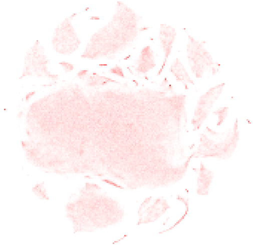

Cluster 551
(n = 7)
Obstetrics/Gynecology (42.9 %)

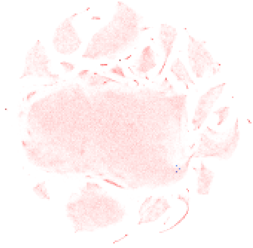

Cluster 552
(n = 7)
Urology (57.1 %)

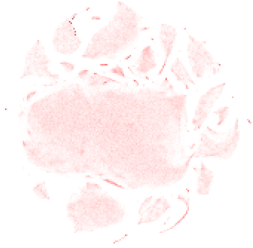

Cluster 553
(n = 7)
Neurology (57.1 %)

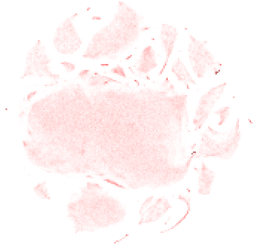

Cluster 554
(n = 7)
Ophthalmology (100.0 %)

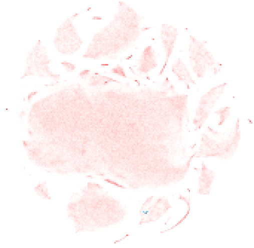

Cluster 555
(n = 7)
Hematology/Oncology (71.4 %)

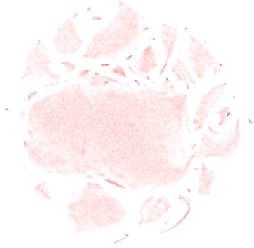

Cluster 556
(n = 6)
Gastroenterology (66.7 %)

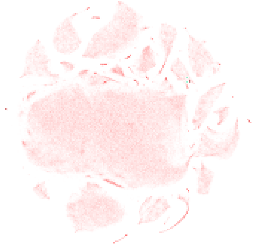

Cluster 557
(n = 6)
Nephrology (83.3 %)

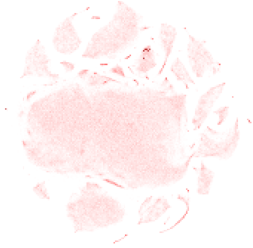

Cluster 558
(n = 6)
Ophthalmology (100.0 %)

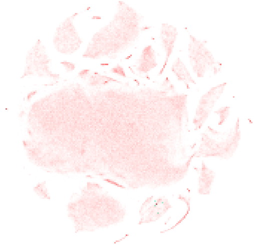

Cluster 559
(n = 6)
Nurse Practitioner (33.3 %)

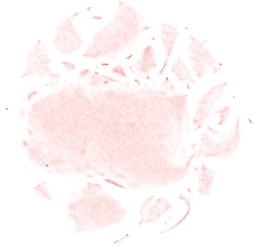

Cluster 560
(n = 6)
Neurology (50.0 %)

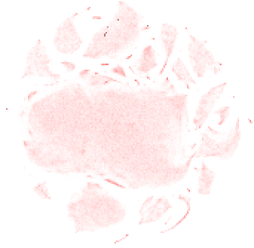

Cluster 561
(n = 6)
Pulmonary Disease (100.0 %)

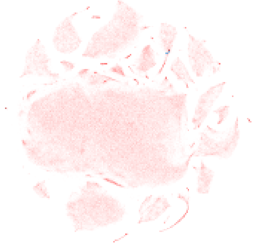

Cluster 562
(n = 6)
Neurology (83.3 %)

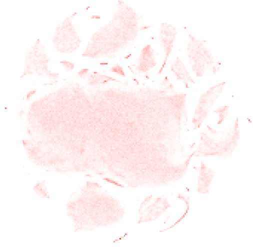

Cluster 563
(n = 6)
Cardiology (66.7 %)

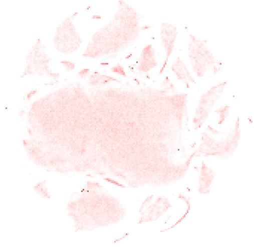

Cluster 564
(n = 5)
Podiatry (40.0 %)

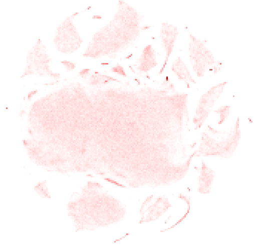

Cluster 565
(n = 5)
Psychiatry (40.0 %)

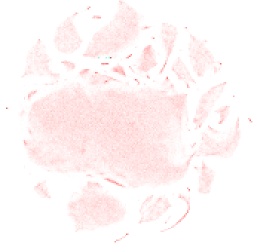

Cluster 566
(n = 5)
Nephrology (100.0 %)

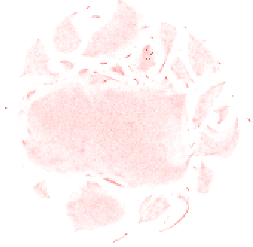

Cluster 567
(n = 5)
Neurology (100.0 %)

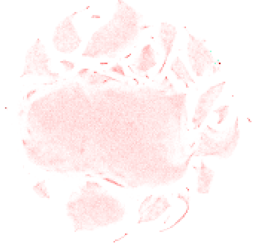

Cluster 568
(n = 5)
Podiatry (20.0 %)

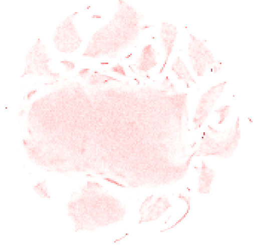

Cluster 569
(n = 4)
Psychiatry (75.0 %)

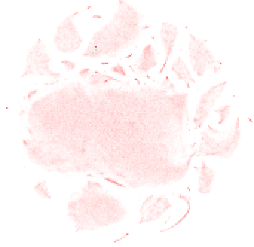

Cluster 570
(n = 4)
Nurse Practitioner (50.0 %)

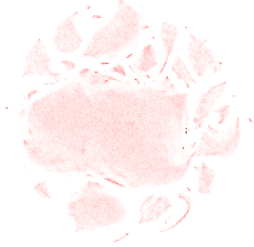

Cluster 571
(n = 4)
Urology (100.0 %)

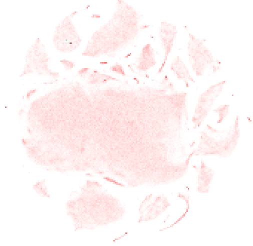

Cluster 572
(n = 4)
Psychiatry & Neurology (50.0 %)

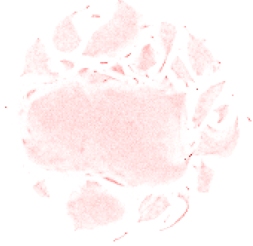

Cluster 573
(n = 4)
Family Practice (75.0 %)

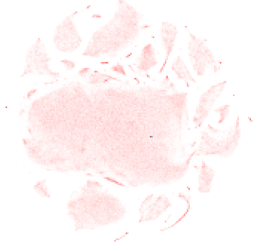

Cluster 574
(n = 4)
Endocrinology (75.0 %)

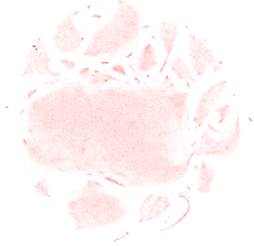

Cluster 575
(n = 4)
Internal Medicine (25.0 %)

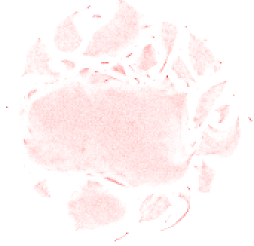

**Cluster 526**
**(n = 11)**
**Nephrology (72.7 %)**

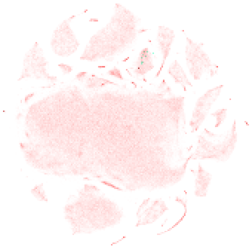

**Cluster 527**
**(n = 11)**
**Pulmonary Disease (72.7 %)**

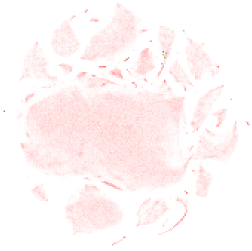

**Cluster 528**
**(n = 11)**
**Cardiology (54.5 %)**

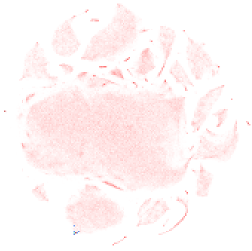

**Cluster 529**
**(n = 11)**
**Nurse Practitioner (54.5 %)**

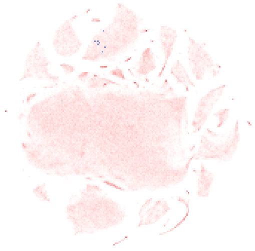

**Cluster 530**
**(n = 11)**
**Neurology (90.9 %)**

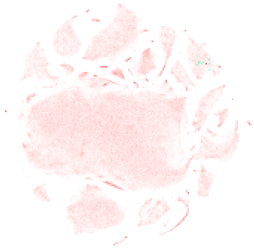

**Cluster 531**
**(n = 11)**
**Rheumatology (90.9 %)**

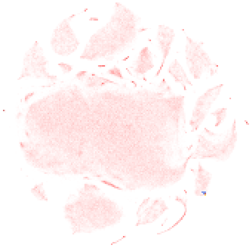

**Cluster 532**
**(n = 10)**
**Internal Medicine (50.0 %)**

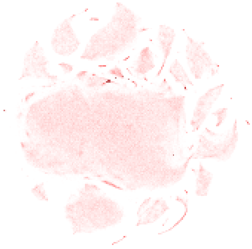

**Cluster 533**
**(n = 10)**
**Ophthalmology (100.0 %)**

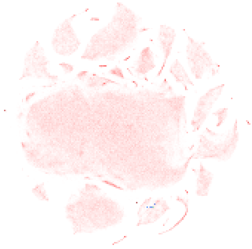

**Cluster 534**
**(n = 10)**
**Family Practice (50.0 %)**

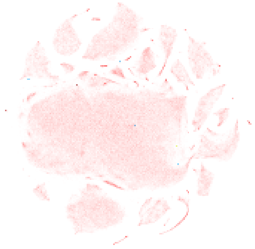

**Cluster 535**
**(n = 10)**
**Internal Medicine (50.0 %)**

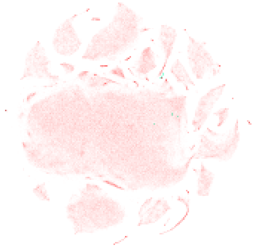

**Cluster 536**
**(n = 9)**
**Family Practice (22.2 %)**

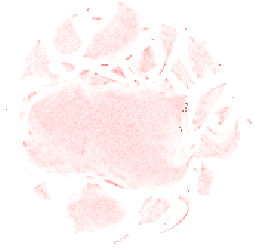

**Cluster 537**
**(n = 9)**
**Ophthalmology (100.0 %)**

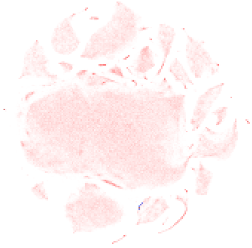

**Cluster 538**
**(n = 9)**
**Nurse Practitioner (33.3 %)**

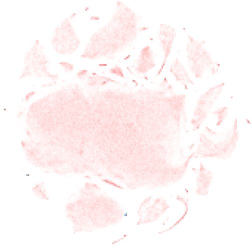

**Cluster 539**
**(n = 9)**
**Family Practice (44.4 %)**

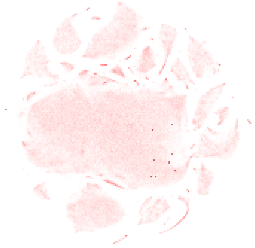

**Cluster 540**
**(n = 9)**
**Gastroenterology (88.9 %)**

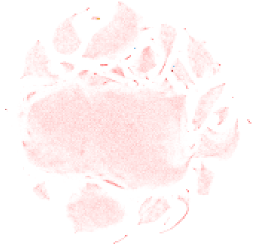

**Cluster 541**
**(n = 9)**
**Psychiatry (55.6 %)**

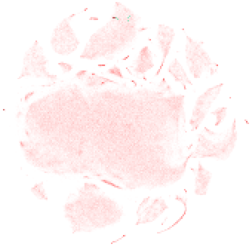

**Cluster 542**
**(n = 9)**
**Cardiology (88.9 %)**

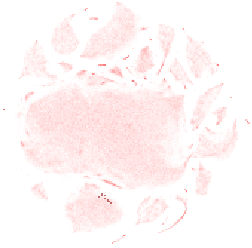

**Cluster 543**
**(n = 8)**
**Urology (87.5 %)**

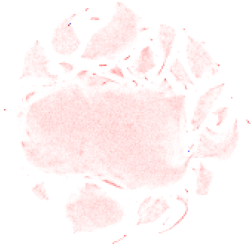

**Cluster 544**
**(n = 8)**
**Gastroenterology (87.5 %)**

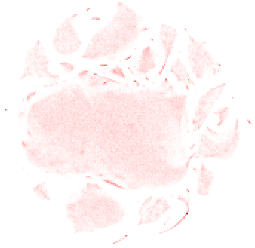

**Cluster 545**
**(n = 8)**
**Internal Medicine (62.5 %)**

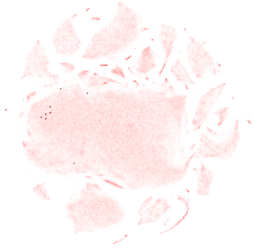

**Cluster 546**
**(n = 8)**
**Internal Medicine (37.5 %)**

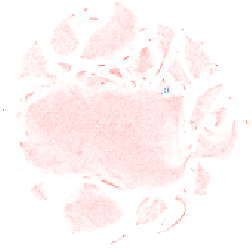

**Cluster 547**
**(n = 8)**
**Hematology/Oncology (87.5 %)**

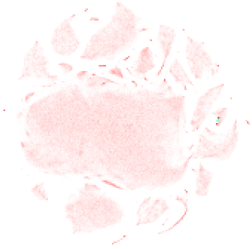

**Cluster 548**
**(n = 8)**
**Internal Medicine (37.5 %)**

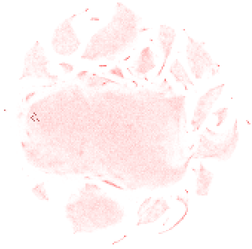

**Cluster 549**
**(n = 8)**
**Gastroenterology (87.5 %)**

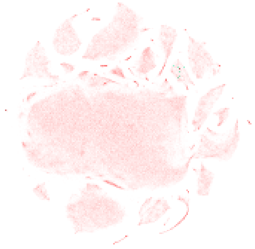

**Cluster 550**
**(n = 8)**
**Podiatry (37.5 %)**

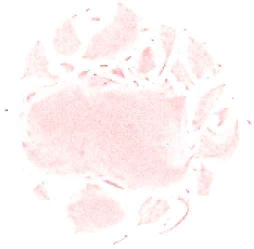

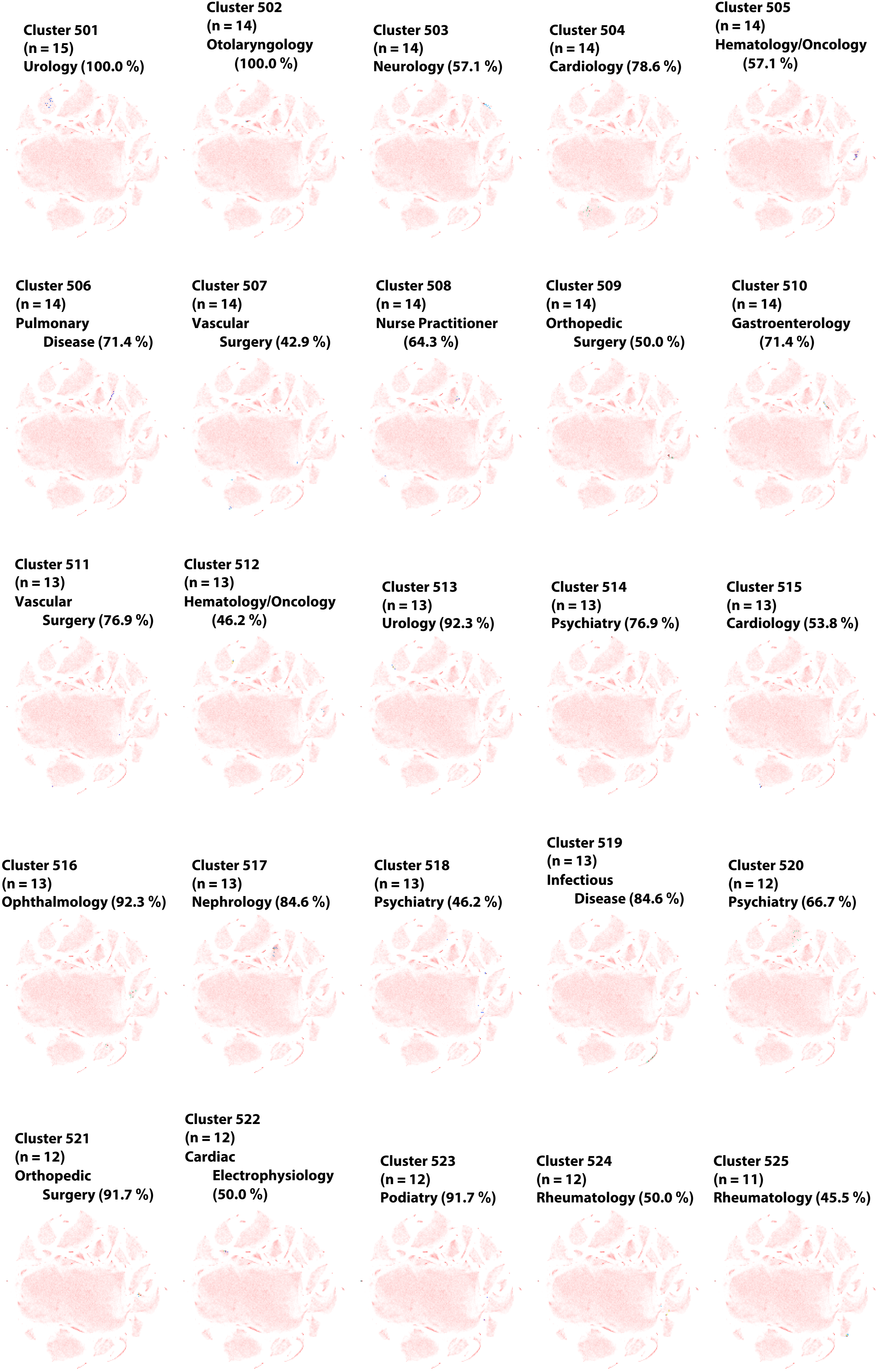

Cluster 501
(n = 15)
Urology (100.0 %)
Cluster 502
(n = 14)
Otolaryngology (100.0 %)
Cluster 503
(n = 14)
Neurology (57.1 %)
Cluster 504
(n = 14)
Cardiology (78.6 %)
Cluster 505
(n = 14)
Hematology/Oncology (57.1 %)
Cluster 506
(n = 14)
Pulmonary Disease (71.4 %)
Cluster 507
(n = 14)
Vascular Surgery (42.9 %)
Cluster 508
(n = 14)
Nurse Practitioner (64.3 %)
Cluster 509
(n = 14)
Orthopedic Surgery (50.0 %)
Cluster 510
(n = 14)
Gastroenterology (71.4 %)
Cluster 511
(n = 13)
Vascular Surgery (76.9 %)
Cluster 512
(n = 13)
Hematology/Oncology (46.2 %)
Cluster 513
(n = 13)
Urology (92.3 %)
Cluster 514
(n = 13)
Psychiatry (76.9 %)
Cluster 515
(n = 13)
Cardiology (53.8 %)
Cluster 516
(n = 13)
Ophthalmology (92.3 %)
Cluster 517
(n = 13)
Nephrology (84.6 %)
Cluster 518
(n = 13)
Psychiatry (46.2 %)
Cluster 519
(n = 13)
Infectious Disease (84.6 %)
Cluster 520
(n = 12)
Psychiatry (66.7 %)
Cluster 521
(n = 12)
Orthopedic Surgery (91.7 %)
Cluster 522
(n = 12)
Cardiac Electrophysiology (50.0 %)
Cluster 523
(n = 12)
Podiatry (91.7 %)
Cluster 524
(n = 12)
Rheumatology (50.0 %)
Cluster 525
(n = 11)
Rheumatology (45.5 %)

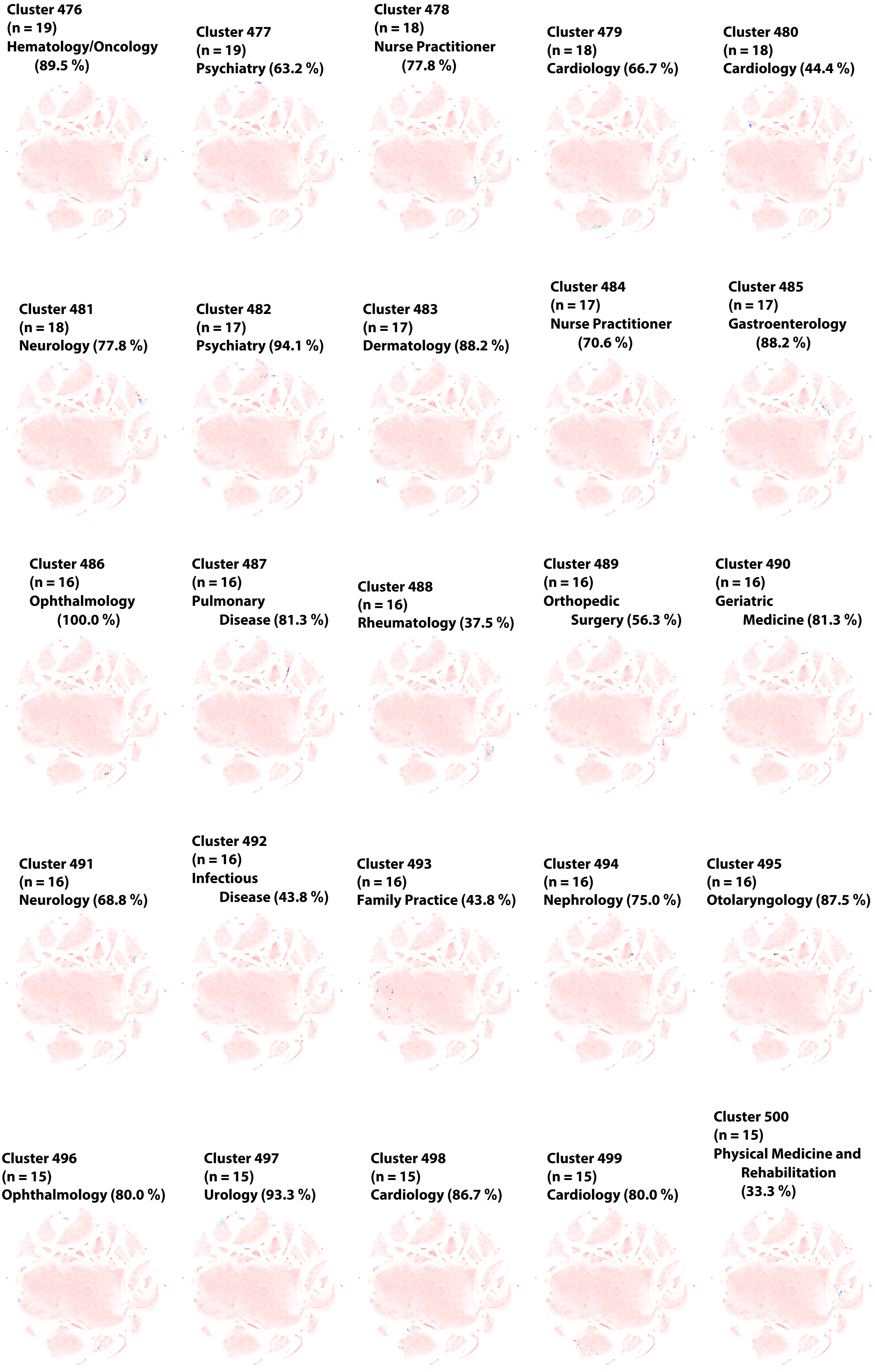
Cluster 476
(n = 19)
Hematology/Oncology (89.5 %)
Cluster 477
(n = 19)
Psychiatry (63.2 %)
Cluster 478
(n = 18)
Nurse Practitioner (77.8 %)
Cluster 479
(n = 18)
Cardiology (66.7 %)
Cluster 480
(n = 18)
Cardiology (44.4 %)
Cluster 481
(n = 18)
Neurology (77.8 %)
Cluster 482
(n = 17)
Psychiatry (94.1 %)
Cluster 483
(n = 17)
Dermatology (88.2 %)
Cluster 484
(n = 17)
Nurse Practitioner (70.6 %)
Cluster 485
(n = 17)
Gastroenterology (88.2 %)
Cluster 486
(n = 16)
Ophthalmology (100.0 %)
Cluster 487
(n = 16)
Pulmonary Disease (81.3 %)
Cluster 488
(n = 16)
Rheumatology (37.5 %)
Cluster 489
(n = 16)
Orthopedic Surgery (56.3 %)
Cluster 490
(n = 16)
Geriatric Medicine (81.3 %)
Cluster 491
(n = 16)
Neurology (68.8 %)
Cluster 492
(n = 16)
Infectious Disease (43.8 %)
Cluster 493
(n = 16)
Family Practice (43.8 %)
Cluster 494
(n = 16)
Nephrology (75.0 %)
Cluster 495
(n = 16)
Otolaryngology (87.5 %)
Cluster 496
(n = 15)
Ophthalmology (80.0 %)
Cluster 497
(n = 15)
Urology (93.3 %)
Cluster 498
(n = 15)
Cardiology (86.7 %)
Cluster 499
(n = 15)
Cardiology (80.0 %)
Cluster 500
(n = 15)
Physical Medicine and Rehabilitation (33.3 %)

Cluster 451
(n = 25)
Nurse Practitioner (88.0 %)

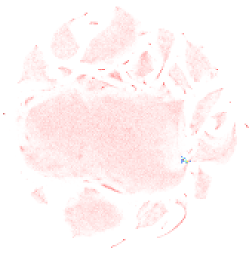

Cluster 452
(n = 25)
Nurse Practitioner (44.0 %)

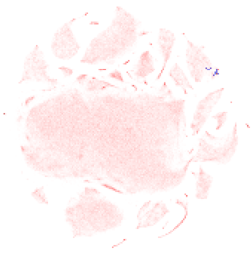

Cluster 453
(n = 25)
Infectious Disease (88.0 %)

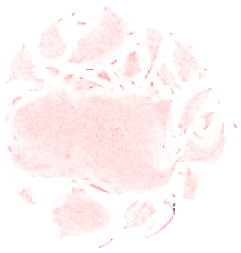

Cluster 454
(n = 24)
Urology (87.5 %)

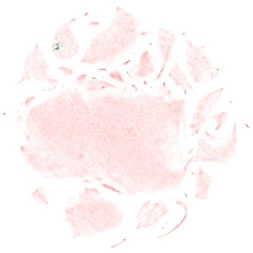

Cluster 455
(n = 24)
Neurology (100.0 %)

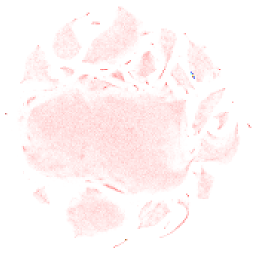

Cluster 456
(n = 24)
Psychiatry (87.5 %)

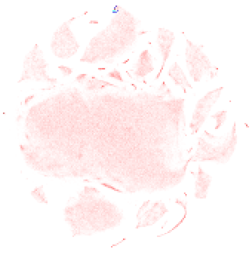

Cluster 457
(n = 23)
Nurse Practitioner (34.8 %)

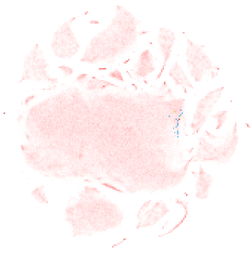

Cluster 458
(n = 23)
Neurology (95.7 %)

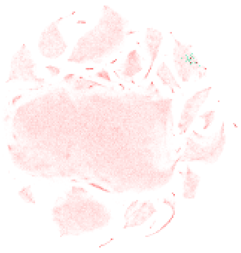

Cluster 459
(n = 22)
Psychiatry (54.5 %)

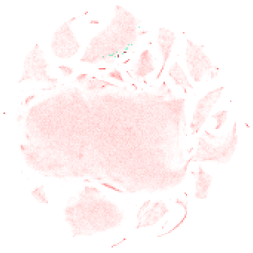

Cluster 460
(n = 22)
Family Practice (72.7 %)

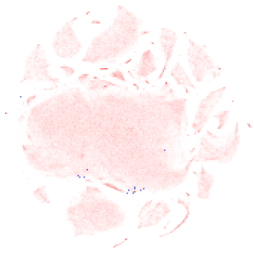

Cluster 461
(n = 22)
Pulmonary Disease (59.1 %)

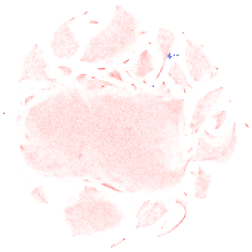

Cluster 462
(n = 22)
Hematology/Oncology (72.7 %)

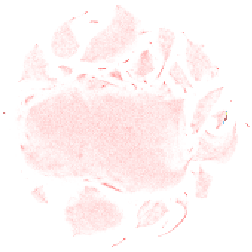

Cluster 463
(n = 22)
Psychiatry (54.5 %)

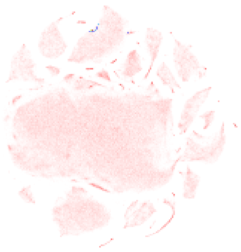

Cluster 464
(n = 22)
Cardiac Electrophysiology (45.5 %)

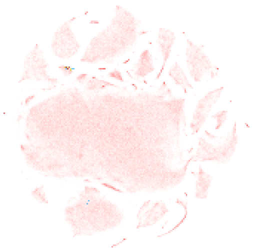

Cluster 465
(n = 22)
Psychiatry (31.8 %)

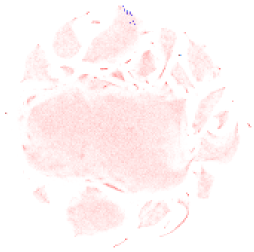

Cluster 466
(n = 21)
Neurology (81.0 %)

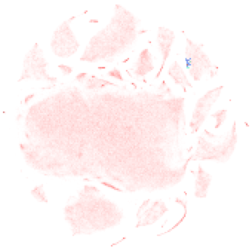

Cluster 467
(n = 21)
Obstetrics/Gynecology (81.0 %)

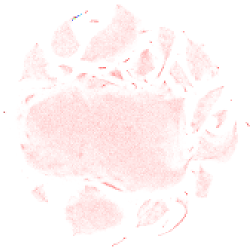

Cluster 468
(n = 20)
Urology (90.0 %)

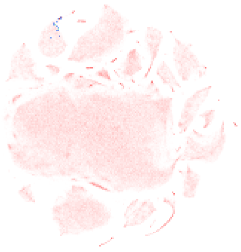

Cluster 469
(n = 19)
Hematology/Oncology (84.2 %)

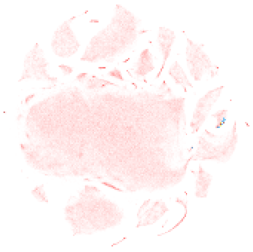

Cluster 470
(n = 19)
Internal Medicine (63.2 %)

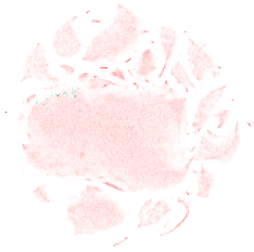

Cluster 471
(n = 19)
Psychiatry (63.2 %)

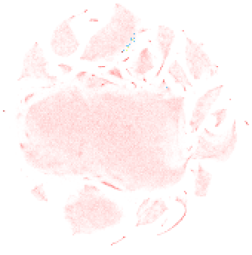

Cluster 472
(n = 19)
Endocrinology (84.2 %)

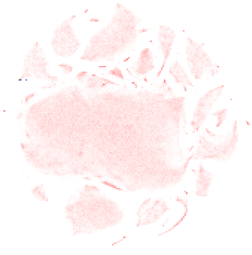

Cluster 473
(n = 19)
Family Practice (36.8 %)

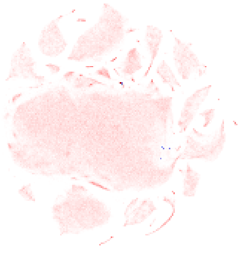

Cluster 474
(n = 19)
Neurology (68.4 %)

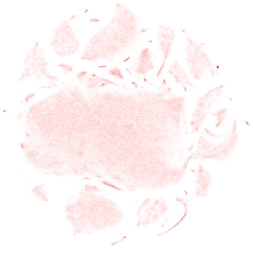

Cluster 475
(n = 19)
Nurse Practitioner (68.4 %)

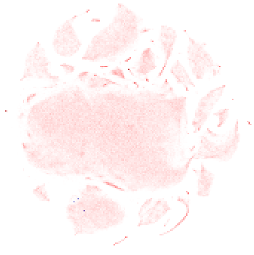

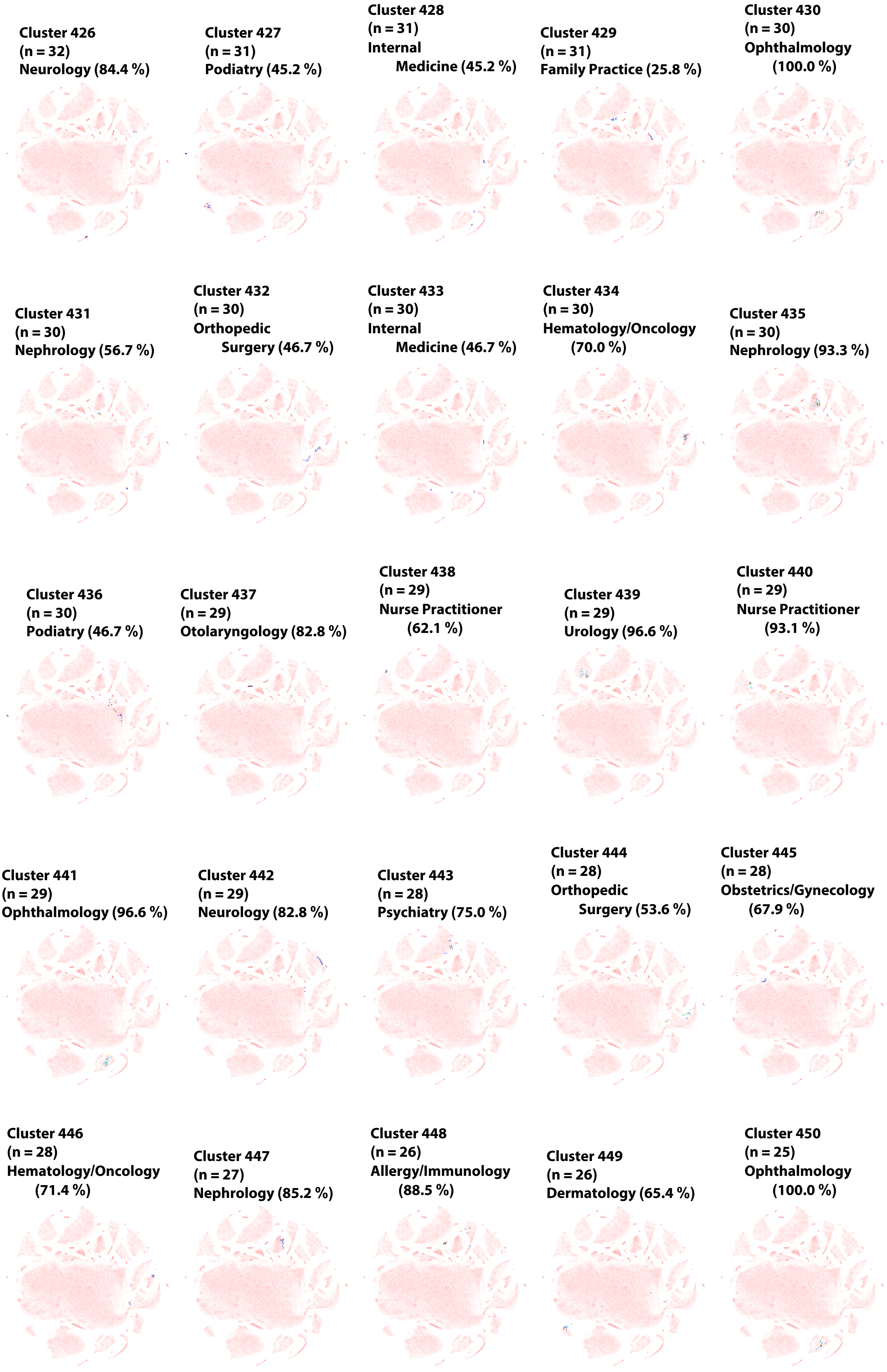
Cluster 426 (n = 32) Neurology (84.4 %)
Cluster 427 (n = 31) Podiatry (45.2 %)
Cluster 428 (n = 31) Internal Medicine (45.2 %)
Cluster 429 (n = 31) Family Practice (25.8 %)
Cluster 430 (n = 30) Ophthalmology (100.0 %)
Cluster 431 (n = 30) Nephrology (56.7 %)
Cluster 432 (n = 30) Orthopedic Surgery (46.7 %)
Cluster 433 (n = 30) Internal Medicine (46.7 %)
Cluster 434 (n = 30) Hematology/Oncology (70.0 %)
Cluster 435 (n = 30) Nephrology (93.3 %)
Cluster 436 (n = 30) Podiatry (46.7 %)
Cluster 437 (n = 29) Otolaryngology (82.8 %)
Cluster 438 (n = 29) Nurse Practitioner (62.1 %)
Cluster 439 (n = 29) Urology (96.6 %)
Cluster 440 (n = 29) Nurse Practitioner (93.1 %)
Cluster 441 (n = 29) Ophthalmology (96.6 %)
Cluster 442 (n = 29) Neurology (82.8 %)
Cluster 443 (n = 28) Psychiatry (75.0 %)
Cluster 444 (n = 28) Orthopedic Surgery (53.6 %)
Cluster 445 (n = 28) Obstetrics/Gynecology (67.9 %)
Cluster 446 (n = 28) Hematology/Oncology (71.4 %)
Cluster 447 (n = 27) Nephrology (85.2 %)
Cluster 448 (n = 26) Allergy/Immunology (88.5 %)
Cluster 449 (n = 26) Dermatology (65.4 %)
Cluster 450 (n = 25) Ophthalmology (100.0 %)

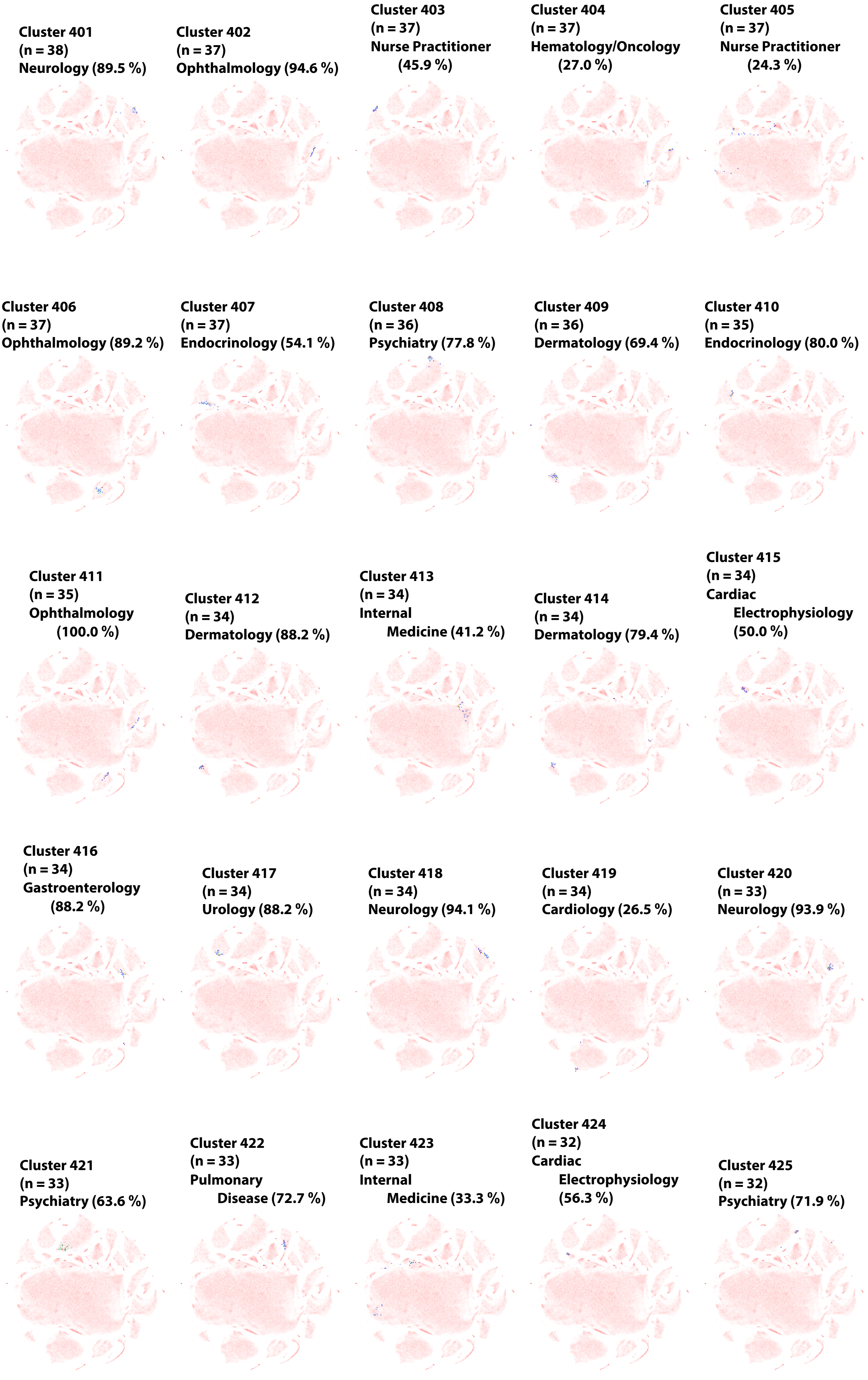
Cluster 401 (n = 38) Neurology (89.5 %)
Cluster 402 (n = 37) Ophthalmology (94.6 %)
Cluster 403 (n = 37) Nurse Practitioner (45.9 %)
Cluster 404 (n = 37) Hematology/Oncology (27.0 %)
Cluster 405 (n = 37) Nurse Practitioner (24.3 %)
Cluster 406 (n = 37) Ophthalmology (89.2 %)
Cluster 407 (n = 37) Endocrinology (54.1 %)
Cluster 408 (n = 36) Psychiatry (77.8 %)
Cluster 409 (n = 36) Dermatology (69.4 %)
Cluster 410 (n = 35) Endocrinology (80.0 %)
Cluster 411 (n = 35) Ophthalmology (100.0 %)
Cluster 412 (n = 34) Dermatology (88.2 %)
Cluster 413 (n = 34) Internal Medicine (41.2 %)
Cluster 414 (n = 34) Dermatology (79.4 %)
Cluster 415 (n = 34) Cardiac Electrophysiology (50.0 %)
Cluster 416 (n = 34) Gastroenterology (88.2 %)
Cluster 417 (n = 34) Urology (88.2 %)
Cluster 418 (n = 34) Neurology (94.1 %)
Cluster 419 (n = 34) Cardiology (26.5 %)
Cluster 420 (n = 33) Neurology (93.9 %)
Cluster 421 (n = 33) Psychiatry (63.6 %)
Cluster 422 (n = 33) Pulmonary Disease (72.7 %)
Cluster 423 (n = 33) Internal Medicine (33.3 %)
Cluster 424 (n = 32) Cardiac Electrophysiology (56.3 %)
Cluster 425 (n = 32) Psychiatry (71.9 %)

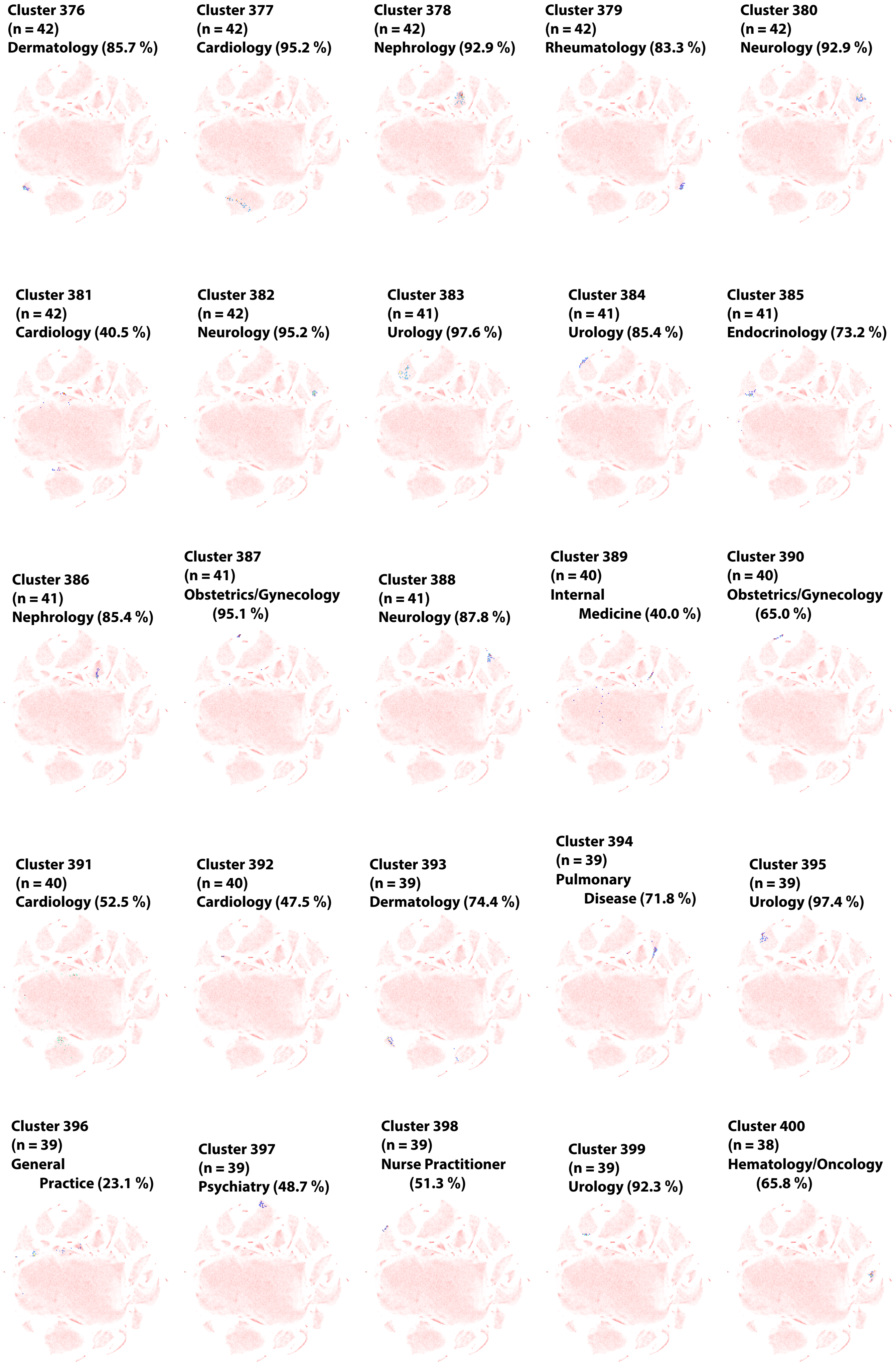
Cluster 376
(n = 42)
Dermatology (85.7 %)
Cluster 377
(n = 42)
Cardiology (95.2 %)
Cluster 378
(n = 42)
Nephrology (92.9 %)
Cluster 379
(n = 42)
Rheumatology (83.3 %)
Cluster 380
(n = 42)
Neurology (92.9 %)
Cluster 381
(n = 42)
Cardiology (40.5 %)
Cluster 382
(n = 42)
Neurology (95.2 %)
Cluster 383
(n = 41)
Urology (97.6 %)
Cluster 384
(n = 41)
Urology (85.4 %)
Cluster 385
(n = 41)
Endocrinology (73.2 %)
Cluster 386
(n = 41)
Nephrology (85.4 %)
Cluster 387
(n = 41)
Obstetrics/Gynecology
(95.1 %)
Cluster 388
(n = 41)
Neurology (87.8 %)
Cluster 389
(n = 40)
Internal
Medicine (40.0 %)
Cluster 390
(n = 40)
Obstetrics/Gynecology
(65.0 %)
Cluster 391
(n = 40)
Cardiology (52.5 %)
Cluster 392
(n = 40)
Cardiology (47.5 %)
Cluster 393
(n = 39)
Dermatology (74.4 %)
Cluster 394
(n = 39)
Pulmonary
Disease (71.8 %)
Cluster 395
(n = 39)
Urology (97.4 %)
Cluster 396
(n = 39)
General
Practice (23.1 %)
Cluster 397
(n = 39)
Psychiatry (48.7 %)
Cluster 398
(n = 39)
Nurse Practitioner
(51.3 %)
Cluster 399
(n = 39)
Urology (92.3 %)
Cluster 400
(n = 38)
Hematology/Oncology
(65.8 %)

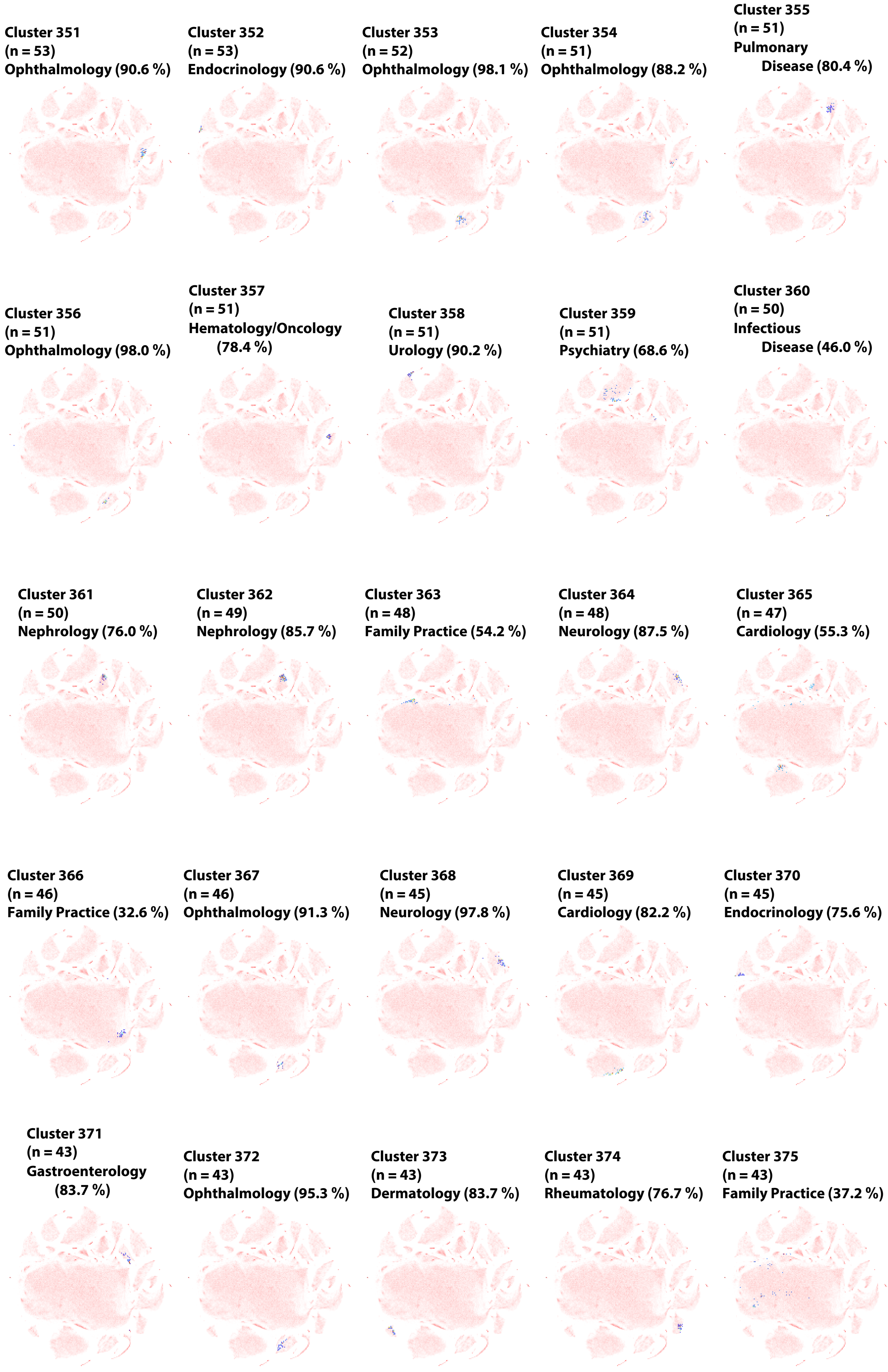
Cluster 351
(n = 53)
Ophthalmology (90.6 %)
Cluster 352
(n = 53)
Endocrinology (90.6 %)
Cluster 353
(n = 52)
Ophthalmology (98.1 %)
Cluster 354
(n = 51)
Ophthalmology (88.2 %)
Cluster 355
(n = 51)
Pulmonary Disease (80.4 %)
Cluster 356
(n = 51)
Ophthalmology (98.0 %)
Cluster 357
(n = 51)
Hematology/Oncology (78.4 %)
Cluster 358
(n = 51)
Urology (90.2 %)
Cluster 359
(n = 51)
Psychiatry (68.6 %)
Cluster 360
(n = 50)
Infectious Disease (46.0 %)
Cluster 361
(n = 50)
Nephrology (76.0 %)
Cluster 362
(n = 49)
Nephrology (85.7 %)
Cluster 363
(n = 48)
Family Practice (54.2 %)
Cluster 364
(n = 48)
Neurology (87.5 %)
Cluster 365
(n = 47)
Cardiology (55.3 %)
Cluster 366
(n = 46)
Family Practice (32.6 %)
Cluster 367
(n = 46)
Ophthalmology (91.3 %)
Cluster 368
(n = 45)
Neurology (97.8 %)
Cluster 369
(n = 45)
Cardiology (82.2 %)
Cluster 370
(n = 45)
Endocrinology (75.6 %)
Cluster 371
(n = 43)
Gastroenterology (83.7 %)
Cluster 372
(n = 43)
Ophthalmology (95.3 %)
Cluster 373
(n = 43)
Dermatology (83.7 %)
Cluster 374
(n = 43)
Rheumatology (76.7 %)
Cluster 375
(n = 43)
Family Practice (37.2 %)

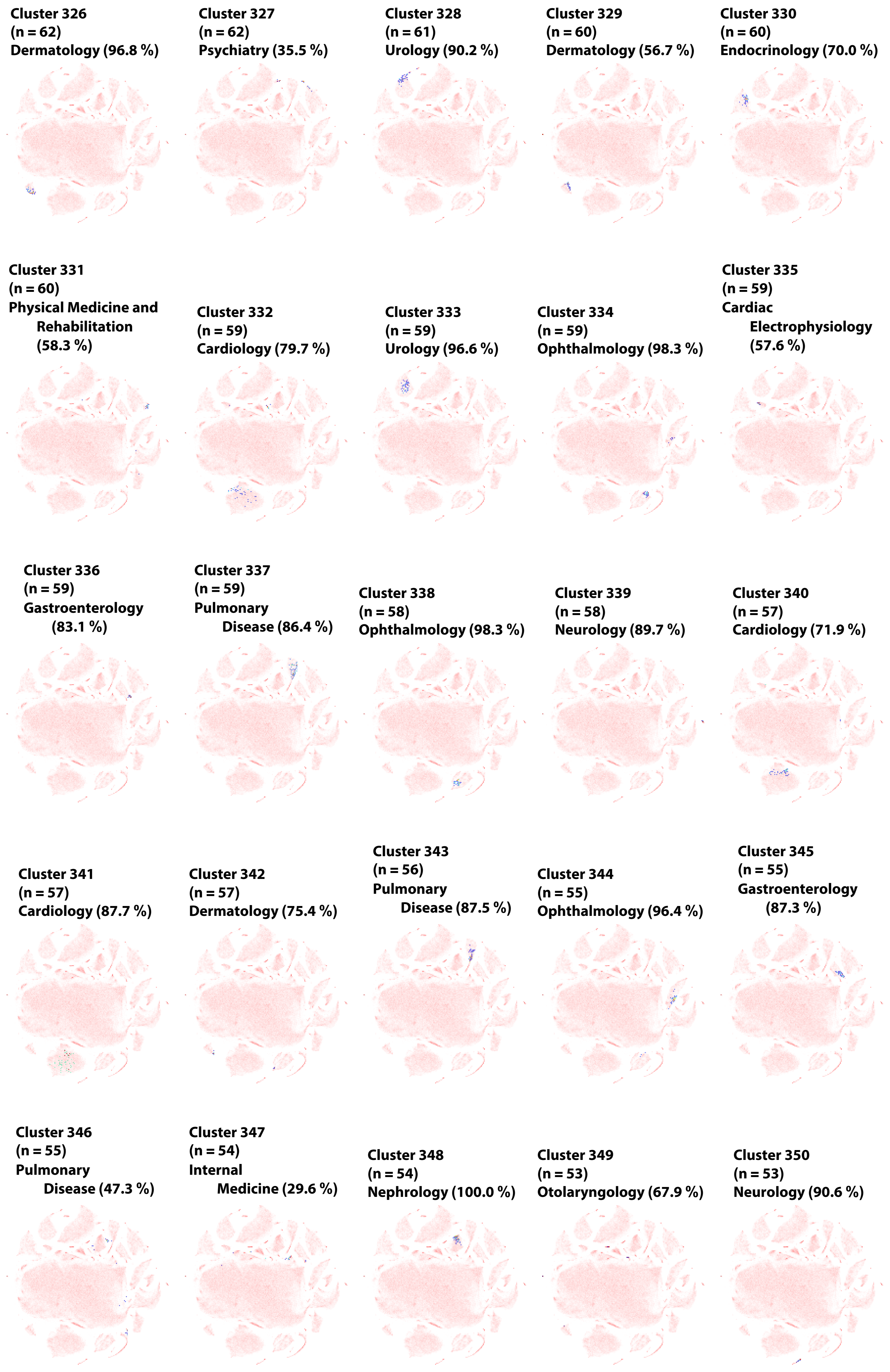
Cluster 326
(n = 62)
Dermatology (96.8 %)
Cluster 327
(n = 62)
Psychiatry (35.5 %)
Cluster 328
(n = 61)
Urology (90.2 %)
Cluster 329
(n = 60)
Dermatology (56.7 %)
Cluster 330
(n = 60)
Endocrinology (70.0 %)
Cluster 331
(n = 60)
Physical Medicine and Rehabilitation (58.3 %)
Cluster 332
(n = 59)
Cardiology (79.7 %)
Cluster 333
(n = 59)
Urology (96.6 %)
Cluster 334
(n = 59)
Ophthalmology (98.3 %)
Cluster 335
(n = 59)
Cardiac Electrophysiology (57.6 %)
Cluster 336
(n = 59)
Gastroenterology (83.1 %)
Cluster 337
(n = 59)
Pulmonary Disease (86.4 %)
Cluster 338
(n = 58)
Ophthalmology (98.3 %)
Cluster 339
(n = 58)
Neurology (89.7 %)
Cluster 340
(n = 57)
Cardiology (71.9 %)
Cluster 341
(n = 57)
Cardiology (87.7 %)
Cluster 342
(n = 57)
Dermatology (75.4 %)
Cluster 343
(n = 56)
Pulmonary Disease (87.5 %)
Cluster 344
(n = 55)
Ophthalmology (96.4 %)
Cluster 345
(n = 55)
Gastroenterology (87.3 %)
Cluster 346
(n = 55)
Pulmonary Disease (47.3 %)
Cluster 347
(n = 54)
Internal Medicine (29.6 %)
Cluster 348
(n = 54)
Nephrology (100.0 %)
Cluster 349
(n = 53)
Otolaryngology (67.9 %)
Cluster 350
(n = 53)
Neurology (90.6 %)

Cluster 301
(n = 74)
Gastroenterology
(83.8 %)

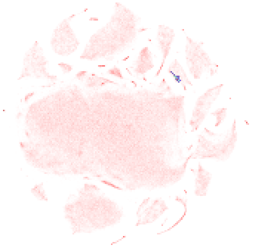

Cluster 302
(n = 73)
Physician
Assistant (26.0 %)

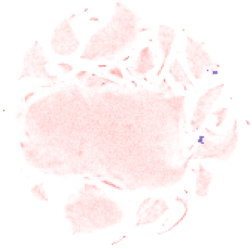

Cluster 303
(n = 73)
Ophthalmology (91.8 %)

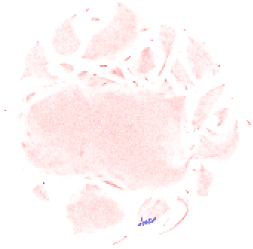

Cluster 304
(n = 73)
Ophthalmology (86.3 %)

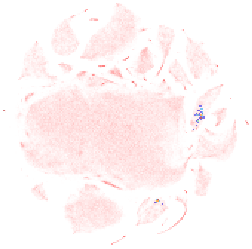

Cluster 305
(n = 72)
Family Practice (31.9 %)

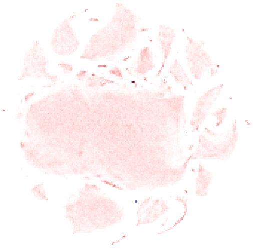

Cluster 306
(n = 72)
Ophthalmology (79.2 %)

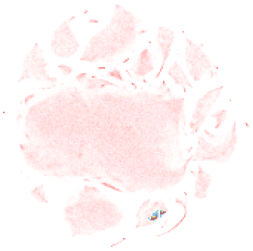

Cluster 307
(n = 71)
Family Practice (38.0 %)

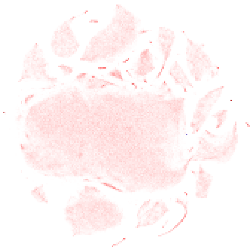

Cluster 308
(n = 71)
Nephrology (88.7 %)

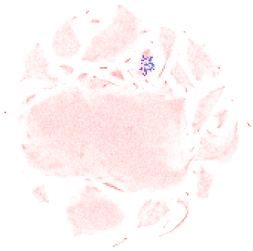

Cluster 309
(n = 70)
Endocrinology (80.0 %)

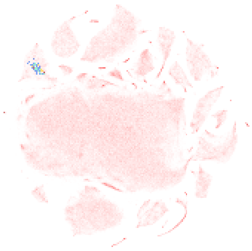

Cluster 310
(n = 70)
Nurse Practitioner
(51.4 %)

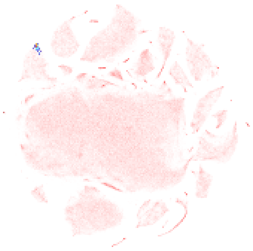

Cluster 311
(n = 69)
Ophthalmology
(100.0 %)

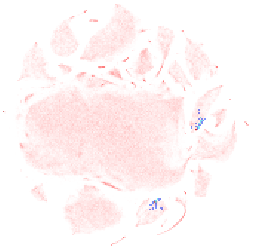

Cluster 312
(n = 68)
Rheumatology (64.7 %)

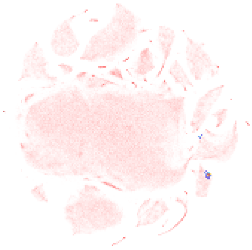

Cluster 313
(n = 68)
Cardiology (64.7 %)

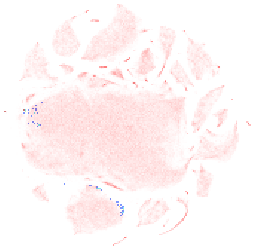

Cluster 314
(n = 68)
Emergency
Medicine (33.8 %)

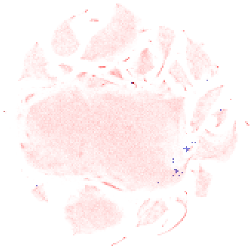

Cluster 315
(n = 68)
Gastroenterology
(70.6 %)

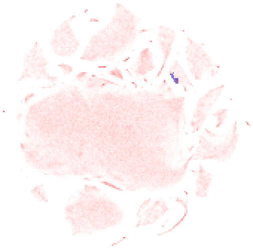

Cluster 316
(n = 67)
Family Practice (25.4 %)

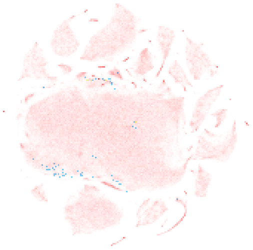

Cluster 317
(n = 67)
Obstetrics/Gynecology
(97.0 %)

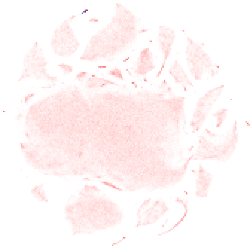

Cluster 318
(n = 66)
Cardiology (93.9 %)

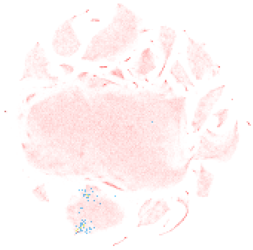

Cluster 319
(n = 66)
Neurology (97.0 %)

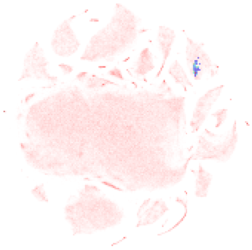

Cluster 320
(n = 65)
Rheumatology (83.1 %)

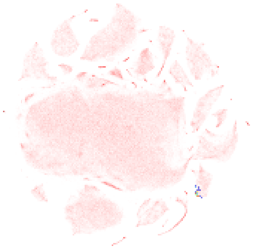

Cluster 321
(n = 65)
Neurology (90.8 %)

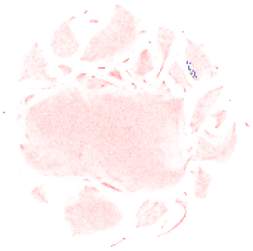

Cluster 322
(n = 64)
Neurology (25.0 %)

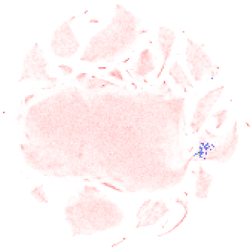

Cluster 323
(n = 64)
Physical Medicine and
Rehabilitation
(29.7 %)

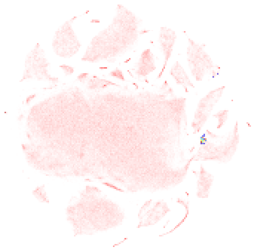

Cluster 324
(n = 62)
Urology (80.6 %)

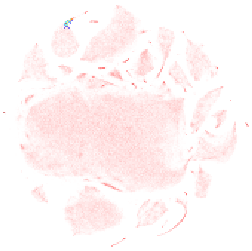

Cluster 325
(n = 62)
Pulmonary
Disease (54.8 %)

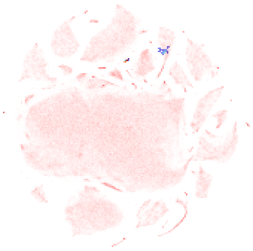

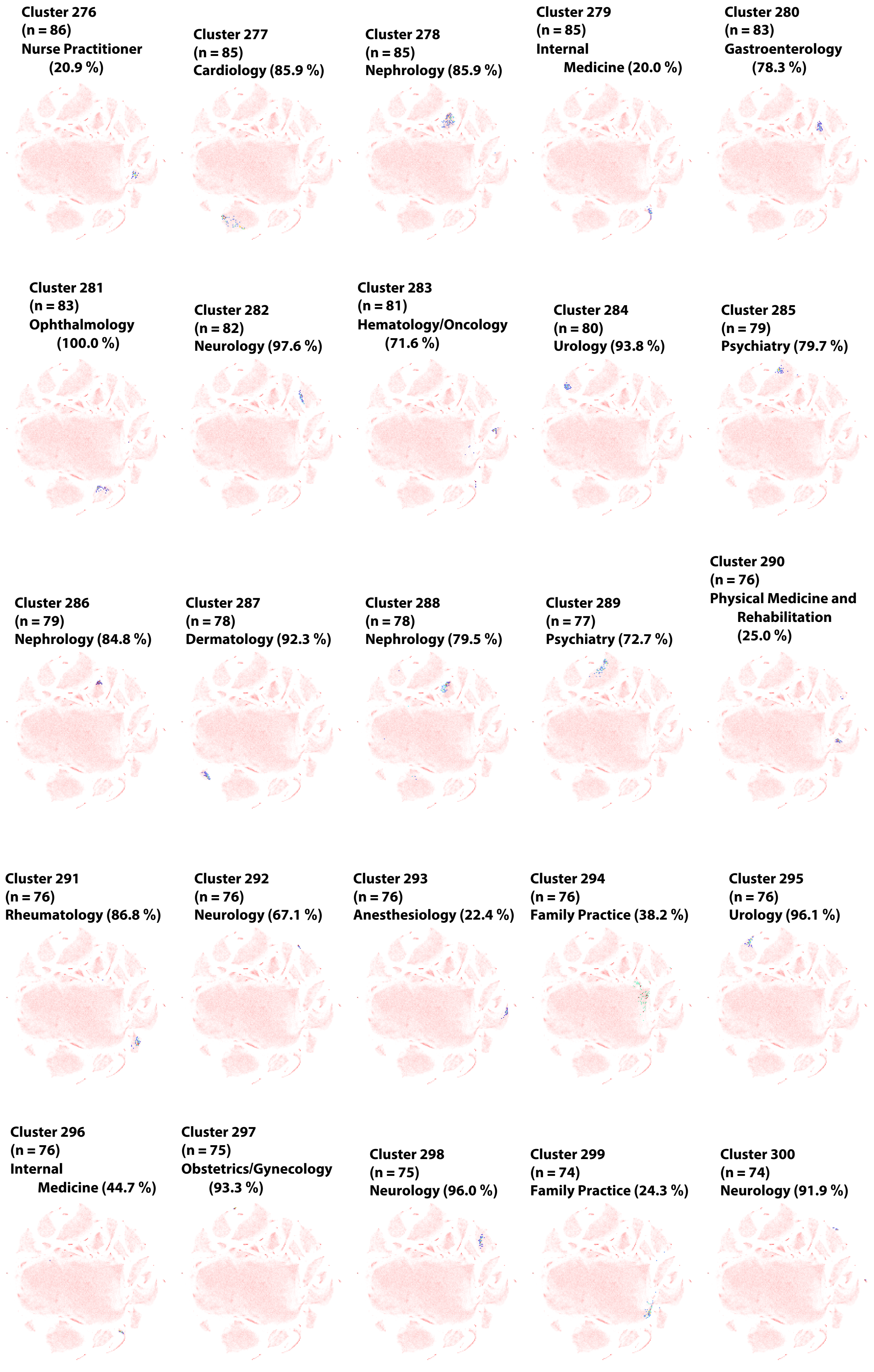
Cluster 276
(n = 86)
Nurse Practitioner (20.9 %)
Cluster 277
(n = 85)
Cardiology (85.9 %)
Cluster 278
(n = 85)
Nephrology (85.9 %)
Cluster 279
(n = 85)
Internal Medicine (20.0 %)
Cluster 280
(n = 83)
Gastroenterology (78.3 %)
Cluster 281
(n = 83)
Ophthalmology (100.0 %)
Cluster 282
(n = 82)
Neurology (97.6 %)
Cluster 283
(n = 81)
Hematology/Oncology (71.6 %)
Cluster 284
(n = 80)
Urology (93.8 %)
Cluster 285
(n = 79)
Psychiatry (79.7 %)
Cluster 286
(n = 79)
Nephrology (84.8 %)
Cluster 287
(n = 78)
Dermatology (92.3 %)
Cluster 288
(n = 78)
Nephrology (79.5 %)
Cluster 289
(n = 77)
Psychiatry (72.7 %)
Cluster 290
(n = 76)
Physical Medicine and Rehabilitation (25.0 %)
Cluster 291
(n = 76)
Rheumatology (86.8 %)
Cluster 292
(n = 76)
Neurology (67.1 %)
Cluster 293
(n = 76)
Anesthesiology (22.4 %)
Cluster 294
(n = 76)
Family Practice (38.2 %)
Cluster 295
(n = 76)
Urology (96.1 %)
Cluster 296
(n = 76)
Internal Medicine (44.7 %)
Cluster 297
(n = 75)
Obstetrics/Gynecology (93.3 %)
Cluster 298
(n = 75)
Neurology (96.0 %)
Cluster 299
(n = 74)
Family Practice (24.3 %)
Cluster 300
(n = 74)
Neurology (91.9 %)

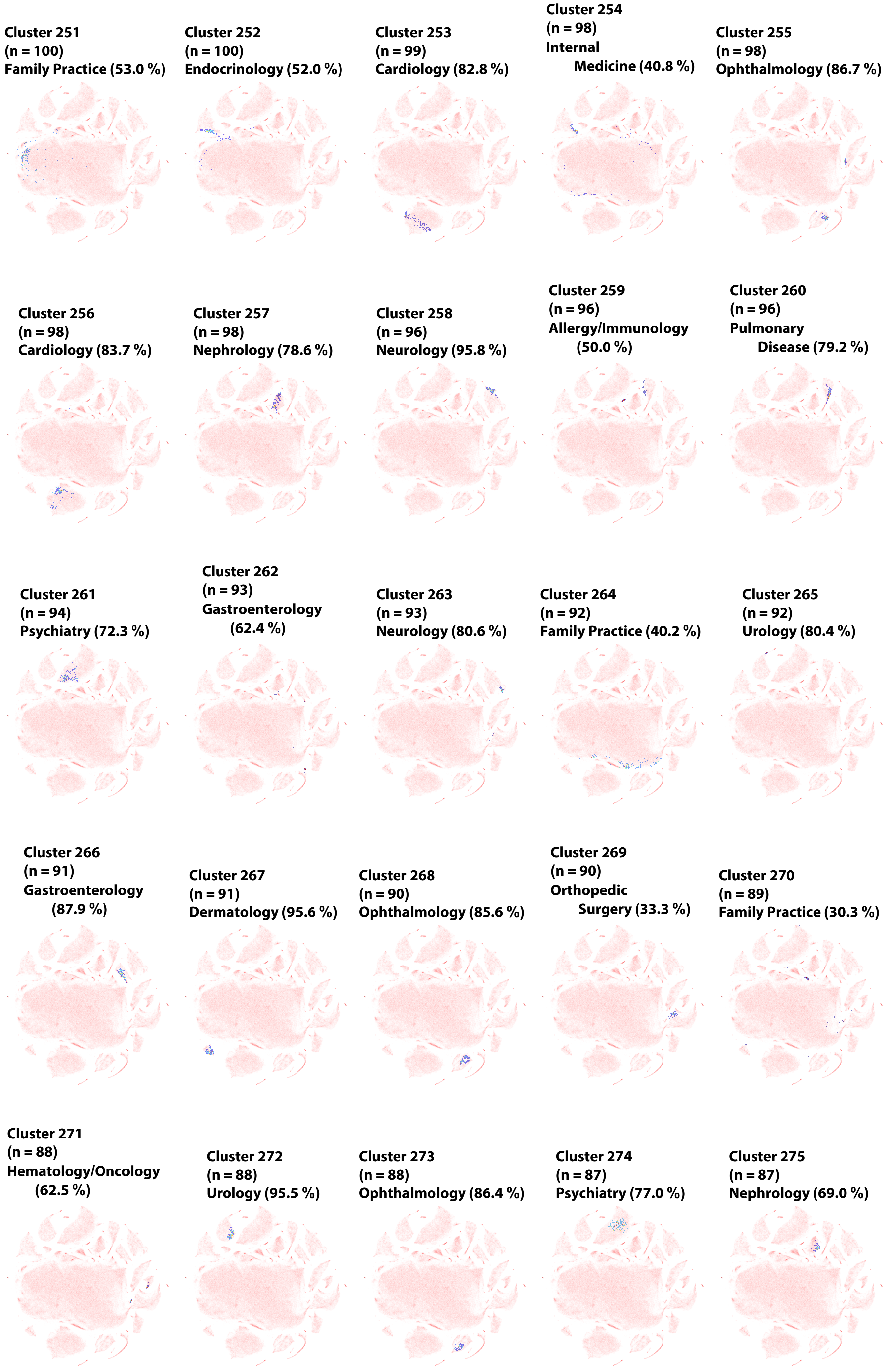
Cluster 251
(n = 100)
Family Practice (53.0 %)
Cluster 252
(n = 100)
Endocrinology (52.0 %)
Cluster 253
(n = 99)
Cardiology (82.8 %)
Cluster 254
(n = 98)
Internal Medicine (40.8 %)
Cluster 255
(n = 98)
Ophthalmology (86.7 %)
Cluster 256
(n = 98)
Cardiology (83.7 %)
Cluster 257
(n = 98)
Nephrology (78.6 %)
Cluster 258
(n = 96)
Neurology (95.8 %)
Cluster 259
(n = 96)
Allergy/Immunology (50.0 %)
Cluster 260
(n = 96)
Pulmonary Disease (79.2 %)
Cluster 261
(n = 94)
Psychiatry (72.3 %)
Cluster 262
(n = 93)
Gastroenterology (62.4 %)
Cluster 263
(n = 93)
Neurology (80.6 %)
Cluster 264
(n = 92)
Family Practice (40.2 %)
Cluster 265
(n = 92)
Urology (80.4 %)
Cluster 266
(n = 91)
Gastroenterology (87.9 %)
Cluster 267
(n = 91)
Dermatology (95.6 %)
Cluster 268
(n = 90)
Ophthalmology (85.6 %)
Cluster 269
(n = 90)
Orthopedic Surgery (33.3 %)
Cluster 270
(n = 89)
Family Practice (30.3 %)
Cluster 271
(n = 88)
Hematology/Oncology (62.5 %)
Cluster 272
(n = 88)
Urology (95.5 %)
Cluster 273
(n = 88)
Ophthalmology (86.4 %)
Cluster 274
(n = 87)
Psychiatry (77.0 %)
Cluster 275
(n = 87)
Nephrology (69.0 %)

Cluster 226
(n = 114)
Hematology/Oncology (70.2 %)

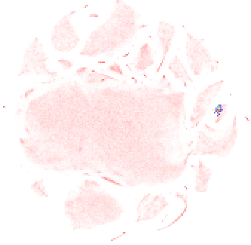

Cluster 227
(n = 113)
Psychiatry (72.6 %)

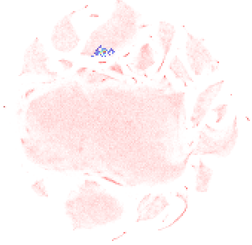

Cluster 228
(n = 113)
Pulmonary Disease (56.6 %)

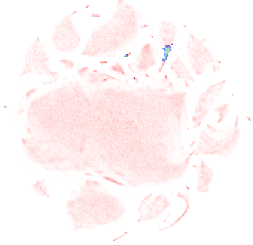

Cluster 229
(n = 112)
Nephrology (88.4 %)

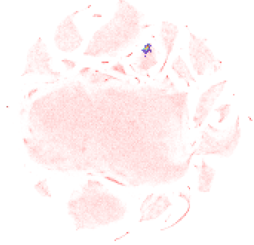

Cluster 230
(n = 111)
Endocrinology (65.8 %)

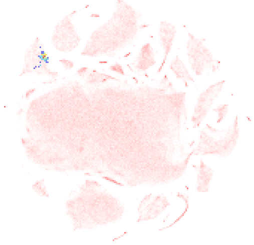

Cluster 231
(n = 110)
Hematology/Oncology (72.7 %)

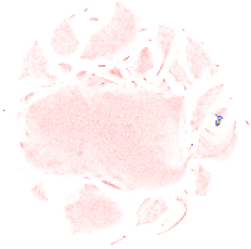

Cluster 232
(n = 109)
Orthopedic Surgery (78.0 %)

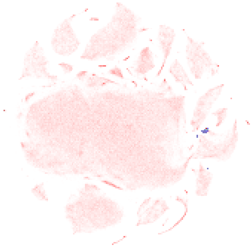

Cluster 233
(n = 109)
Nurse Practitioner (43.1 %)

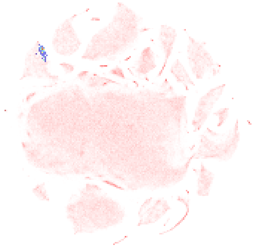

Cluster 234
(n = 109)
Urology (93.6 %)

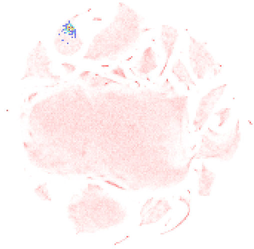

Cluster 235
(n = 109)
Neurology (93.6 %)

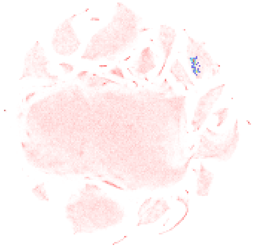

Cluster 236
(n = 108)
Ophthalmology (96.3 %)

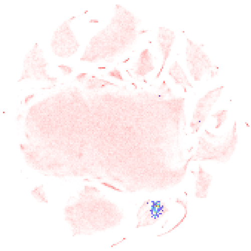

Cluster 237
(n = 108)
Rheumatology (89.8 %)

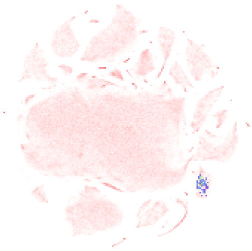

Cluster 238
(n = 107)
Urology (92.5 %)

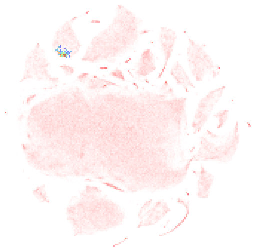

Cluster 239
(n = 107)
Nurse Practitioner (29.9 %)

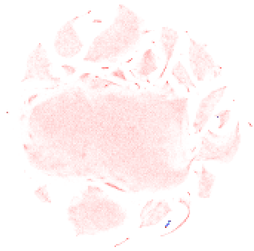

Cluster 240
(n = 107)
Otolaryngology (84.1 %)

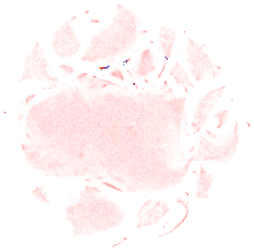

Cluster 241
(n = 107)
Physical Medicine and Rehabilitation (19.6 %)

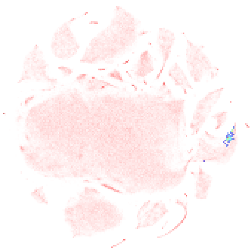

Cluster 242
(n = 106)
Rheumatology (84.9 %)

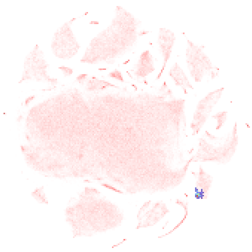

Cluster 243
(n = 104)
Rheumatology (86.5 %)

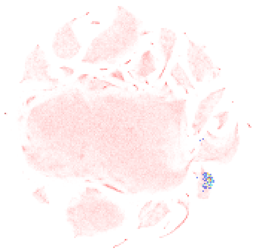

Cluster 244
(n = 104)
Gastroenterology (87.5 %)

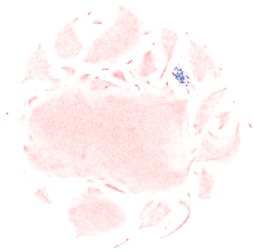

Cluster 245
(n = 104)
Hematology/Oncology (61.5 %)

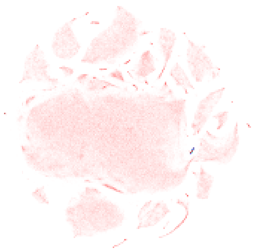

Cluster 246
(n = 104)
Gastroenterology (89.4 %)

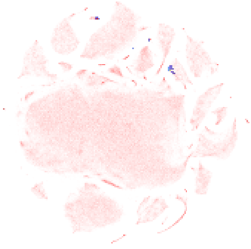

Cluster 247
(n = 102)
Neurology (65.7 %)

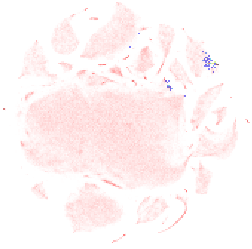

Cluster 248
(n = 101)
Ophthalmology (97.0 %)

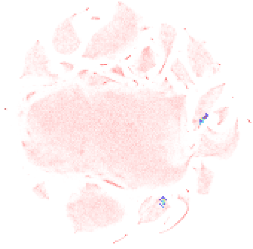

Cluster 249
(n = 101)
Internal Medicine (35.6 %)

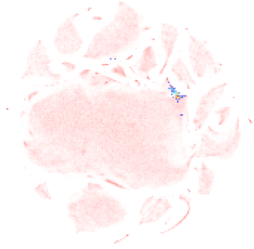

Cluster 250
(n = 100)
Family Practice (23.0 %)

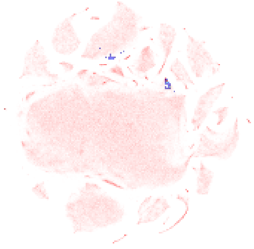

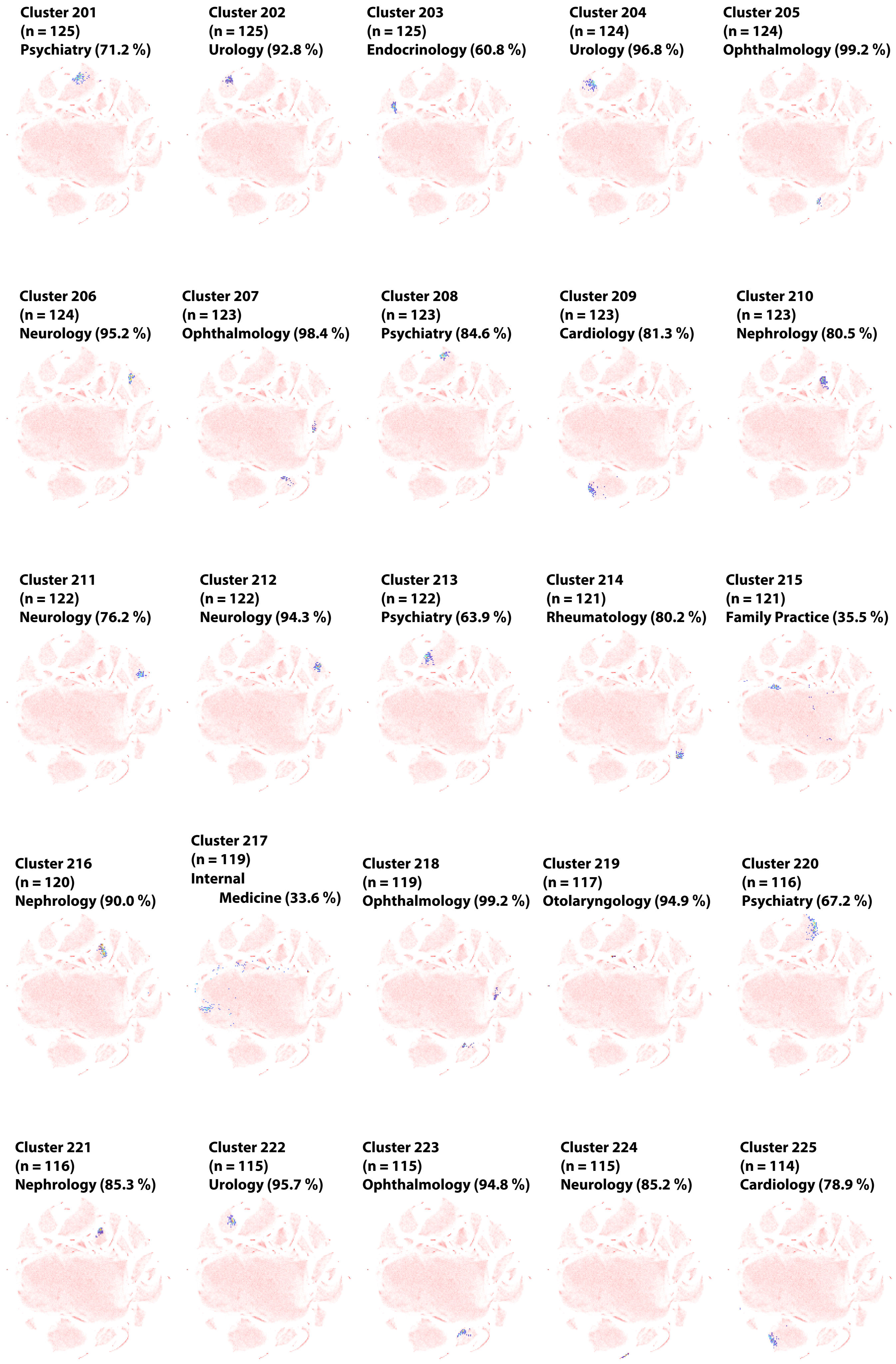
Cluster 201
(n = 125)
Psychiatry (71.2 %)
Cluster 202
(n = 125)
Urology (92.8 %)
Cluster 203
(n = 125)
Endocrinology (60.8 %)
Cluster 204
(n = 124)
Urology (96.8 %)
Cluster 205
(n = 124)
Ophthalmology (99.2 %)
Cluster 206
(n = 124)
Neurology (95.2 %)
Cluster 207
(n = 123)
Ophthalmology (98.4 %)
Cluster 208
(n = 123)
Psychiatry (84.6 %)
Cluster 209
(n = 123)
Cardiology (81.3 %)
Cluster 210
(n = 123)
Nephrology (80.5 %)
Cluster 211
(n = 122)
Neurology (76.2 %)
Cluster 212
(n = 122)
Neurology (94.3 %)
Cluster 213
(n = 122)
Psychiatry (63.9 %)
Cluster 214
(n = 121)
Rheumatology (80.2 %)
Cluster 215
(n = 121)
Family Practice (35.5 %)
Cluster 216
(n = 120)
Nephrology (90.0 %)
Cluster 217
(n = 119)
Internal
Medicine (33.6 %)
Cluster 218
(n = 119)
Ophthalmology (99.2 %)
Cluster 219
(n = 117)
Otolaryngology (94.9 %)
Cluster 220
(n = 116)
Psychiatry (67.2 %)
Cluster 221
(n = 116)
Nephrology (85.3 %)
Cluster 222
(n = 115)
Urology (95.7 %)
Cluster 223
(n = 115)
Ophthalmology (94.8 %)
Cluster 224
(n = 115)
Neurology (85.2 %)
Cluster 225
(n = 114)
Cardiology (78.9 %)

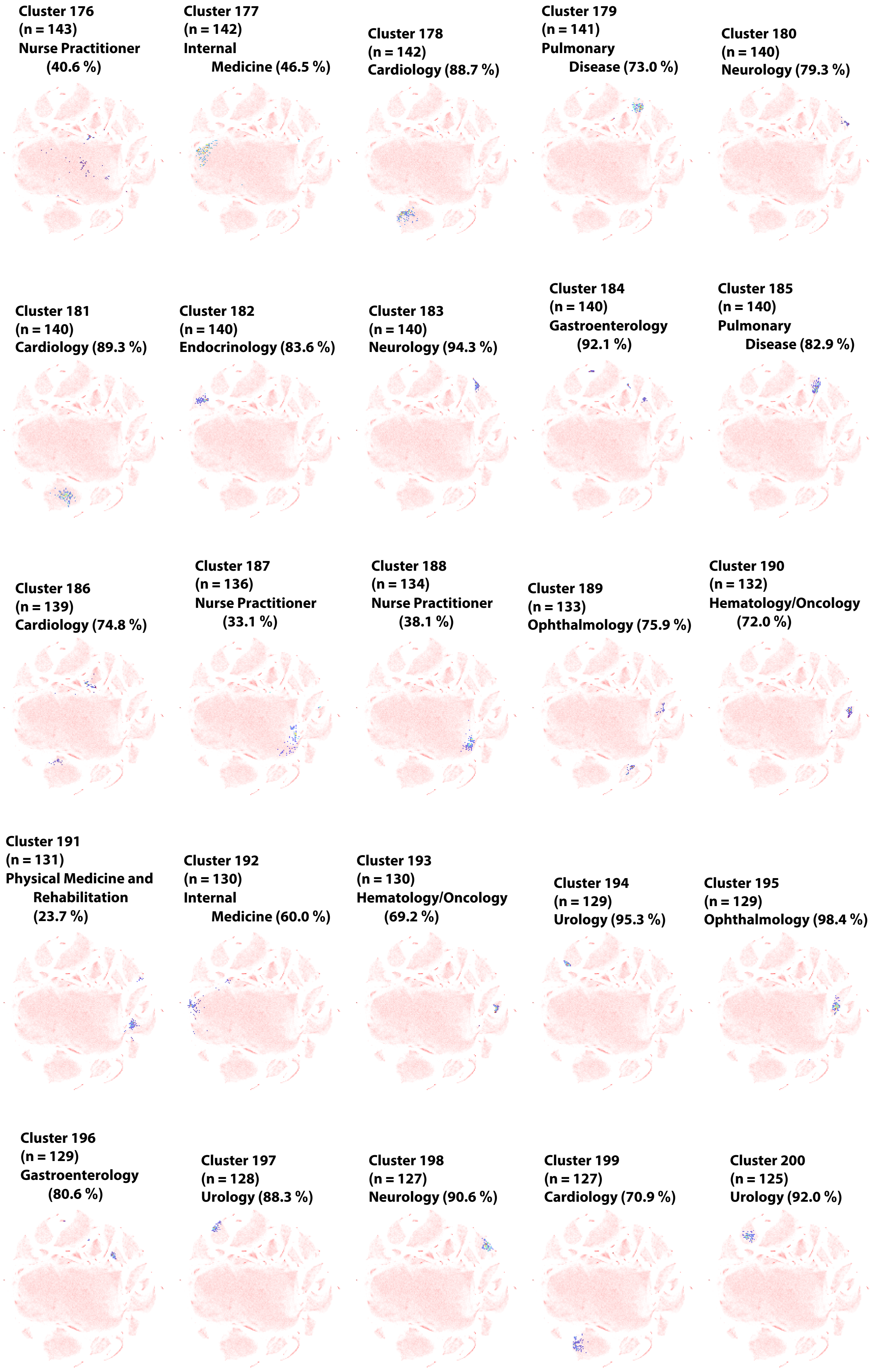
Cluster 176
(n = 143)
Nurse Practitioner
(40.6 %)
Cluster 177
(n = 142)
Internal
Medicine (46.5 %)
Cluster 178
(n = 142)
Cardiology (88.7 %)
Cluster 179
(n = 141)
Pulmonary
Disease (73.0 %)
Cluster 180
(n = 140)
Neurology (79.3 %)
Cluster 181
(n = 140)
Cardiology (89.3 %)
Cluster 182
(n = 140)
Endocrinology (83.6 %)
Cluster 183
(n = 140)
Neurology (94.3 %)
Cluster 184
(n = 140)
Gastroenterology
(92.1 %)
Cluster 185
(n = 140)
Pulmonary
Disease (82.9 %)
Cluster 186
(n = 139)
Cardiology (74.8 %)
Cluster 187
(n = 136)
Nurse Practitioner
(33.1 %)
Cluster 188
(n = 134)
Nurse Practitioner
(38.1 %)
Cluster 189
(n = 133)
Ophthalmology (75.9 %)
Cluster 190
(n = 132)
Hematology/Oncology
(72.0 %)
Cluster 191
(n = 131)
Physical Medicine and
Rehabilitation
(23.7 %)
Cluster 192
(n = 130)
Internal
Medicine (60.0 %)
Cluster 193
(n = 130)
Hematology/Oncology
(69.2 %)
Cluster 194
(n = 129)
Urology (95.3 %)
Cluster 195
(n = 129)
Ophthalmology (98.4 %)
Cluster 196
(n = 129)
Gastroenterology
(80.6 %)
Cluster 197
(n = 128)
Urology (88.3 %)
Cluster 198
(n = 127)
Neurology (90.6 %)
Cluster 199
(n = 127)
Cardiology (70.9 %)
Cluster 200
(n = 125)
Urology (92.0 %)

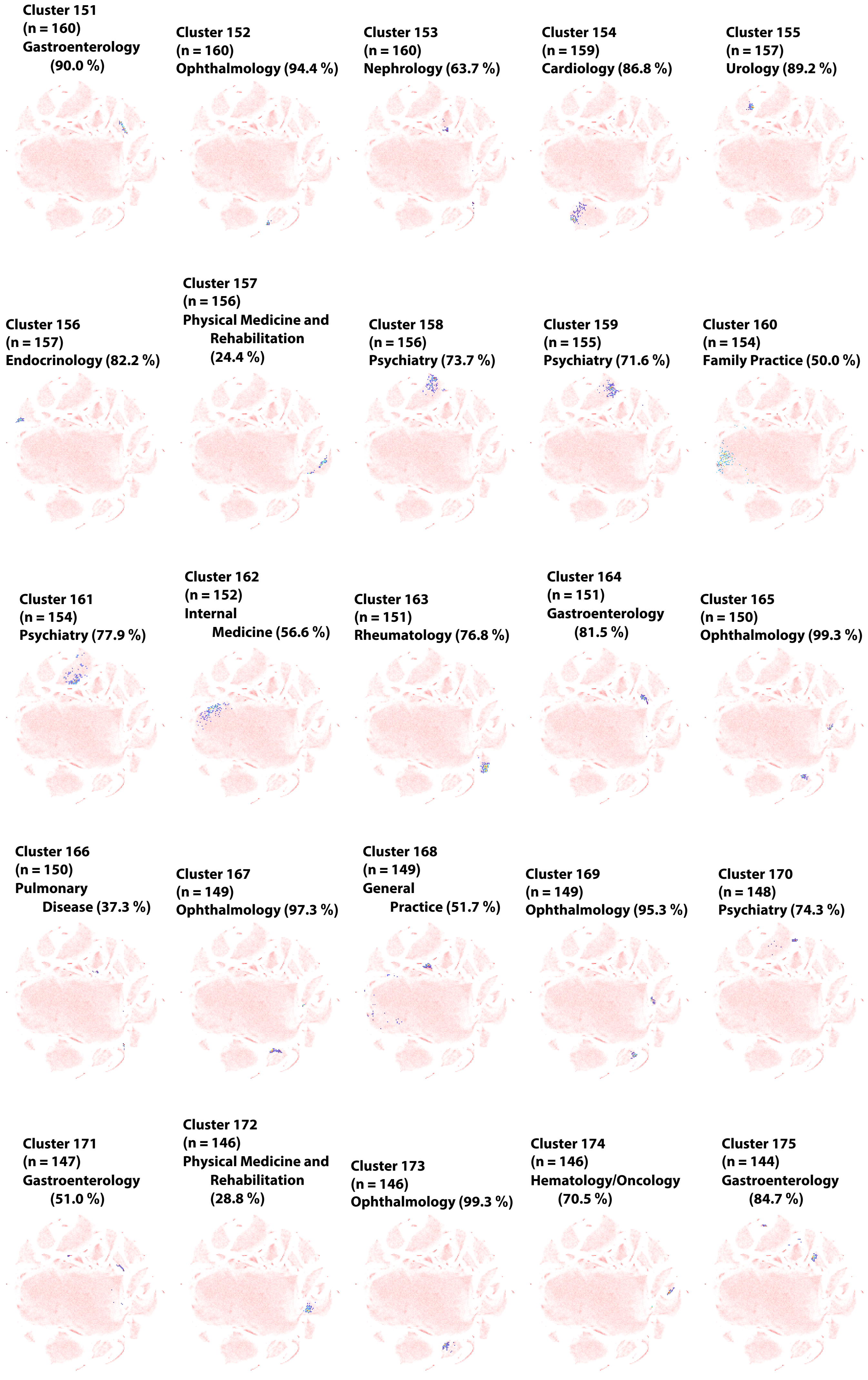
Cluster 151
(n = 160)
Gastroenterology
(90.0 %)
Cluster 152
(n = 160)
Ophthalmology (94.4 %)
Cluster 153
(n = 160)
Nephrology (63.7 %)
Cluster 154
(n = 159)
Cardiology (86.8 %)
Cluster 155
(n = 157)
Urology (89.2 %)
Cluster 156
(n = 157)
Endocrinology (82.2 %)
Cluster 157
(n = 156)
Physical Medicine and
Rehabilitation
(24.4 %)
Cluster 158
(n = 156)
Psychiatry (73.7 %)
Cluster 159
(n = 155)
Psychiatry (71.6 %)
Cluster 160
(n = 154)
Family Practice (50.0 %)
Cluster 161
(n = 154)
Psychiatry (77.9 %)
Cluster 162
(n = 152)
Internal
Medicine (56.6 %)
Cluster 163
(n = 151)
Rheumatology (76.8 %)
Cluster 164
(n = 151)
Gastroenterology
(81.5 %)
Cluster 165
(n = 150)
Ophthalmology (99.3 %)
Cluster 166
(n = 150)
Pulmonary
Disease (37.3 %)
Cluster 167
(n = 149)
Ophthalmology (97.3 %)
Cluster 168
(n = 149)
General
Practice (51.7 %)
Cluster 169
(n = 149)
Ophthalmology (95.3 %)
Cluster 170
(n = 148)
Psychiatry (74.3 %)
Cluster 171
(n = 147)
Gastroenterology
(51.0 %)
Cluster 172
(n = 146)
Physical Medicine and
Rehabilitation
(28.8 %)
Cluster 173
(n = 146)
Ophthalmology (99.3 %)
Cluster 174
(n = 146)
Hematology/Oncology
(70.5 %)
Cluster 175
(n = 144)
Gastroenterology
(84.7 %)

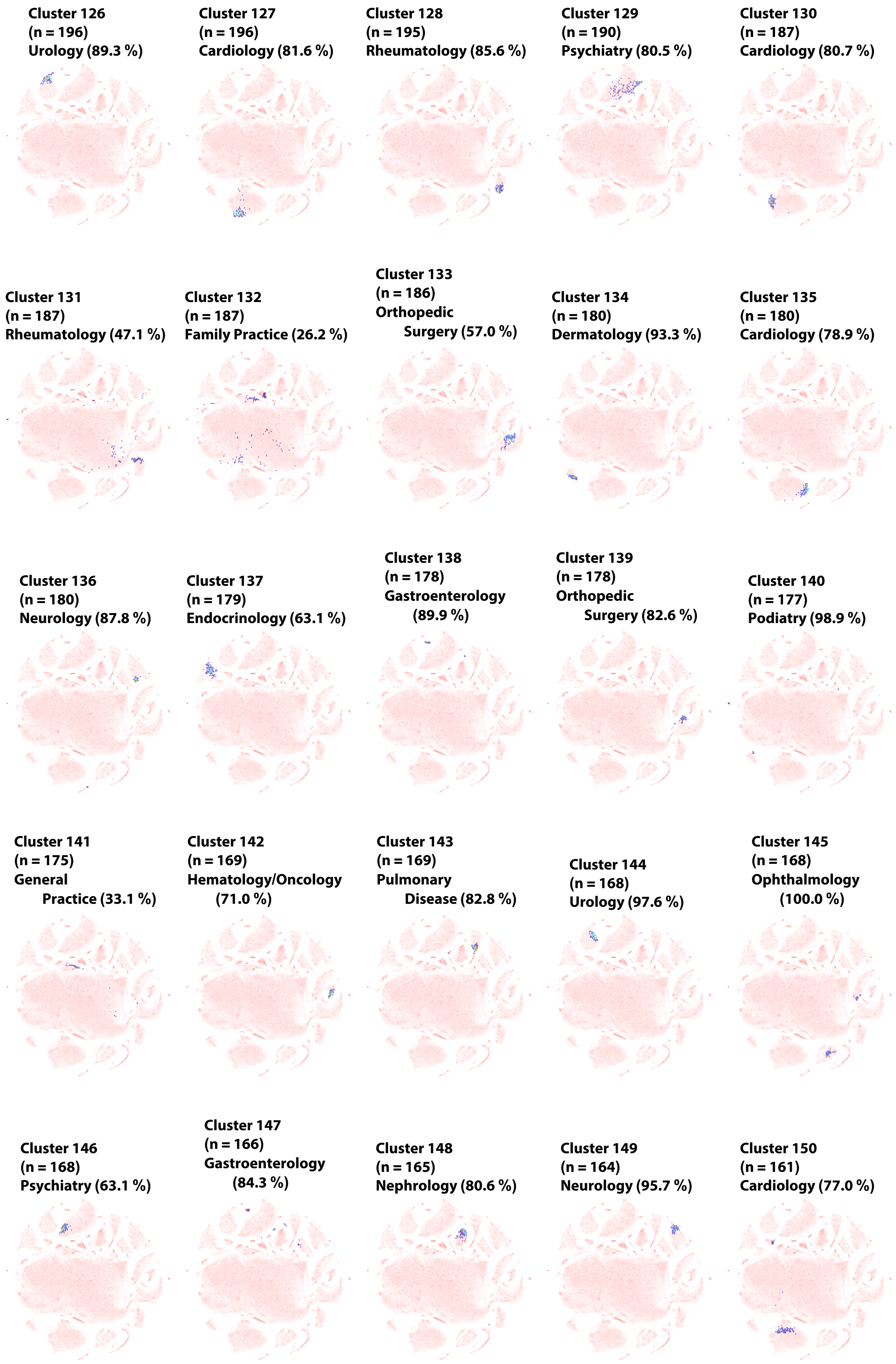
Cluster 126
(n = 196)
Urology (89.3 %)
Cluster 127
(n = 196)
Cardiology (81.6 %)
Cluster 128
(n = 195)
Rheumatology (85.6 %)
Cluster 129
(n = 190)
Psychiatry (80.5 %)
Cluster 130
(n = 187)
Cardiology (80.7 %)
Cluster 131
(n = 187)
Rheumatology (47.1 %)
Cluster 132
(n = 187)
Family Practice (26.2 %)
Cluster 133
(n = 186)
Orthopedic Surgery (57.0 %)
Cluster 134
(n = 180)
Dermatology (93.3 %)
Cluster 135
(n = 180)
Cardiology (78.9 %)
Cluster 136
(n = 180)
Neurology (87.8 %)
Cluster 137
(n = 179)
Endocrinology (63.1 %)
Cluster 138
(n = 178)
Gastroenterology (89.9 %)
Cluster 139
(n = 178)
Orthopedic Surgery (82.6 %)
Cluster 140
(n = 177)
Podiatry (98.9 %)
Cluster 141
(n = 175)
General Practice (33.1 %)
Cluster 142
(n = 169)
Hematology/Oncology (71.0 %)
Cluster 143
(n = 169)
Pulmonary Disease (82.8 %)
Cluster 144
(n = 168)
Urology (97.6 %)
Cluster 145
(n = 168)
Ophthalmology (100.0 %)
Cluster 146
(n = 168)
Psychiatry (63.1 %)
Cluster 147
(n = 166)
Gastroenterology (84.3 %)
Cluster 148
(n = 165)
Nephrology (80.6 %)
Cluster 149
(n = 164)
Neurology (95.7 %)
Cluster 150
(n = 161)
Cardiology (77.0 %)

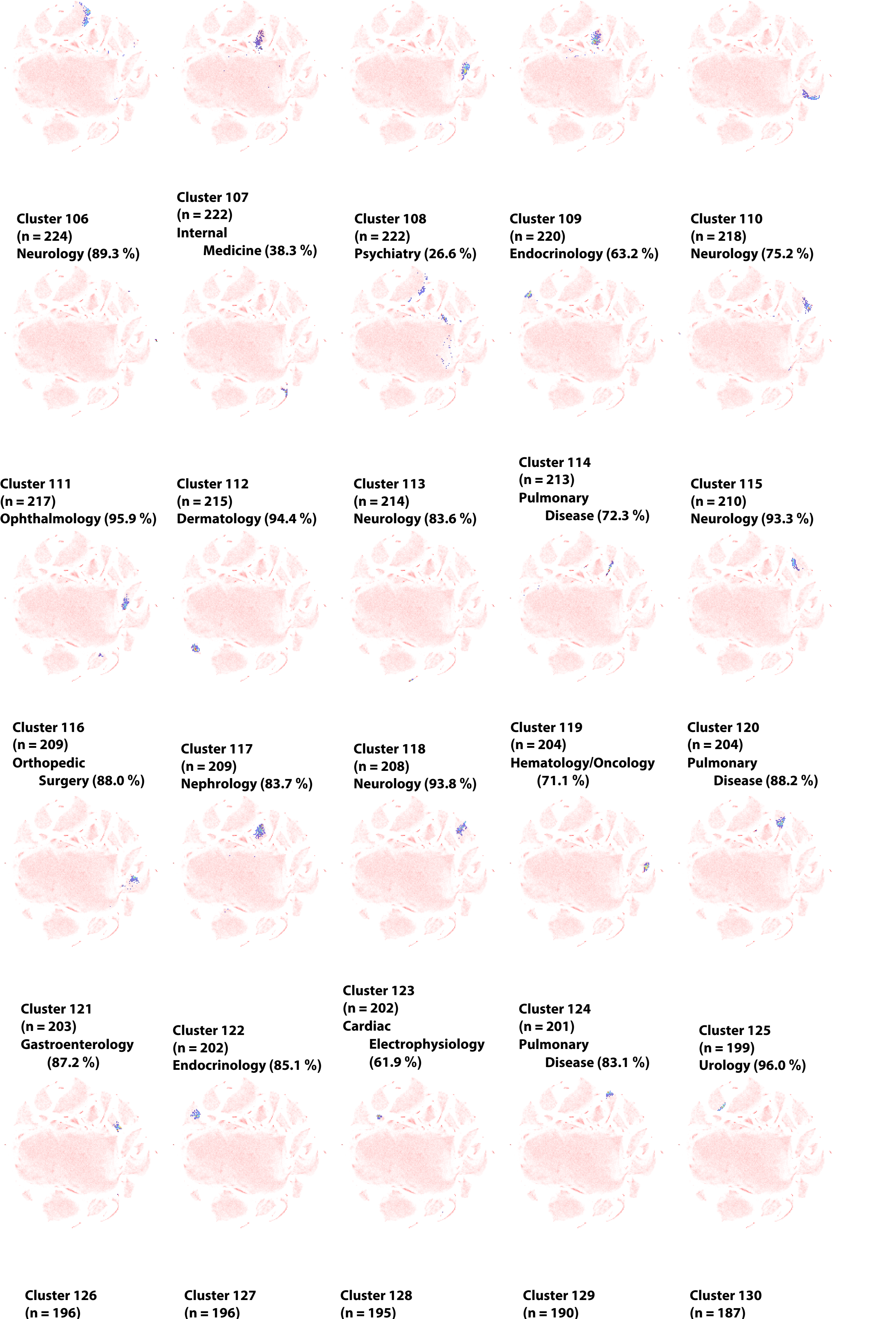
Cluster 106
(n = 224)
Neurology (89.3 %)
Cluster 107
(n = 222)
Internal Medicine (38.3 %)
Cluster 108
(n = 222)
Psychiatry (26.6 %)
Cluster 109
(n = 220)
Endocrinology (63.2 %)
Cluster 110
(n = 218)
Neurology (75.2 %)
Cluster 111
(n = 217)
Ophthalmology (95.9 %)
Cluster 112
(n = 215)
Dermatology (94.4 %)
Cluster 113
(n = 214)
Neurology (83.6 %)
Cluster 114
(n = 213)
Pulmonary Disease (72.3 %)
Cluster 115
(n = 210)
Neurology (93.3 %)
Cluster 116
(n = 209)
Orthopedic Surgery (88.0 %)
Cluster 117
(n = 209)
Nephrology (83.7 %)
Cluster 118
(n = 208)
Neurology (93.8 %)
Cluster 119
(n = 204)
Hematology/Oncology (71.1 %)
Cluster 120
(n = 204)
Pulmonary Disease (88.2 %)
Cluster 121
(n = 203)
Gastroenterology (87.2 %)
Cluster 122
(n = 202)
Endocrinology (85.1 %)
Cluster 123
(n = 202)
Cardiac Electrophysiology (61.9 %)
Cluster 124
(n = 201)
Pulmonary Disease (83.1 %)
Cluster 125
(n = 199)
Urology (96.0 %)
Cluster 126
(n = 196)
Cluster 127
(n = 196)
Cluster 128
(n = 195)
Cluster 129
(n = 190)
Cluster 130
(n = 187)

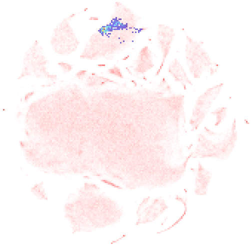

Cluster 81
(n = 284)
Family Practice (43.7 %)

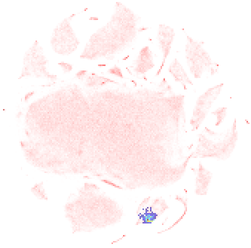

Cluster 82
(n = 279)
Cardiology (89.6 %)

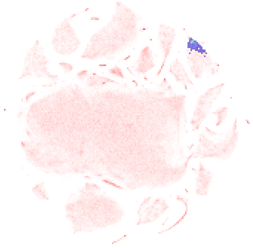

Cluster 83
(n = 279)
Cardiology (89.6 %)

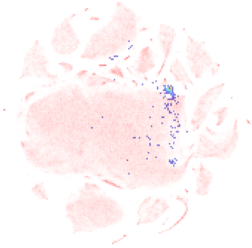

Cluster 84
(n = 277)
Cardiology (89.9 %)

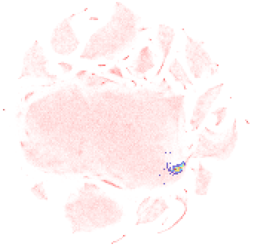

Cluster 85
(n = 275)
Urology (95.6 %)

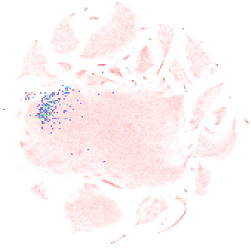

Cluster 86
(n = 274)
Internal Medicine (38.3 %)

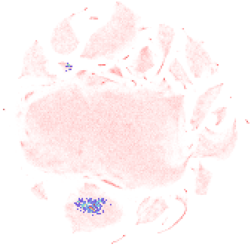

Cluster 87
(n = 272)
Internal Medicine (44.1 %)

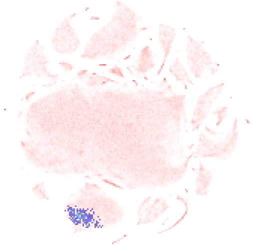

Cluster 88
(n = 271)
Dermatology (94.5 %)

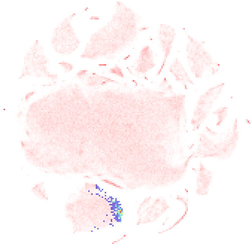

Cluster 89
(n = 270)
Neurology (91.1 %)

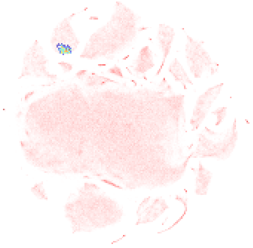

Cluster 90
(n = 268)
Internal Medicine (48.1 %)

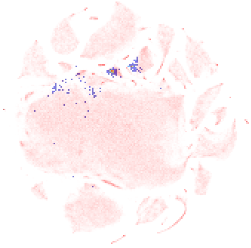

Cluster 91
(n = 267)
Neurology (84.6 %)

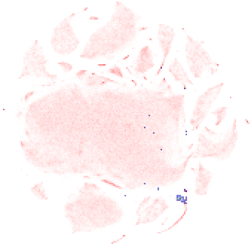

Cluster 92
(n = 262)
Cardiology (49.2 %)

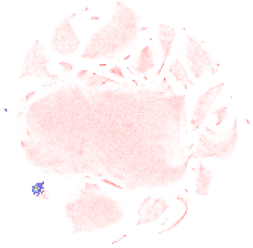

Cluster 93
(n = 257)
Ophthalmology (97.7 %)

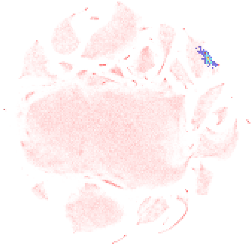

Cluster 94
(n = 249)
Dermatology (86.7 %)

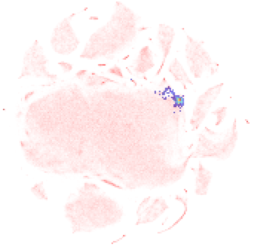

Cluster 95
(n = 247)
Allergy/Immunology (81.8 %)

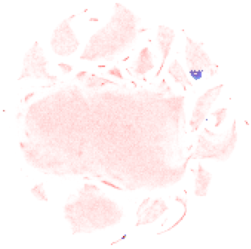

Cluster 96
(n = 246)
Ophthalmology (98.8 %)

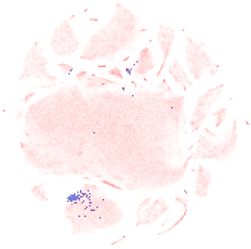

Cluster 97
(n = 243)
Otolaryngology (77.0 %)

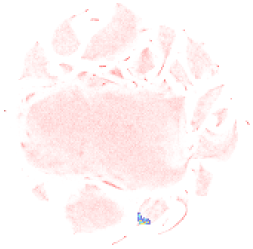

Cluster 98
(n = 238)
Psychiatry (69.7 %)

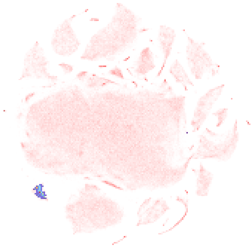

Cluster 99
(n = 235)
Gastroenterology (92.8 %)

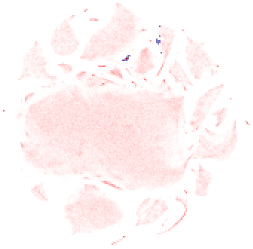

Cluster 100
(n = 233)
Internal Medicine (48.9 %)

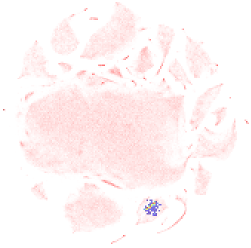

Cluster 101
(n = 232)
Psychiatry (76.3 %)

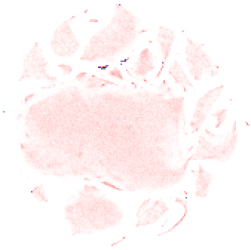

Cluster 102
(n = 231)
Nephrology (77.9 %)

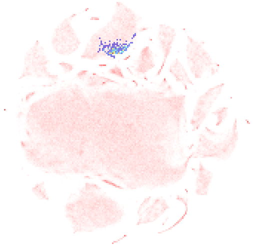

Cluster 103
(n = 228)
Ophthalmology (93.9 %)

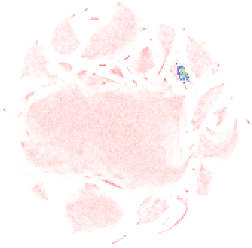

Cluster 104
(n = 226)
Nephrology (81.0 %)

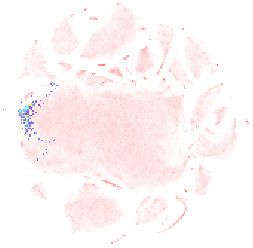

Cluster 105
(n = 225)
Anesthesiology (20.9 %)

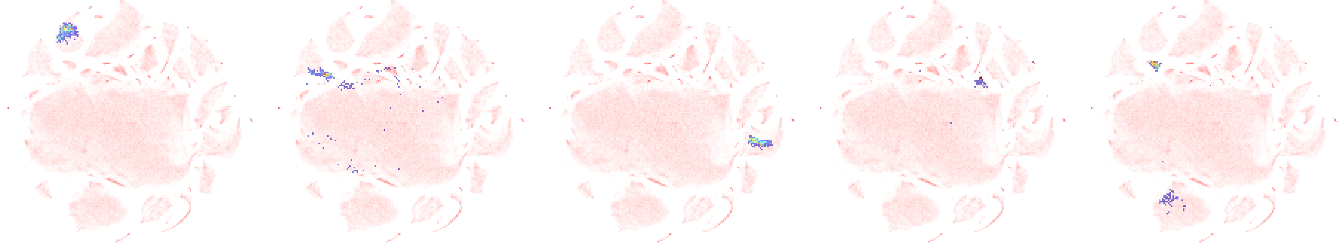

**Cluster 56** (n = 372) Urology (91.7 %)

**Cluster 57** (n = 368) Nurse Practitioner (32.6 %)

**Cluster 58** (n = 367) Family Practice (18.5 %)

**Cluster 59** (n = 361) Psychiatry (74.0 %)

**Cluster 60** (n = 352) Psychiatry (75.6 %)

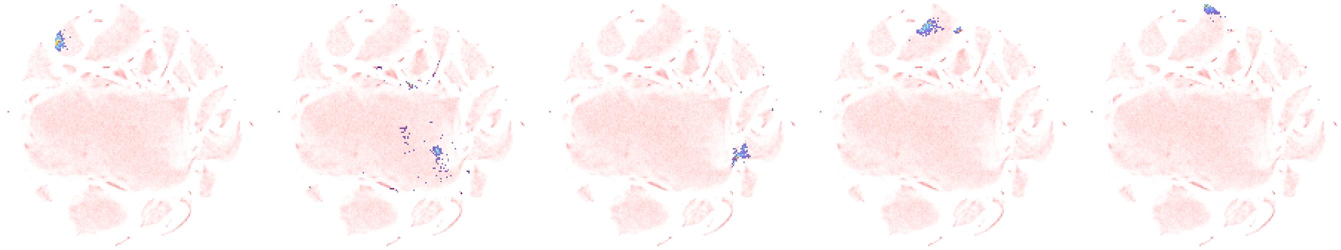

**Cluster 61** (n = 347) Psychiatry (82.4 %)

**Cluster 62** (n = 344) Pulmonary Disease (82.8 %)

**Cluster 63** (n = 335) Internal Medicine (32.5 %)

**Cluster 64** (n = 334) Psychiatry (79.0 %)

**Cluster 65** (n = 332) Psychiatry (73.8 %)

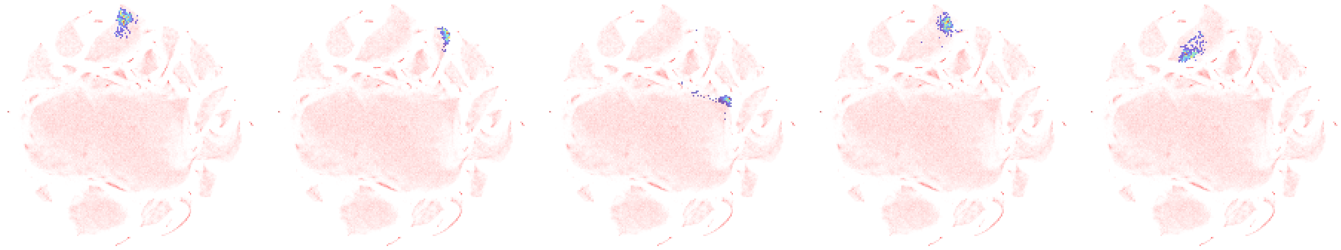

**Cluster 66** (n = 331) Infectious Disease (49.2 %)

**Cluster 67** (n = 327) Hematology/Oncology (68.5 %)

**Cluster 68** (n = 323) Rheumatology (82.0 %)

**Cluster 69** (n = 323) Psychiatry (69.7 %)

**Cluster 70** (n = 319) Physical Medicine and Rehabilitation (24.5 %)

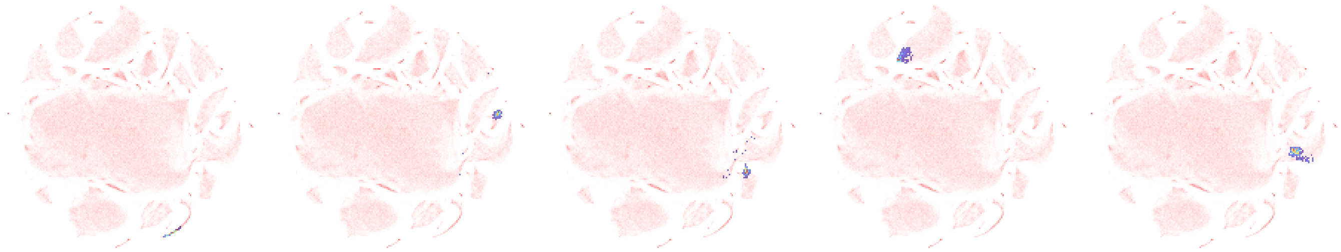

**Cluster 71** (n = 318) Cardiology (83.3 %)

**Cluster 72** (n = 318) Ophthalmology (95.3 %)

**Cluster 73** (n = 311) Ophthalmology (90.0 %)

**Cluster 74** (n = 304) Cardiology (75.3 %)

**Cluster 75** (n = 301) Rheumatology (79.7 %)

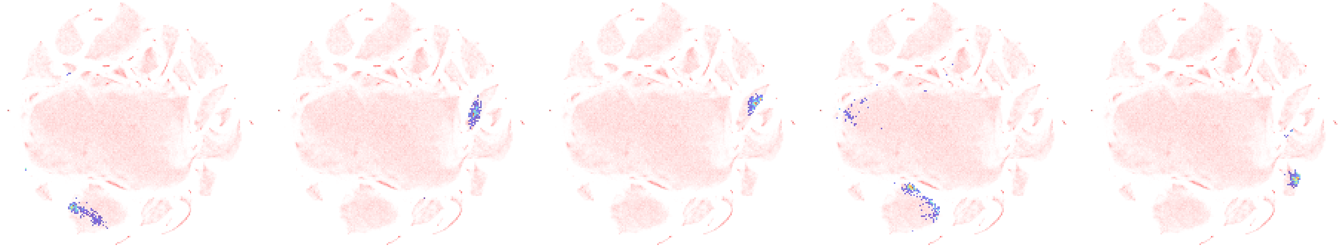

**Cluster 76** (n = 298)

**Cluster 77** (n = 298)

**Cluster 78** (n = 293)

**Cluster 79** (n = 293)

**Cluster 80** (n = 290)

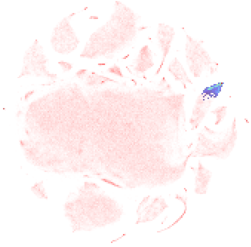
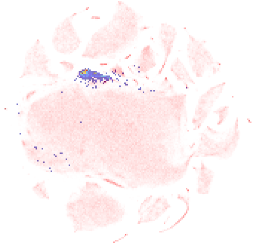
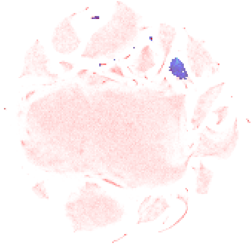
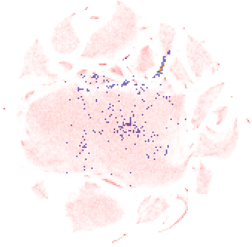
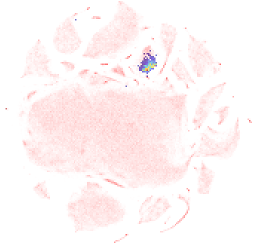

Cluster 31
(n = 692)
Psychiatry (67.9 %)

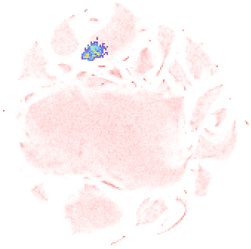

Cluster 32
(n = 676)
Endocrinology (83.7 %)

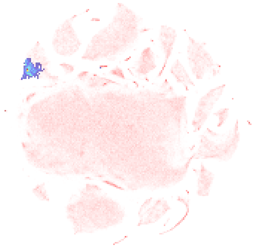

Cluster 33
(n = 668)
Internal Medicine (37.0 %)

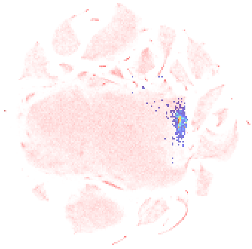

Cluster 34
(n = 660)
Family Practice (36.2 %)

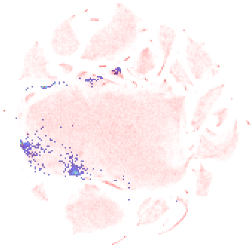

Cluster 35
(n = 649)
Ophthalmology (97.5 %)

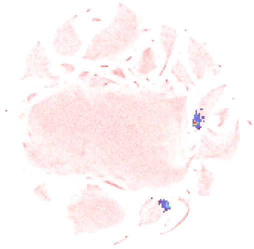

Cluster 36
(n = 608)
Cardiology (84.2 %)

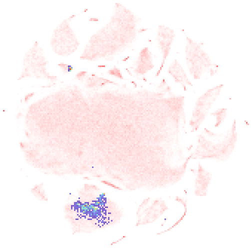

Cluster 37
(n = 596)
Internal Medicine (43.6 %)

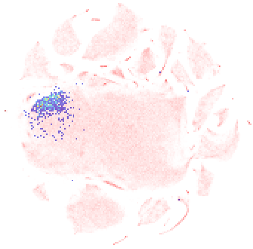

Cluster 38
(n = 563)
Psychiatry (70.0 %)

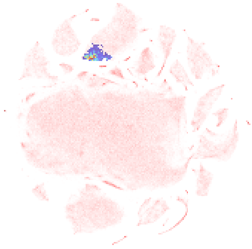

Cluster 39
(n = 544)
Psychiatry (64.2 %)

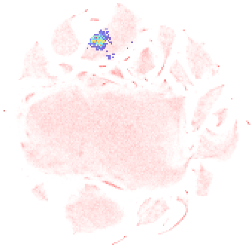

Cluster 40
(n = 534)
Psychiatry (54.3 %)

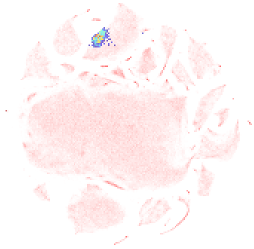

Cluster 41
(n = 526)
Ophthalmology (97.1 %)

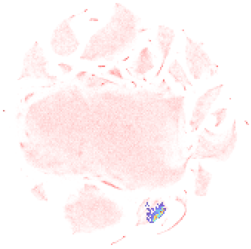

Cluster 42
(n = 503)
Nephrology (86.9 %)

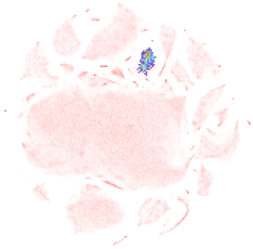

Cluster 43
(n = 502)
Family Practice (42.8 %)

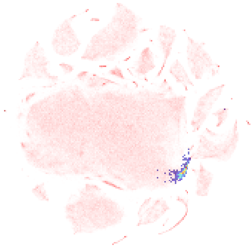

Cluster 44
(n = 495)
Cardiology (68.1 %)

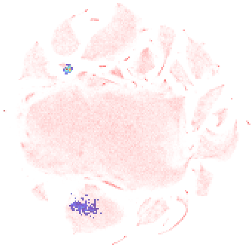

Cluster 45
(n = 477)
Interventional Pain Management (18.9 %)

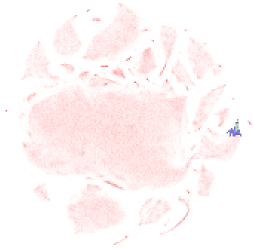

Cluster 46
(n = 472)
Cardiology (85.4 %)

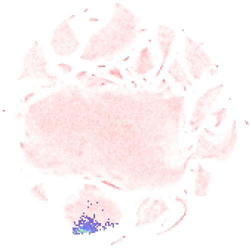

Cluster 47
(n = 462)
Internal Medicine (59.3 %)

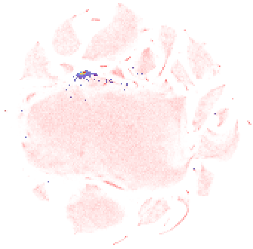

Cluster 48
(n = 457)
Pulmonary Disease (82.3 %)

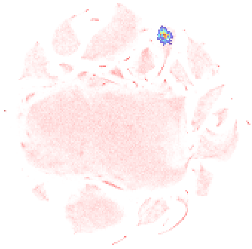

Cluster 49
(n = 455)
Endocrinology (69.9 %)

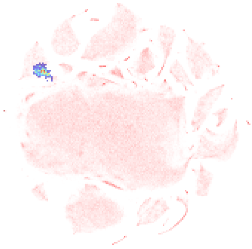

Cluster 50
(n = 440)
Nurse Practitioner (35.5 %)

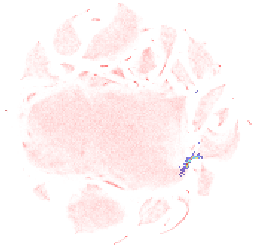

Cluster 51
(n = 436)

Cluster 52
(n = 428)
Internal

Cluster 53
(n = 426)

Cluster 54
(n = 384)
Internal

Cluster 55
(n = 383)

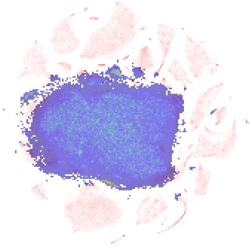
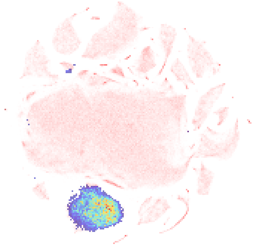
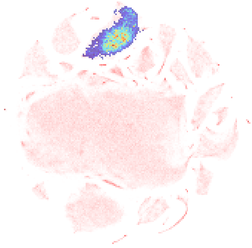
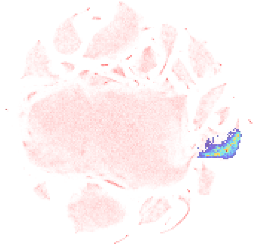
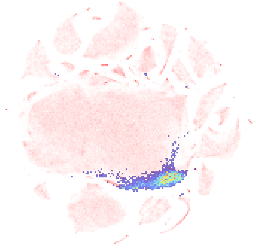

**Cluster 6**
**(n = 2,667)**
**Internal Medicine (53.5 %)**

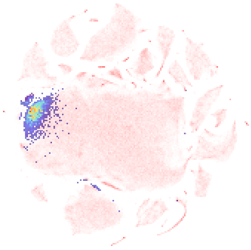

**Cluster 7**
**(n = 2,513)**
**Internal Medicine (51.0 %)**

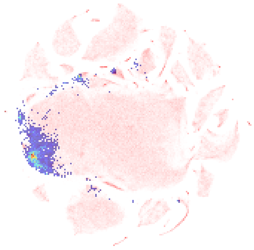

**Cluster 8**
**(n = 1,588)**
**Internal Medicine (47.1 %)**

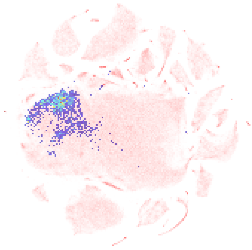

**Cluster 9**
**(n = 1,541)**
**Rheumatology (83.6 %)**

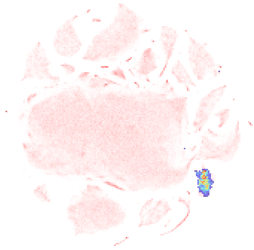

**Cluster 10**
**(n = 1,479)**
**Neurology (93.2 %)**

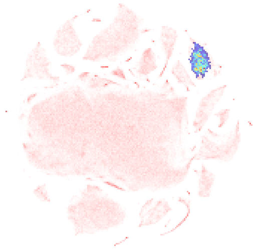

**Cluster 11**
**(n = 1,339)**
**Cardiology (39.7 %)**

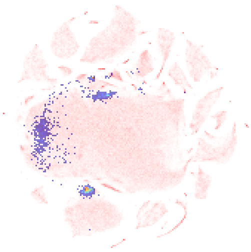

**Cluster 12**
**(n = 1,322)**
**Ophthalmology (91.7 %)**

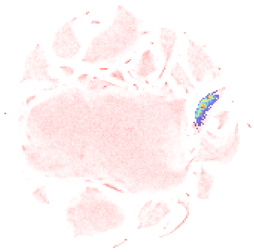

**Cluster 13**
**(n = 1,319)**
**Family Practice (38.7 %)**

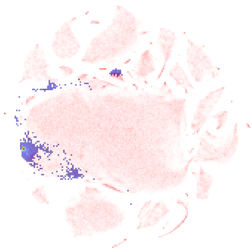

**Cluster 14**
**(n = 1,297)**
**Family Practice (35.6 %)**

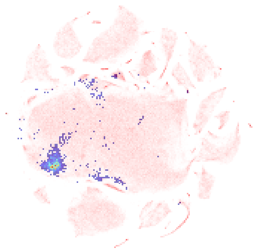

**Cluster 15**
**(n = 1,267)**
**Urology (95.4 %)**

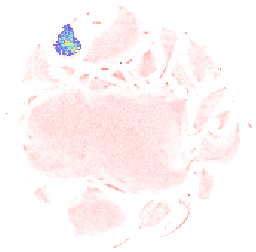

**Cluster 16**
**(n = 1,223)**
**Internal Medicine (54.9 %)**

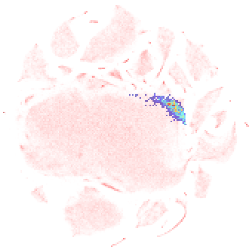

**Cluster 17**
**(n = 1,139)**
**Internal Medicine (44.6 %)**

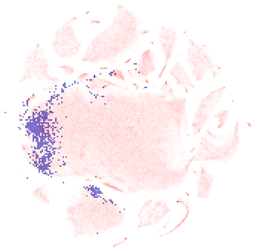

**Cluster 18**
**(n = 1,121)**
**Internal Medicine (46.3 %)**

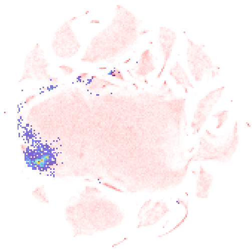

**Cluster 19**
**(n = 1,006)**
**Nephrology (88.4 %)**

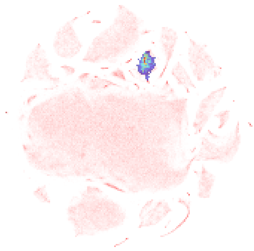

**Cluster 20**
**(n = 1,001)**
**Infectious Disease (38.1 %)**

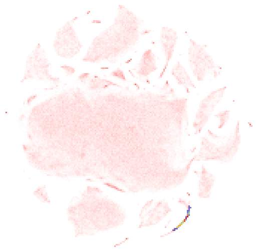

**Cluster 21**
**(n = 983)**
**Psychiatry (79.1 %)**

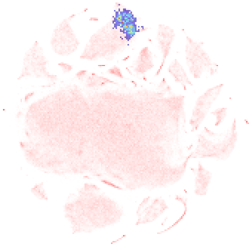

**Cluster 22**
**(n = 900)**
**Pulmonary Disease (80.2 %)**

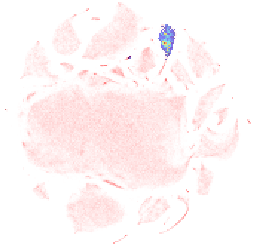

**Cluster 23**
**(n = 882)**
**Endocrinology (80.3 %)**

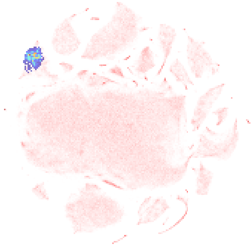

**Cluster 24**
**(n = 880)**
**Family Practice (32.7 %)**

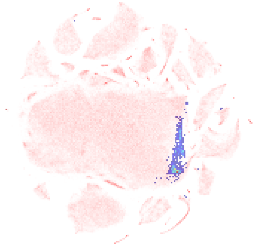

**Cluster 25**
**(n = 794)**
**Cardiology (81.6 %)**

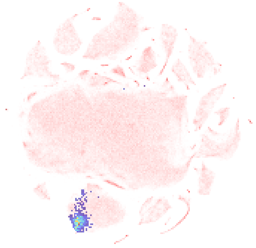

**Cluster 26**
**(n = 782)**
**Ophthalmology (80.9 %)**

**Cluster 27**
**(n = 772)**
**Internal Medicine (43.9 %)**

**Cluster 28**
**(n = 768)**
**Gastroenterology (88.2 %)**

**Cluster 29**
**(n = 762)**
**Pulmonary Disease (41.5 %)**

**Cluster 30**
**(n = 743)**
**Nephrology (73.8 %)**
