# Supplementary material for: Visualizing nationwide variation in medicare Part D prescribing patterns
Source: BMC Med Inform Decis Mak. 2018 Nov 19;18:103. doi: 10.1186/s12911-018-0670-2 (PMC6245567; doi:10.1186/s12911-018-0670-2)
Supplement: Supplementary file 9 — Figure S7. Characteristics of core-based statistical areas (CBSA). 52 CBSAs are listed that have July 2012 population estimates greater than 1,000,000 residents. See Methods for data sources. (PDF 24.2 kb) [file 12911_2018_670_MOESM9_ESM.pdf]

CBSA Code	CBSA Name	IATA Code	July 2012 Pop. (Est.)	# of Part D Enrollees	% of Part D Enrolled	# of Providers	Enrollees / Provider
35620	New York-Newark-Jersey City, NY-NJ-PA	JFK	19837753	1924589	9.70	83578	23.03
31080	Los Angeles-Long Beach-Anaheim, CA	LAX	13037045	1237148	9.49	39852	31.04
16980	Chicago-Naperville-Elgin, IL-IN-WI	ORD	9514059	760387	7.99	32654	23.29
19100	Dallas-Fort Worth-Arlington, TX	DFW	6702801	439555	6.56	17769	24.74
26420	Houston-The Woodlands-Sugar Land, TX	IAH	6175466	390948	6.33	16688	23.43
37980	Philadelphia-Camden-Wilmington, PA-NJ-DE-MD	PHL	6019533	634298	10.54	26426	24.00
47900	Washington-Arlington-Alexandria, DC-VA-MD-WV	IAD	5862594	270101	4.61	17280	15.63
33100	Miami-Fort Lauderdale-West Palm Beach, FL	MIA	5763282	716788	12.44	19036	37.65
12060	Atlanta-Sandy Springs-Roswell, GA	ATL	5454429	386486	7.09	14061	27.49
14460	Boston-Cambridge-Newton, MA-NH	BOS	4642095	427150	9.20	24349	17.54
41860	San Francisco-Oakland-Hayward, CA	SFO	4454159	459868	10.32	15973	28.79
40140	Riverside-San Bernardino-Ontario, CA	SBD	4342332	389086	8.96	8783	44.30
38060	Phoenix-Mesa-Scottsdale, AZ	PHX	4327632	388969	8.99	13444	28.93
19820	Detroit-Warren-Dearborn, MI	DTW	4292832	481562	11.22	17416	27.65
42660	Seattle-Tacoma-Bellevue, WA	SEA	3552591	296363	8.34	13806	21.47
33460	Minneapolis-St. Paul-Bloomington, MN-WI	MSP	3422417	369252	10.79	11725	31.49
41740	San Diego-Carlsbad, CA	SAN	3176138	284797	8.97	9195	30.97
45300	Tampa-St. Petersburg-Clearwater, FL	TPA	2845178	368592	12.95	9474	38.91
41180	St. Louis, MO-IL	STL	2796506	297722	10.65	9904	30.06
12580	Baltimore-Columbia-Towson, MD	BWI	2753922	211514	7.68	12453	16.98
19740	Denver-Aurora-Lakewood, CO	DEN	2646694	207751	7.85	9636	21.56
38300	Pittsburgh, PA	PIT	2360989	370610	15.70	10675	34.72
16740	Charlotte-Concord-Gastonia, NC-SC	CLT	2294990	195795	8.53	6696	29.24
38900	Portland-Vancouver-Hillsboro, OR-WA	PDX	2289038	236186	10.32	9215	25.63
41700	San Antonio-New Braunfels, TX	SAT	2234494	172532	7.72	6456	26.72
36740	Orlando-Kissimmee-Sanford, FL	MCO	2223456	199491	8.97	6236	31.99
40900	Sacramento--Roseville--Arden-Arcade, CA	SMF	2193927	221537	10.10	6712	33.01
17140	Cincinnati, OH-KY-IN	CVG	2129309	241490	11.34	6964	34.68
17460	Cleveland-Elyria, OH	CLE	2064739	287814	13.94	10105	28.48
28140	Kansas City, MO-KS	MCI	2038690	183868	9.02	6919	26.57
29820	Las Vegas-Henderson-Paradise, NV	LAS	1997659	157856	7.90	4504	35.05
18140	Columbus, OH	CMH	1944937	219723	11.30	7563	29.05
26900	Indianapolis-Carmel-Anderson, IN	IND	1929207	165049	8.56	7561	21.83
41940	San Jose-Sunnyvale-Santa Clara, CA	SJC	1892894	166513	8.80	6429	25.90
12420	Austin-Round Rock, TX	AUS	1835110	97772	5.33	4651	21.02
34980	Nashville-Davidson--Murfreesboro--Franklin, TN	BNA	1726759	155511	9.01	6923	22.46
47260	Virginia Beach-Norfolk-Newport News, VA-NC	ORF	1698410	112482	6.62	5042	22.31
39300	Providence-Warwick, RI-MA	PVD	1601160	196891	12.30	6120	32.17
33340	Milwaukee-Waukesha-West Allis, WI	MKE	1566182	157058	10.03	6335	24.79
27260	Jacksonville, FL	JAX	1378040	120186	8.72	4831	24.88
32820	Memphis, TN-MS-AR	MEM	1340739	119186	8.89	3779	31.54
36420	Oklahoma City, OK	OKC	1297397	100874	7.78	4520	22.32
31140	Louisville/Jefferson County, KY-IN	SDF	1251538	140432	11.22	4789	29.32
40060	Richmond, VA	RIC	1232954	109493	8.88	4514	24.26
35380	New Orleans-Metairie, LA	MSY	1227656	139039	11.33	5386	25.81
25540	Hartford-West Hartford-East Hartford, CT	BDL	1214503	134859	11.10	5816	23.19
39580	Raleigh, NC	RDU	1188504	75253	6.33	3050	24.67
13820	Birmingham-Hoover, AL	BHM	1134915	126250	11.12	4404	28.67
15380	Buffalo-Cheektowaga-Niagara Falls, NY	BUF	1133767	152892	13.49	4800	31.85
41620	Salt Lake City, UT	SLC	1123943	74890	6.66	4143	18.08
40380	Rochester, NY	ROC	1082375	152465	14.09	5066	30.10
24340	Grand Rapids-Wyoming, MI	GRR	1005493	113412	11.28	3626	31.28
