# Supplementary material for: Visualizing nationwide variation in medicare Part D prescribing patterns
Source: BMC Med Inform Decis Mak. 2018 Nov 19;18:103. doi: 10.1186/s12911-018-0670-2 (PMC6245567; doi:10.1186/s12911-018-0670-2)
Supplement: Supplementary file 10 — Figure S8. t-SNE plots with particular CBSAs highlighted. A. t-SNE plot based on provider by drug matrix (as in Fig. 3a) with providers in Rochester and Oklahoma City annotated (see Fig. 10b). B. t-SNE plot based on drug class by provider matrix (as in 3b) with providers in Miami and Boston annotated (see Fig. 10e). (PDF 1010 kb) [file 12911_2018_670_MOESM10_ESM.pdf]

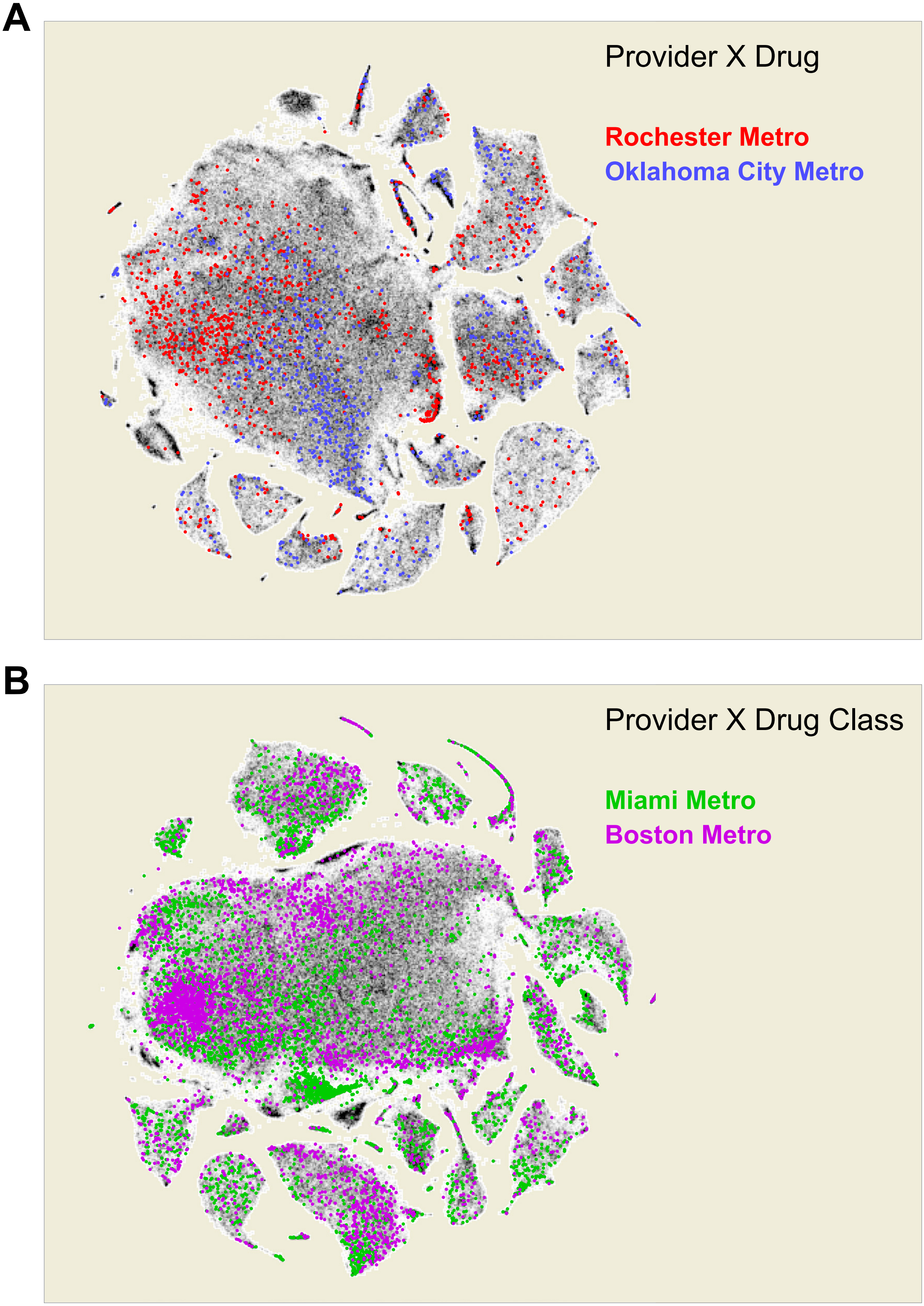
A
Provider X Drug
Rochester Metro
Oklahoma City Metro
B
Provider X Drug Class
Miami Metro
Boston Metro
